# Ag(I)-Induced C–C Reductive Elimination from Pt(II): Role of Ag(I) and Counteranion

**DOI:** 10.1021/acs.organomet.6c00318

**Published:** 2026-10-15

**Authors:** Md Asmaul Hoque, Zoë M. Gehman, Farzad Bastani, Jonah G. Clark, Kevin P. Quirion, Aakashi Jain, Diane A. Dickie, Amrit Venkatesh, Petra Reinke, Daniel H. Ess, T. Brent Gunnoe

**Affiliations:** † Department of Chemistry, 2358 University of Virginia, Charlottesville, Virginia 22904, United States; ‡ Department of Materials Science and Engineering, University of Virginia, Charlottesville, Virginia 22904, United States; § Department of Chemistry and Biochemistry, 6756 Brigham Young University, Provo, Utah 84604, United States

## Abstract

Transition-metal-catalyzed C–C bond formation, including C–H functionalization reactions, often benefits from Lewis acid additives, among which Ag­(I) salts are widely used in Pd-catalyzed transformations. Despite this, direct experimental evidence defining the mechanistic role of Lewis acid additives remains limited. Herein, we investigate the role of Ag­(I) additives in C–C bond formation using (6-Fp)­PtPh_2_ (**1**) (6-Fp = 8,8′-(1,2-phenylene)­diquinoline) as the starting complex. The reaction of (6-Fp)­PtPh_2_ with different Ag­(I) compounds gives significantly different yields for the formation of biphenyl. Unexpectedly, our combined experimental, including ^195^Pt NMR spectroscopy and X-ray photoelectron spectroscopy, and computational studies reveal that Ag­(I) becomes incorporated into the Pt bonding framework to form multimetallic Pt/Ag intermediates that can promote reductive elimination. The Ag­(I) counteranion strongly influences the structure and reactivity of these intermediates, giving the rate trend AgTFA ≈ AgOTf > AgNO_3_ for the formation of biphenyl. These findings provide direct evidence for multimetallic cooperation involving Ag­(I) in C–C bond formation.

## Graphical abstract

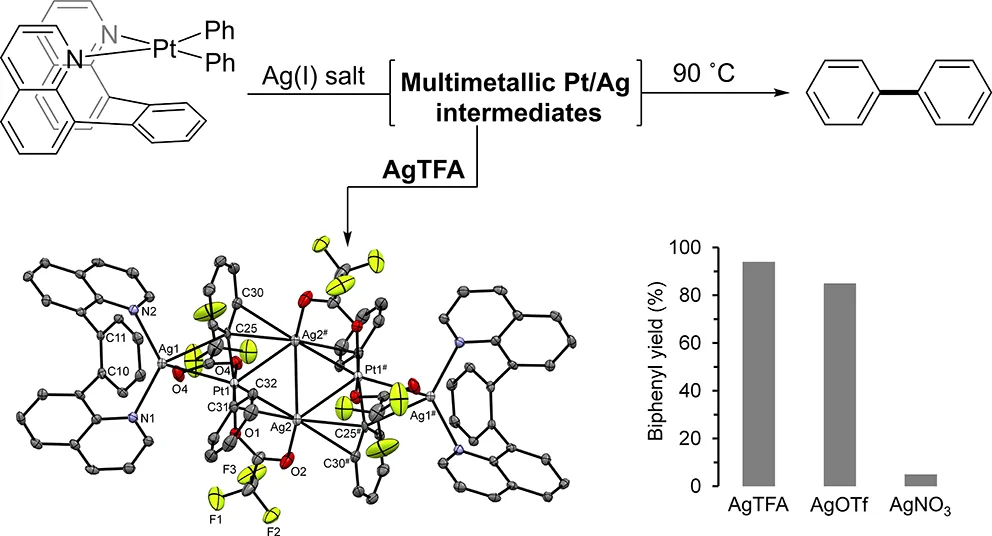

## Introduction

Transition-metal-catalyzed C–C coupling reactions provide a powerful strategy for the construction of carbon–carbon bonds and often enable streamlined access to complex molecular architectures. These transformations have become central to modern synthetic chemistry and are widely used in pharmaceutical and chemical industries.^1^ In many of these catalytic processes, additives such as Lewis or Brønsted acids, are used to achieve optimal reactivity and selectivity (Scheme 1).^2^ Such additives can profoundly influence catalytic activity by activating substrates and/or modulating catalyst speciation, reaction pathways, and key elementary steps.

**Scheme 1:**
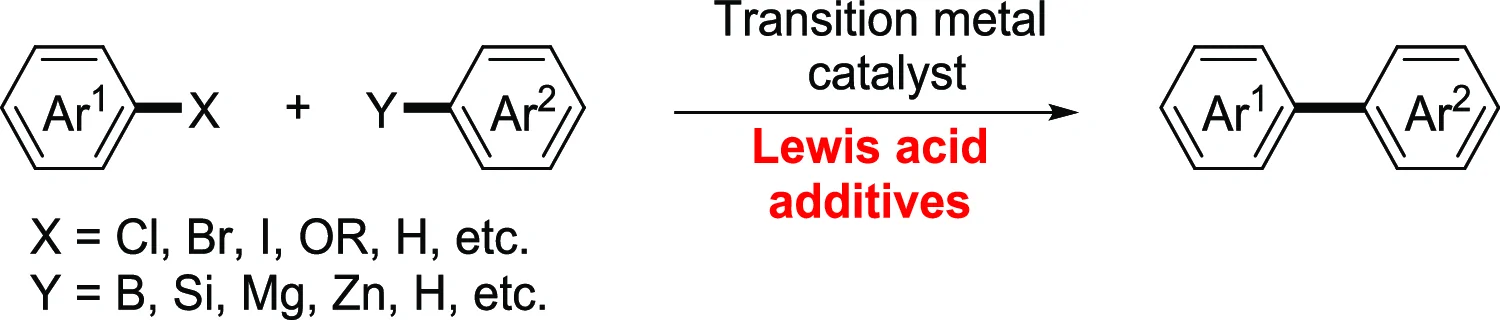
General Reaction Scheme for C–C Coupling Reactions

Among the various additives used to enhance catalysis, Ag­(I) salts have been frequently employed in combination with transition-metal catalysts including Ir, Rh, Co, and Pd in numerous C–H functionalization reactions.^3^ Traditionally, Ag­(I) salts have been invoked to serve several roles including: (i) acting as terminal oxidants to regenerate an active catalyst, (ii) functioning as Lewis acids that interact with substrates or catalytic intermediates, and/or (iii) abstracting halides from catalyst precursors due to the strong halophilicity of Ag­(I). Beyond these conventional roles, computational studies have suggested that Ag­(I) salts may participate directly in the active site through the formation of heterobimetallic complexes with transition metals. In particular, Pd–Ag bimetallic intermediates (Scheme 2, TS 3) have been proposed to lower the activation barriers for key steps such as oxidative addition, concerted metalation–deprotonation (CMD), and reductive elimination.^4^ Despite these proposals, direct experimental evidence defining the mechanistic role of Ag­(I) in these transformations remains scarce.

**Scheme 2:**
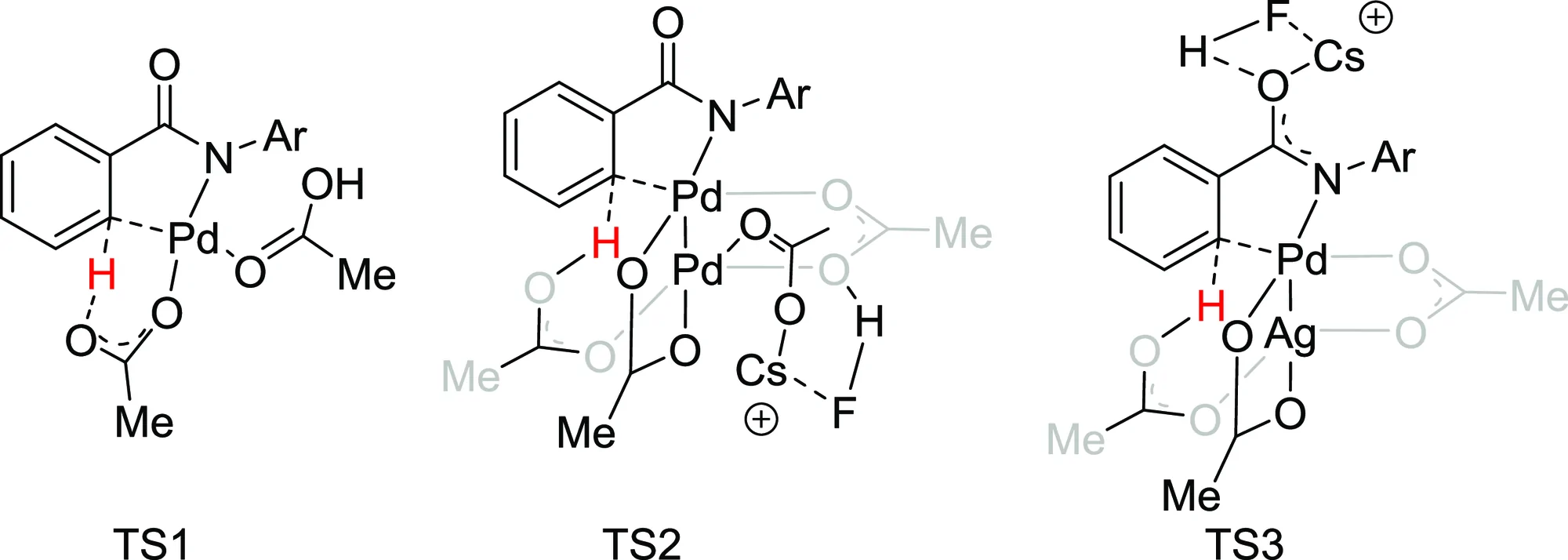
Previously Proposed Formation of Pd–Ag Bimetallic Complex in Pd-Catalysis (Computational Study Only)

Our group has reported a series of arene alkenylation reactions catalyzed by late transition metals including Rh, Ir, and Pd using Cu­(II) carboxylates as oxidants. Combined experimental and computational studies revealed that likely the Cu­(II) oxidant is not merely a terminal oxidant but can become incorporated into the catalyst framework, forming multimetallic species that actively participate in the catalytic cycle.^5^

Reductive elimination represents a key elementary step in transition-metal catalysis and is responsible for product formation in many C–C and C–heteroatom bond-forming reactions.^6^ However, this step can constitute a kinetic bottleneck in catalytic cycles. For d^8^ transition-metal complexes such as Ni­(II), Pd­(II), and Pt­(II), C–C bond formation is generally proposed to proceed via two principal pathways: (i) direct reductive elimination from the d^8^ metal center or (ii) oxidation to a higher-valent metal species, followed by reductive elimination (Scheme 3). Studies led by the Goldberg^7^ and Goddard^8^ groups on six-coordinate octahedral Pt­(IV) alkyl and aryl complexes have demonstrated that ligand dissociation to generate a coordinatively unsaturated five-coordinate intermediate is often required prior to C–C bond formation. More recently, Sanford,^9^ Mirica,^10^ Chang^11^ and other groups have explored oxidatively induced reductive elimination pathways for several late transition metals for which oxidation of a reactive intermediate to a higher-valent state increases the thermodynamic driving force for C–C bond formation. In contrast, analogous reactivity in Pt complexes remains relatively underdeveloped, and Pt complexes are often considered less effective for promoting C–C bond formation through these pathways.^12^

**Scheme 3:**
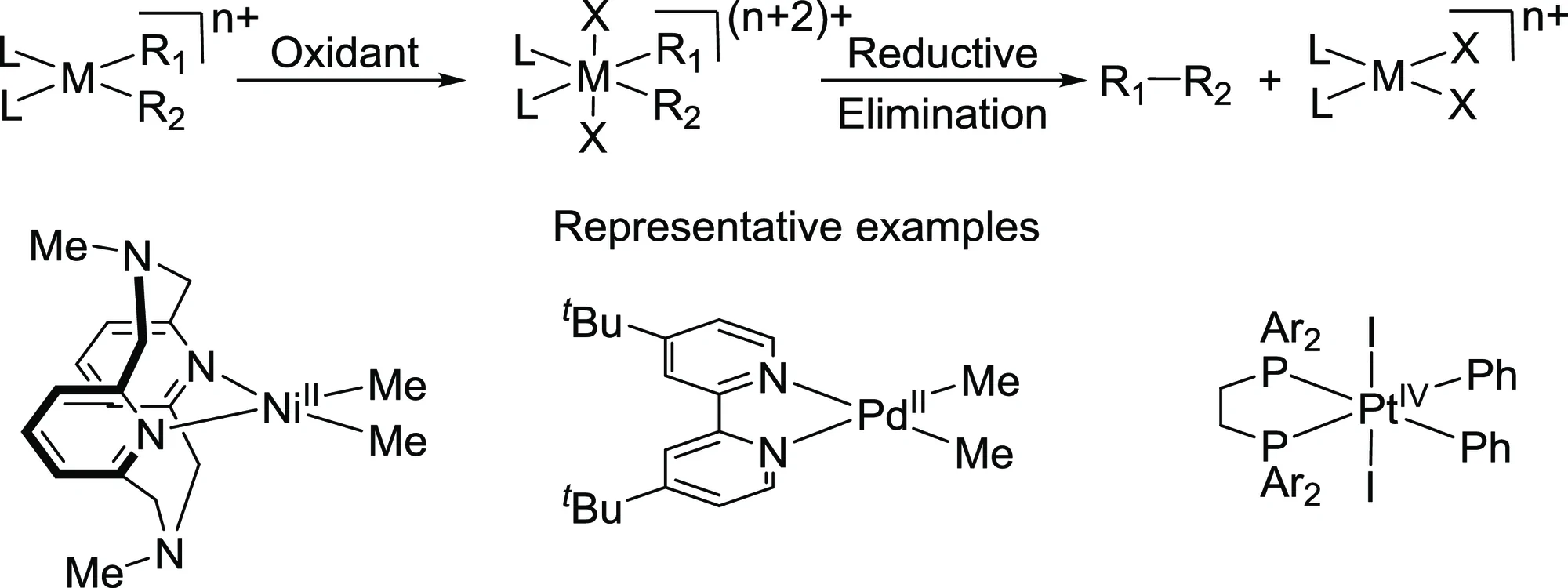
Commonly Proposed Reaction Pathway for Oxidatively Induced Reductive Elimination Reaction Starting from a d^8^ Transition Metal

Building on work by Song and co-workers,^13^ our group has been exploring a family of transition-metal complexes supported by “capping arene” ligands,^14^ which have been applied to a range of transformations (Scheme 4) including catalytic C–H functionalization,^15^ olefin hydrogenation,^16^ stoichiometric C–H activation,^17^ nucleophilic aromatic substitution,^18^ and electrocatalytic water oxidation.^19^ Some of these studies demonstrated that the geometric and electronic properties of the capping arene ligand can modulate the distance and interaction between the metal center and the arene moiety, thereby selectively stabilizing or destabilizing reaction intermediates and transition states. For example, in Rh-catalyzed hydrogenation of styrene to ethylbenzene, the complex (6-Fp)­Rh­(η^2^-C_2_H_4_)Cl (6-Fp = 8,8′-(1,2-phenylene)­diquinoline) produces ethylbenzene nearly twice as rapidly as (5-Fp)­Rh­(η^2^-C_2_H_4_)Cl (5-Fp = 1,2-bis­(N-7-azaindolyl)-benzene).^16^ Density Functional Theory (DFT) calculations indicated that the activation barrier for oxidative addition of dihydrogen is lower by 1.2 kcal/mol for the complex bearing the 6-Fp ligand relative to the 5-Fp analog. This difference was attributed to the shorter Rh–arene interaction for (6-Fp)­Rh­(η^2^-C_2_H_4_)­Cl, which likely stabilizes the Rh­(III) oxidation state relative to the 5-Fp variant. Furthermore, studies of reductive elimination from (Fp)­Rh^III^(Me)­(X)_2_ complexes revealed that the arene moiety can destabilize highly oxidized intermediates through metal–arene interactions, thereby facilitating Me–X bond formation through an S_*N*_2-type pathway.^20^

**Scheme 4:**
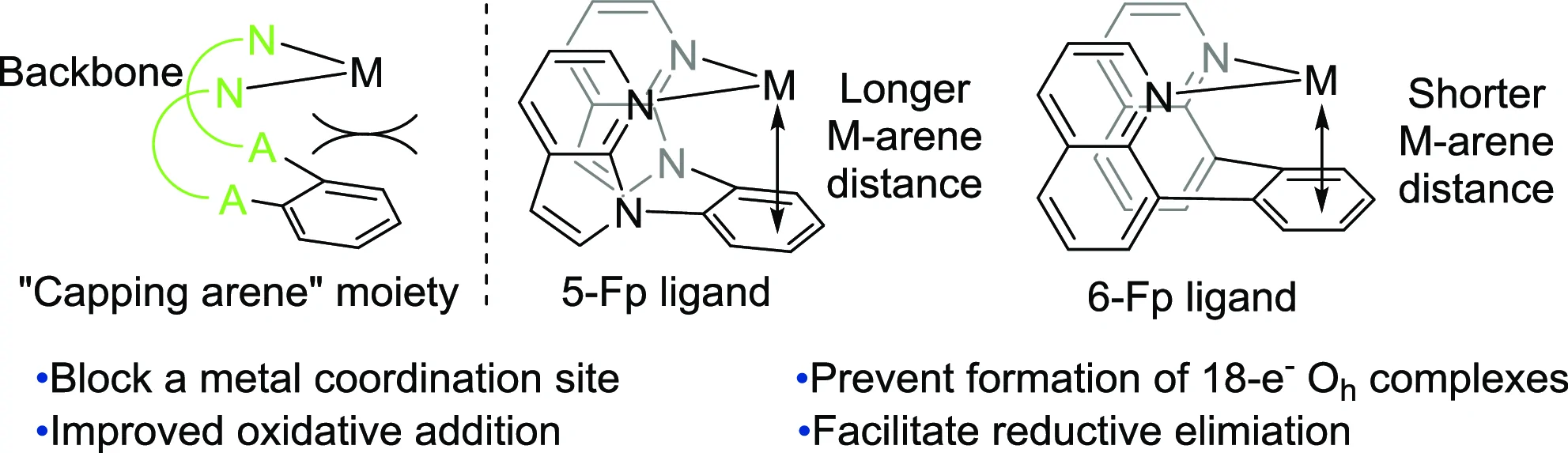
Design of Capping Arene Ligands and Impact of Ligand Structure on Metal-Arene Distances

Motivated by these findings, we became interested in examining how oxidants and capping arene ligands might cooperatively influence oxidatively induced reductive elimination processes leading to carbon–carbon bond formation. Herein, we report the synthesis and reactivity of (6-Fp)­PtPh_2_ (**1**) (6-Fp = 8,8′-(1,2-phenylene)­diquinoline) and reactions with Ag­(I) salts. Our initial goal was to observe and study oxidatively induced reductive elimination to form biphenyl (Scheme 5). However, mechanistic investigations revealed an unexpected role of Ag­(I) in these transformations. Rather than acting solely as an external oxidant to generate Pt­(III) or Pt­(IV) intermediates and Ag(s), Ag­(I) salts react with (6-Fp)­PtPh_2_ to form multimetallic Pt/Ag intermediates prior to reductive elimination of biphenyl. Furthermore, the counteranion of the Ag­(I) salt influences the structure of these Pt/Ag species and consequently dictates the rate of biphenyl formation. These findings, which diverted our original goal of understanding the role of the capping arene ligand, provide direct experimental insight into an unexpected mechanistic role of Ag­(I) additives in transition-metal-mediated C–C bond formation reactions and indicate the possibility of a similar and complicated role for Ag­(I) in catalytic processes as well as other Lewis acids when used to promote catalytic activity.

**Scheme 5:**
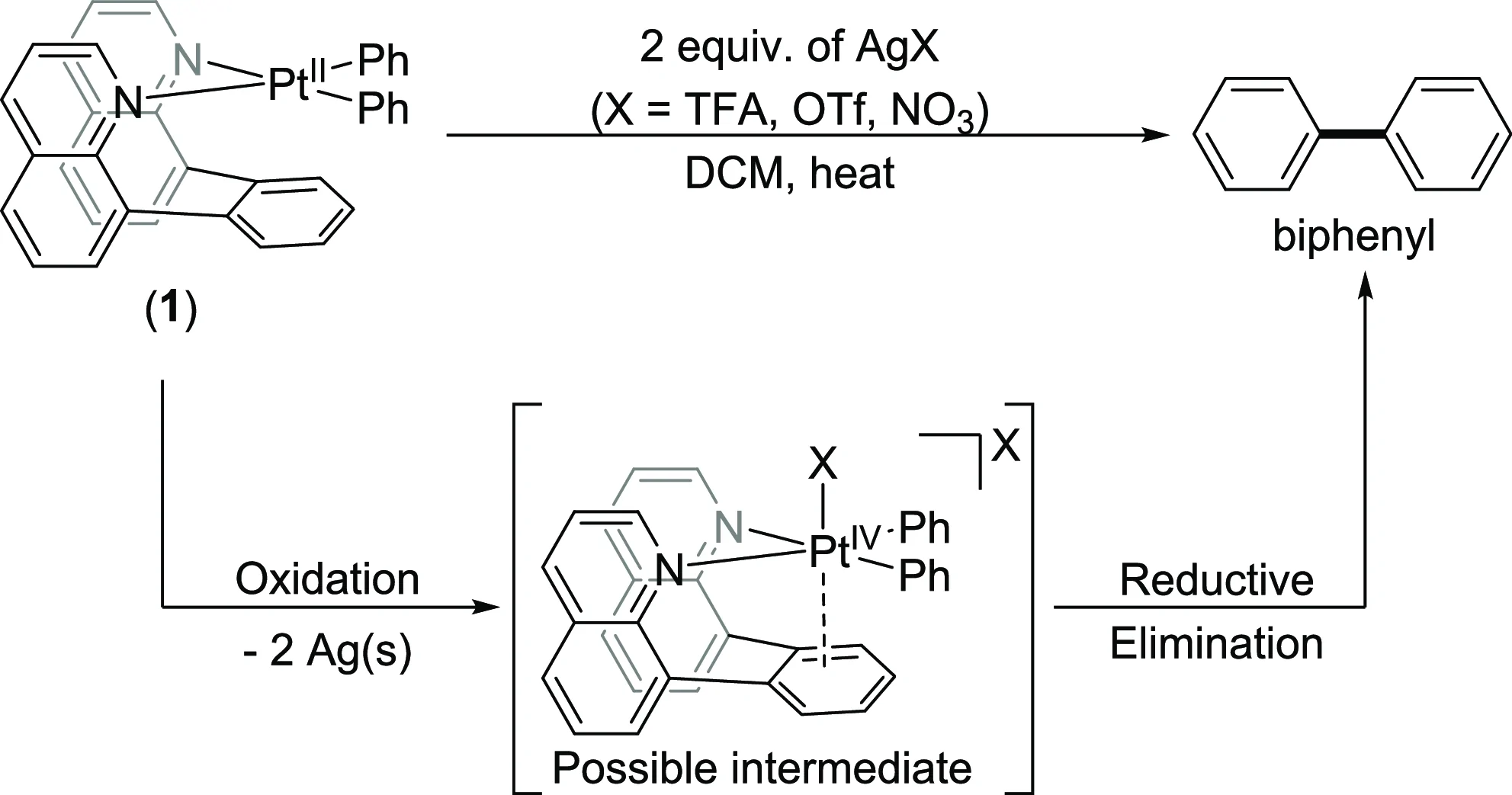
Proposed Pathways for Oxidatively Induced Reductive Elimination of Biphenyl from (6-Fp)­PtPh_2_ (**1**)

## Results and Discussion

### Synthesis and Characterization of Capping Arene-Ligated Pt Complex and Its Reactivity toward Biphenyl Formation

Treatment of 6-Fp with the platinum precursor [(Ph_2_Pt)_2_(μ-SEt_2_)_2_] in diethyl ether at room temperature afforded the orange complex (6-Fp)­PtPh_2_ (**1**) in 72% isolated yield (eq 1). Complex **1** was characterized in both solution and solid states. In the ^1^H NMR spectrum, the ortho-hydrogens of the phenyl groups bound to Pt display strong Pt–H coupling (^3^ *J*_Pt–H_ = 78 Hz, also measured using ^1^H–^195^Pt HSQC, see Section 4.2 in the Supporting Information for ^195^Pt NMR analysis), consistent with previously reported Pt–Ph complexes.^12,21^ Additionally, the ortho-hydrogens of the pyridine units in the Fp ligand show weak interaction with the Pt center (^3^ *J*_Pt–H_ = 15–20 Hz). Single-crystal X-ray diffraction (XRD) analysis (Figure 1) reveals that complex **1** adopts a square-planar geometry around the Pt center. The distances between the Pt center and the closest carbon atoms of the arene moiety are 2.998(4) Å (Pt1–C10) and 3.052(4) Å (Pt1–C15), indicating that the arene ring is positioned in close proximity to the metal center. The C–C bond distances in C6 arene moiety are in the range of 1.374 to 1.419 Å.^22^ We reasoned that the proximity of the C6 arene group and Pt could allow the arene unit to interact with the Pt center, thus enabling more facile oxidation to Pt­(III) or Pt­(IV), compared to similar Pt­(II) complexes that lack a Pt/arene bonding interaction,^14,15,19,20^ which, in turn, might facilitate overall reductive elimination of biphenyl using oxidatively induced C–C bond formation.
1

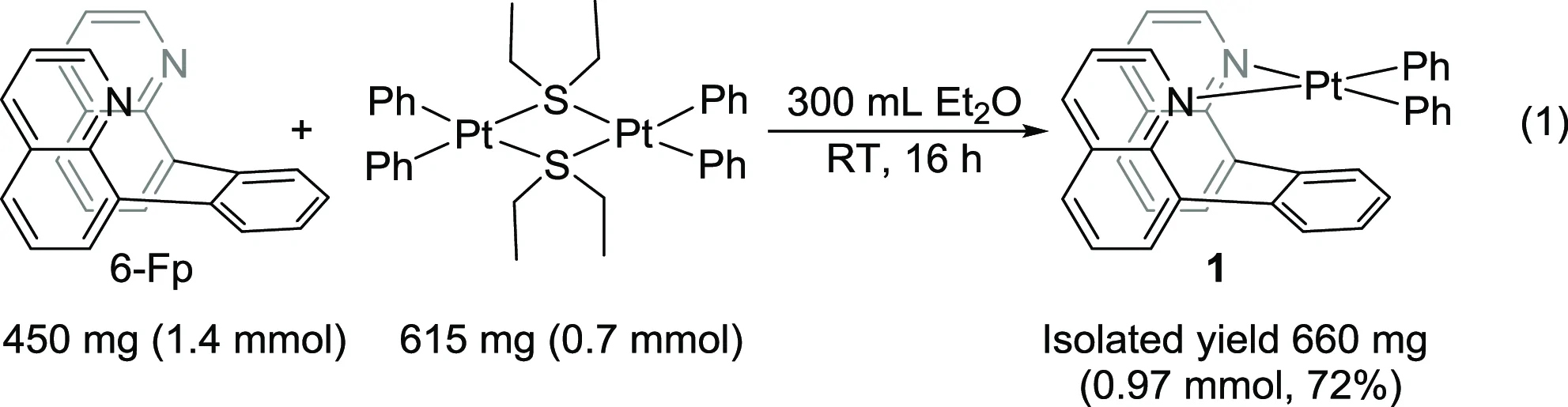




**Figure 1:**
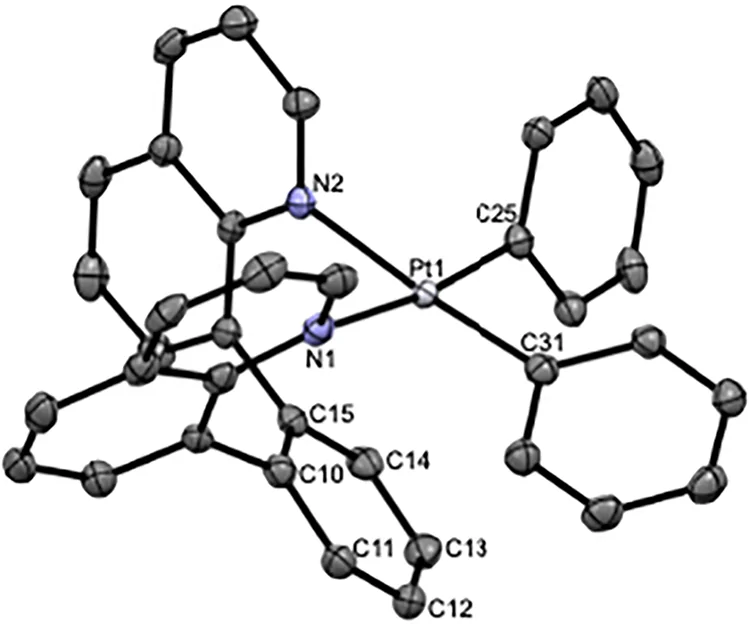
Structure of (6-Fp)­PtPh_2_ (**1**). Ellipsoids are drawn at the 50% probability level. Hydrogen atoms and the minor position of the disordered Pt atoms are omitted for clarity. Selected distances [Å], and angles [deg]: Pt1–N1 2.143(3), Pt1–N2 2.162(3), Pt1–C25 1.985(3), Pt–C31 2.001(3), Pt1–C10 2.998(4), Pt1–C15 3.052(4), C10–C11 1.396(4), C10–C15 1.419(5), C11–C12 1.391(4), C12–C13 1.388(5), C13–C14 1.374(4), C14–C15 1.404(5), N1–Pt–N2 87.54(9), C25–Pt–C31 90.58(12).

Based on literature precedent, we considered that the Pt­(II) center in **1** could be oxidized to Pt­(III) or Pt­(IV) upon treatment with an oxidant. To test this hypothesis, complex **1** was treated with various Ag­(I) salts as oxidants, and biphenyl formation was monitored. In a typical experiment, reactions were performed in a J-Young NMR tube containing 5 mM of **1** and varying equivalents of Ag­(I) salts using 1,3,5-trimethoxybenzene as an internal standard in separate capillary in CD_2_Cl_2_. The reaction mixtures were heated at 90 °C for 4 h, and the results are summarized in Table 1 (See Supporting Information, Figures S3–S5). Biphenyl formation was observed when **1** was treated with AgTFA (TFA = trifluoroacetate), AgOTf (OTF = trifluoromethanesulfonate), and AgNO_3_, albeit with significantly different reactivity and yields. For example, when 2 equiv of Ag­(I) were used, AgTFA afforded biphenyl in 94% yield, whereas AgOTf gave 85% yield and AgNO_3_ produced only 5% yield (Table 1, entries 1–3). When the Ag­(I) loading was reduced to 1 equiv (relative to **1**), biphenyl formation was still observed (49% for AgTFA, 22% for AgOTf, and 3% for AgNO_3_, entries 4–6). Increasing the Ag­(I) loading to 4 equiv did not significantly affect the yield of biphenyl compared to using two equivalents of Ag­(I) salt. Notably, in the case of AgTFA, biphenyl formation was also observed at room temperature, albeit in lower yield (<30%, entries 1,4,7) than at 90 °C, whereas AgOTf and AgNO_3_ produced no detectable biphenyl at room temperature.

**Table 1:**
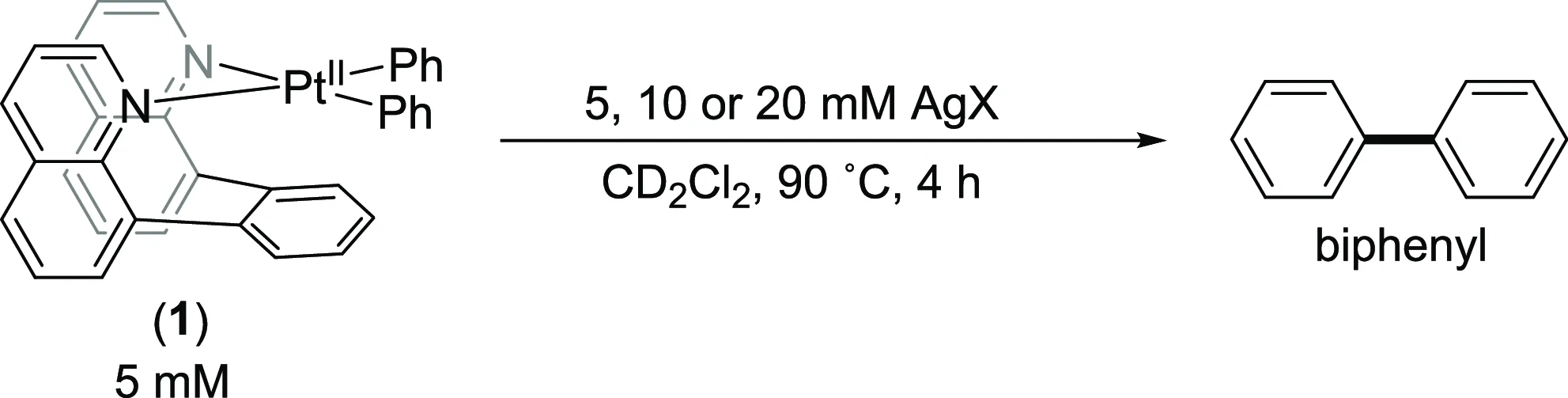
Reactivity Study of Complex 1 for Biphenyl Synthesis Using Various Ag (I) Salts^a^

entry	oxidant	oxidant (equiv)	% biphenyl at RT	% biphenyl at 90 °C
1	AgTFA	2	30(5)	94(3)
2	AgOTf	2	0	85(3)
3	AgNO_3_	2	0	5(1)
4	AgTFA	1	12(2)	49(3)
5	AgOTf	1	0	22(3)
6	AgNO_3_	1	0	3(2)
7	AgTFA	4	25(3)	93(4)
8	AgOTf	4	0	83(4)
9	AgNO_3_	4	0	7(2)

a Reaction conditions: Reactions were carried out in J-Young NMR tubes containing 0.5 mL, 5 mM of complex **1** and 1, 2, or 4 equiv of AgX (X= TFA, OTf, NO_3_) using 1,3,5-trimethoxybenzene as an internal standard at 90 °C for 4 h in CD_2_Cl_2_. Standard deviations are calculated based on a minimum of three independent experiments.

These results indicate that the counteranion of the Ag­(I) salt plays a role in the C–C bond-forming reaction to give biphenyl. The redox potentials of these partially soluble Ag­(I) salts in DCM solvent (0.42 V vs Fc/Fc^+^ for AgTFA, 0.47 V for AgOTf, and 0.30 V for AgNO_3_) are higher than that of the Pt­(II/III) redox couple (0.20 V vs Fc/Fc^+^; see Figure S6 for CV and DPV), suggesting that all three Ag­(I) salts should be capable of oxidizing the Pt center and potentially promote reductive elimination. However, the distinct differences in reactivity observed with the various Ag­(I) compounds suggested that these salts may play a different role beyond merely serving as oxidant. We therefore sought to further investigate the mechanistic role of these Ag­(I) salts in this transformation.

### Reactivity Study of (6-Fp)­PtPh_2_ (1) with Ag­(I) Salts at Room Temperature

To understand the reactions of **1** with different Ag­(I) salts, we sought to characterize potential reactive intermediates formed from the reaction of **1** with the three different Ag­(I) salts. The treatment of **1** with two equivalents of AgTFA, AgOTf, or AgNO_3_ at room temperature in dichloromethane (DCM) afforded three distinct complexes (Scheme 6). We consider the formal oxidation states in **2**–**4** to be Pt­(II) and Ag­(I), although this is, of course, not necessarily a reflection of the actual metal charges. Despite their structural differences, each complex exhibited the same number of proton resonances in their respective ^1^H NMR spectra (Figure S7). ^1^H NMR spectroscopy revealed that the phenyl resonances of complexes **2**–**4** (Figures S8–S16) are shifted to higher chemical shifts relative to those of the parent complex **1**, which is consistent with modification of the coordination environment of Pt upon formation of Pt/Ag interactions. The magnitude of the shift depends on the Ag salt employed. Complex **2**, generated using AgTFA, exhibits the largest increase in chemical shift, whereas complexes **3** and **4**, obtained from AgOTf and AgNO_3_, respectively, show more modest changes (Figure S7). For complexes **3** and **4**, which have similar Pt coordination environments, the trend in chemical shift is consistent with the relative redox potentials of the Ag­(I) salts. This trend correlates with the relative redox potential of the Ag­(I) salts. In all heterometallic species, the Pt–H coupling is diminished or poorly resolved in comparison to that observed for **1**. Furthermore, a rigorous chemical shift assignment of **1** using COSY, NOESY, HSQC and HMBC methods, and comparison of ^1^H–^13^C HSQC spectra of **1** and **2**, clearly illustrate the modifications in the structure due to addition of AgTFA (see Supporting Information, Section 4.2).

**Scheme 6:**
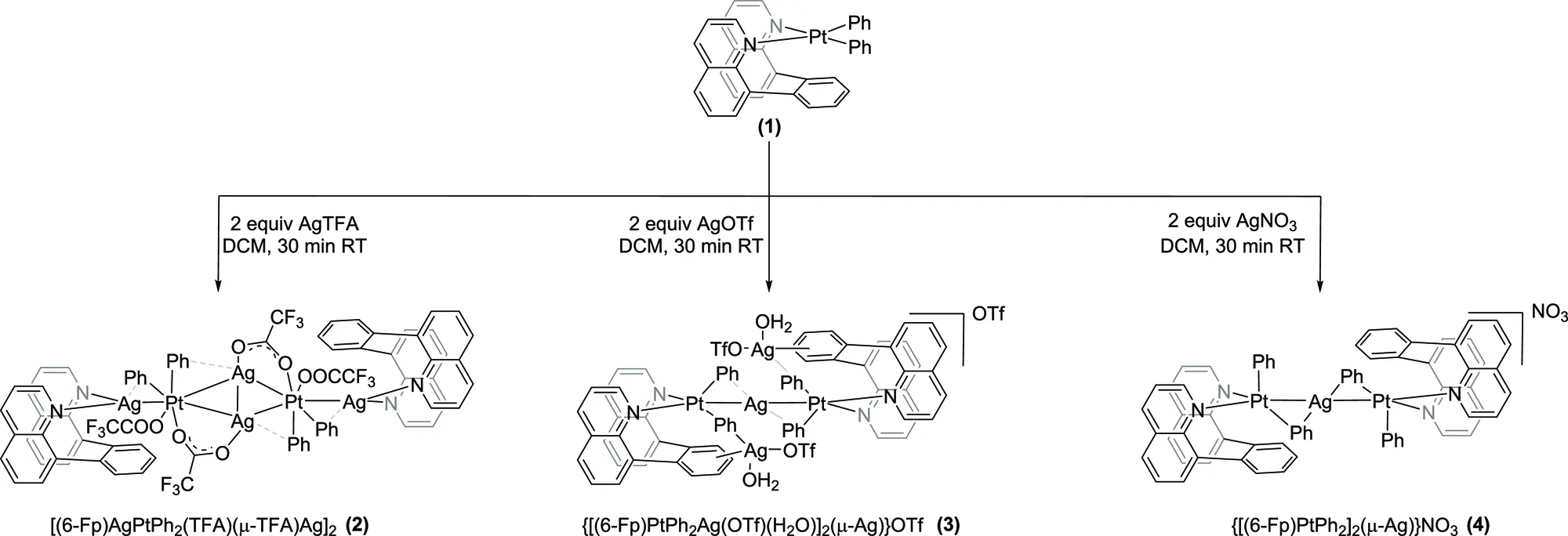
Reaction of (6-Fp)­PtPh_2_ (1) with 2 Equiv of AgTFA, AgOTf or AgNO_3_ at Room Temperature Leads to the Formation of Pt/Ag Multimetallic Complexes: [(6-Fp)­AgPtPh_2_(TFA)­(μ-TFA)­Ag]_2_ (2), {[(6-Fp)­PtPh_2_Ag­(OTf)­(H_2_O)]_2_(*μ*-Ag)}­OTf (3), and {[(6-Fp)­PtPh_2_]_2_(μ-Ag)}­NO_3_, (4), Respectively

To establish the formation of Pt–Ag bimetallic complexes in solution, we obtained ^195^Pt NMR spectra of compounds **1**–**4**. The ^195^Pt NMR spectrum of **1** shows a signal at −2987 ppm. The ^195^Pt NMR chemical shifts of complexes **3** and **4** appear at −2638 and −2627 ppm, respectively, which increase on average by ca. 350 ppm in comparison to **1**, in good agreement with two Pt–Ag dative interactions.^23^ Complex **2** shows a relatively intense signal at ca. −2460 ppm, which indicates a further increase in ^195^Pt chemical shift by ca. 200 ppm, that supports higher number of Pt–Ag interactions, indicated by the single-crystal X-ray structure. A second, weaker ^195^Pt NMR signal was observed at ca. – 2200 ppm; however, the low signal-to-noise ratios prevented the identity of these signals to be unequivocally established. We tentatively assign this second weaker signal to a putative Pt product formed as a result biphenyl elimination from **2** given that biphenyl elimination occurs at room temperature (Table 1). A combination of ^1^H, ^13^C, ^15^N and ^195^Pt NMR spectra supports the formation of the proposed Pt–Ag complexes in solution, in good agreement with the single crystal X-ray diffraction structures (*vide infra*). Single-crystal XRD analysis confirmed the formation of heterometallic Pt/Ag complexes, in which the Ag centers are coordinated directly to the platinum frameworks.

### Isolation and Characterization of the Reactive Intermediates

Reaction of the yellow-orange precursor **1** with AgTFA in DCM at room temperature for 30 min yielded the dark-orange heterometallic complex, [(6-Fp)­AgPtPh_2_(TFA)­(μ-TFA)­Ag]_2_ (**2**), in 81% isolated yield. Single-crystal XRD analysis of crystals grown via slow evaporation from a DCM/hexane mixture revealed a multinuclear Pt/Ag architecture with a stoichiometric Pt/Ag ratio of 2:4 (Figure 2). The most salient structural feature of **2** is the transmetalation of a silver­(I) center into the 6-Fp ligand scaffold. One Ag­(I) ion displaces the platinum center from the 6-Fp coordination environment, supported by a Pt–Ag bond and a weak η^1^-interaction with a phenyl ipso-carbon. Concurrently, the Pt center remains ligated to the phenyl groups but expands its coordination sphere to accommodate two additional ligand-unsupported Ag­(I) ions and two TFA ligands. One TFA unit bridges to a ligand-unsupported Ag­(I) center, resulting in a distorted, seven-coordinate capped octahedral geometry around the Pt center. The bond distance between the ligated Ag and Pt (Ag1–Pt1 = 2.7884(3) Å) is shorter than that of the unligated Ag–Pt interaction (Pt1–Ag2 = 2.9449(3) Å) suggesting a covalent-like interaction in the former. Upon complexation, the Ag–N­(6-Fp) and Pt–C­(Ph) distances in **2** undergo elongation relative to complex **1** (Table 2). Furthermore, the C_Ph_–Pt–C_Ph_ angle (C25–Pt1–C31) widens from 90.59(12)° for **1** to 93.74(17)° in **2**, reflecting the steric and electronic demands of the multinuclear core. The coordination modes of the silver centers are notably distinct: while the ligated Ag1 exhibits a weak η^1^ interaction with the phenyl ipso-carbon (Ag1–C25 = 2.699(4) Å), the nonligated Ag2 displays a more pronounced η^2^-interaction with the phenyl rings (Ag2–C31 = 2.449(4) Å, Ag2–C25^#1^ = 2.521(4) Å). Additionally, short Ag2–Ag2^#1^ distances of 2.9905(6) Å indicate argentophilic interactions, which likely stabilize the assembly of the Pt_2_Ag_4_ core.

**Figure 2:**
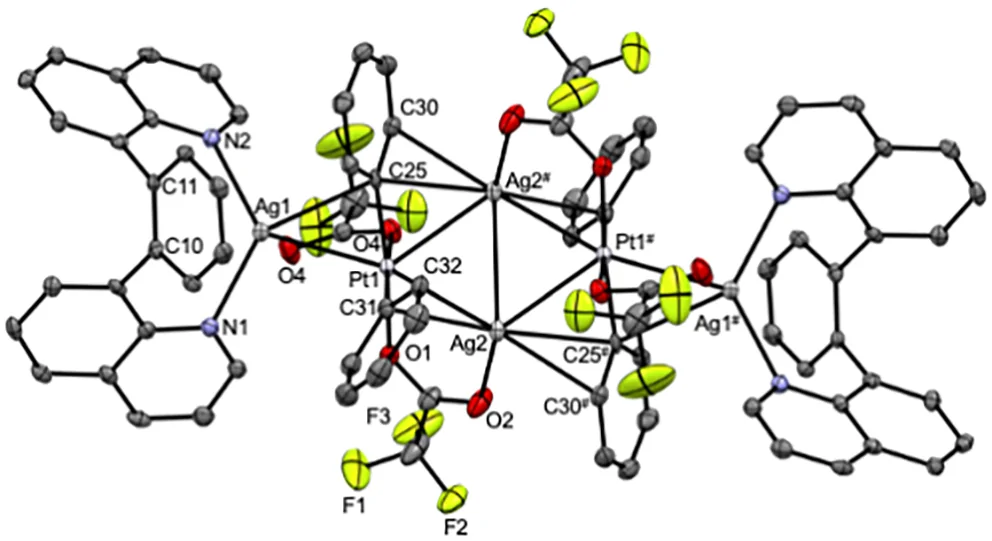
ORTEP of [(6-Fp)­AgPtPh_2_(TFA)­(μ-TFA)­Ag]_2_ (**2**). Ellipsoids are drawn at the 50% probability level. Hydrogen atoms, noncoordinating solvents, and the minor position of the disordered atoms are omitted for clarity.

**Table 2:** Selected Bond Lengths and Bond Angles for [(6-Fp)­AgPtPh_2_(TFA)­(μ-TFA)­Ag]_2_ (**2**)

bond distances	**2** (Å)
Ag1–N1 (6-Fp)	2.297(3)
Ag1–N2 (6-Fp)	2.337(3)
Ag1–C25(Ph)	2.699(4)
Ag1–C10(arene)	2.806(4)
Ag1–C11(arene)	3.048(4)
Ag1–Pt1	2.7884(3)
Pt1–Ag2	2.9449(3)
Pt1–Ag2^#1^	2.8248(3)
Pt1–C25 (Ph)	2.000(4)
Pt1–C31 (Ph)	1.994(4)
Pt1–O1	2.148(14)
Pt1–O3	2.121(3)
Ag2–Ag2^#1^	2.9905(6)
Ag2–C25^#1^	2.699(4)
Ag2–C30^#1^	2.636(4)
Ag2–C31	2.449(4)
Ag2–C32	2.549(4)
Ag2–O2	2.318(13)

bond angles	[deg]
N1–Ag1–N2	118.42(11)
C25–Pt1–C31	93.74(17)
Ag1–Pt1–Ag2	146.084(11)
Ag1–Pt1–Ag2^#1^	126.401(11)
Ag2–Pt1–Ag2^#1^	62.396(11)

The treatment of complex **1** with AgOTf in DCM resulted in a color change from orange to greenish-yellow. From this mixture, the heterometallic complex {[(6-Fp)­PtPh_2_Ag­(OTf)­(H_2_O)]_2_(μ-Ag)}­OTf (**3**) was isolated in 75% yield. The XRD analysis revealed an ionic framework comprising a free triflate anion and a 2:3 Pt/Ag ratio. Notably, the architecture of **3** diverges significantly from that of the AgTFA-derived complex **2** as illustrated in Figure 3. For complex **3**, the Pt center retains its coordination to the 6-Fp ligand and phenyl groups, similar to complex **1**. However, the Pt center establishes an additional bond with Ag­(I) likely via the donation of electron density from the Pt dz^2^ orbital into the Lewis acidic Ag­(I) center. This d^8^–d^10^ dative interaction is consistent with previously reported Pt–Ag assemblies.^21,24^ The Pt–Ag bond distance for **3** (2.75586(12) Å) is shorter than that observed for **2** (2.7884(3) Å), suggesting a more robust Pt–>Ag electronic donation. This enhanced metal–metal bonding has a measurable influence on the Pt coordination sphere (Table 3): the Pt–C_Ph_ bonds of **3** (Pt1–C25 = 2.036(2) Å; Pt1–C31 = 2.013(2) Å) are elongated relative to those of complex **1** (Pt1–C25 = 1.985(3) Å Pt1–C31 = 2.001(3) Å).

**Figure 3:**
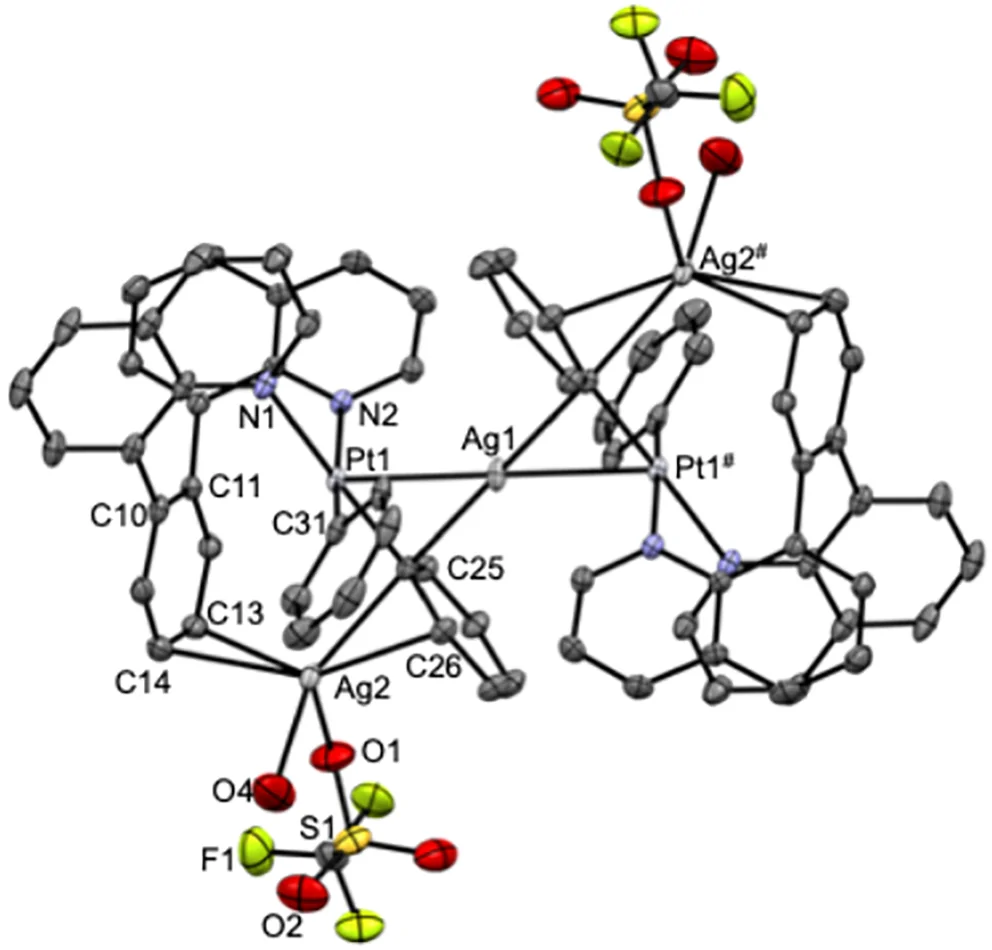
ORTEP of {[(6-Fp)­PtPh_2_Ag­(OTf)­(H_2_O)]_2_(μ-Ag)}­OTf, (**3**). Ellipsoids are drawn at the 50% probability level, and hydrogen atoms, noncoordinating solvents and OTf anion are omitted for clarity. O4 is connected with two hydrogen atom and it is a H_2_O molecule.

**Table 3:** Selected Bond Lengths and Bond Angles for (6-Fp)­PtPh_2_ (**1**), {[(6-Fp)­PtPh_2_Ag­(OTf)­(H_2_O)]_2_(*μ*-Ag)}­OTf (**3**) and {[(6-Fp)­PtPh_2_]_2_(*μ*-Ag)}­NO_3_ (**4**)

bond	**1** (Å)	**3** (Å)	**4** (Å)
Pt1–N1(6-Fp)	2.143(3)	2.1348(19)	2.134(12)
Pt1–N2(6-Fp)	2.162(3)	2.1828(19)	2.097(12)
Pt1–C25(Ph)	1.985(3)	2.036(2)	2.086(10)
Pt1–C31(Ph)	2.001(3)	2.013(2)	2.071(14)
Pt1–C10	2.998(4)	3.044(2)	2.927(10)
Pt1–C15	3.052(4)		
Pt1–C11		2.943(2)	2.949(10)
Pt1–Ag1		2.75586(12)	2.6812(2)
Ag1–C25(Ph)		2.577(2)	2.521 (17)
Ag1–C31(Ph)		3.063(3)	3.030(14)

bond angle	[deg]	[deg]	[deg]
N1–Pt1–N2	87.54(9)	83.94(7)	86.5(5)
C25–Pt1–C31	90.58(12)	92.20(9)	90.5(6)
Pt1–Ag1–Pt^#1^		180.0(0)	180.0(0)

Furthermore, the Ph–Pt–Ph angle widens to 92.20(9)° for **3** compared to 90.58(12)° for **1**. Interestingly, this angle is narrower than that observed in complex **2** (93.74(17)°) reflecting a specific accommodation of the steric and electronic demands at the Pt center. Another feature of **3** is the coordination environment of the peripheral Ag centers, which exhibit η^2^-interactions with both the arene moiety of the 6-Fp ligand and the phenyl groups, complemented by additional coordination of H_2_O and OTf ligands.

The addition of AgNO_3_ to complex **1** in DCM yielded the heterometallic complex {[(6-Fp)­PtPh_2_]_2_(μ-Ag)}­NO_3_ (**4**). Similar to **3**, the XRD analysis of a single-crystal of **4** reveals an ionic framework consisting of a 2:1 (Pt/Ag) complex cation and a noncoordinating nitrate anion (Figure 4). The Pt–Ag bond for **4** (2.6812(2) Å) is shorter than those observed for both **2** (2.7884(3) Å) and **3** (2.75586(12) Å). This Pt–Ag bond distance contraction could be due to the absence of any additional coordinated Ag in **4**, compared to **3**. This metal–metal interaction induces an influence as evidenced by the Pt–C_Ph_ bond lengths for **4** (Pt1–C25 = 2.086(14) Å; Pt1–C31 = 2.071(14) Å), which are elongated compared to complexes **1**, **2** and **3**. Furthermore, the Ag–C_Ph_ interaction for **4** (2.534(15) Å) is elongated relative to **2** and **3** (Table 3), indicating that the Ag­(I) center is likely more strongly sequestered by the Pt units for **4**.

**Figure 4:**
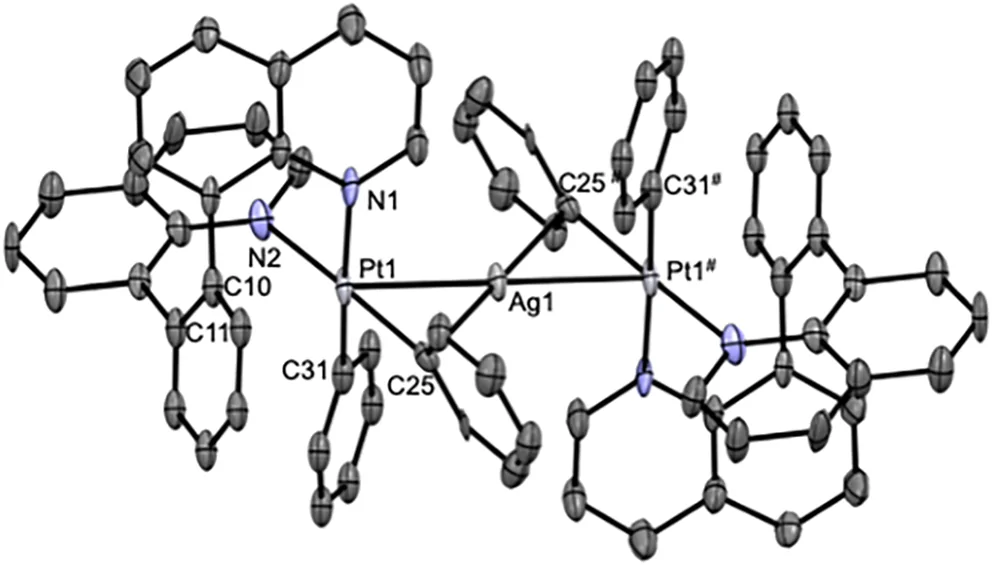
ORTEP of {[(6-Fp)­PtPh_2_]_2_(μ-Ag)}­NO_3_, (**4**). Ellipsoids are drawn at the 50% probability level. Hydrogen atoms, NO_3_ anions, and the minor position of the disordered atoms are omitted for clarity.

To establish that the solid-state structures determined crystallographically are representative of the bulk materials, UV–vis spectra of crystalline samples, bulk-isolated solids, and in situ generated solutions were compared in DCM. For complex **2**, the spectra of all three samples were nearly identical, indicating the formation of a single solution-state species upon treatment of **1** with AgTFA (Figure 5). Analogous results were obtained for **3** and **4** derived from AgOTf and AgNO_3_, respectively (Figures S24 and 25). The UV–vis spectra further differentiate the three systems: complex **2** displays a prominent absorption band at 417 nm that is absent for complexes **3** and **4**, whereas the latter two exhibit closely similar spectral profiles. These observations support the formation of distinct structural motifs depending on the counteranion of the Ag­(I) salt.

**Figure 5:**
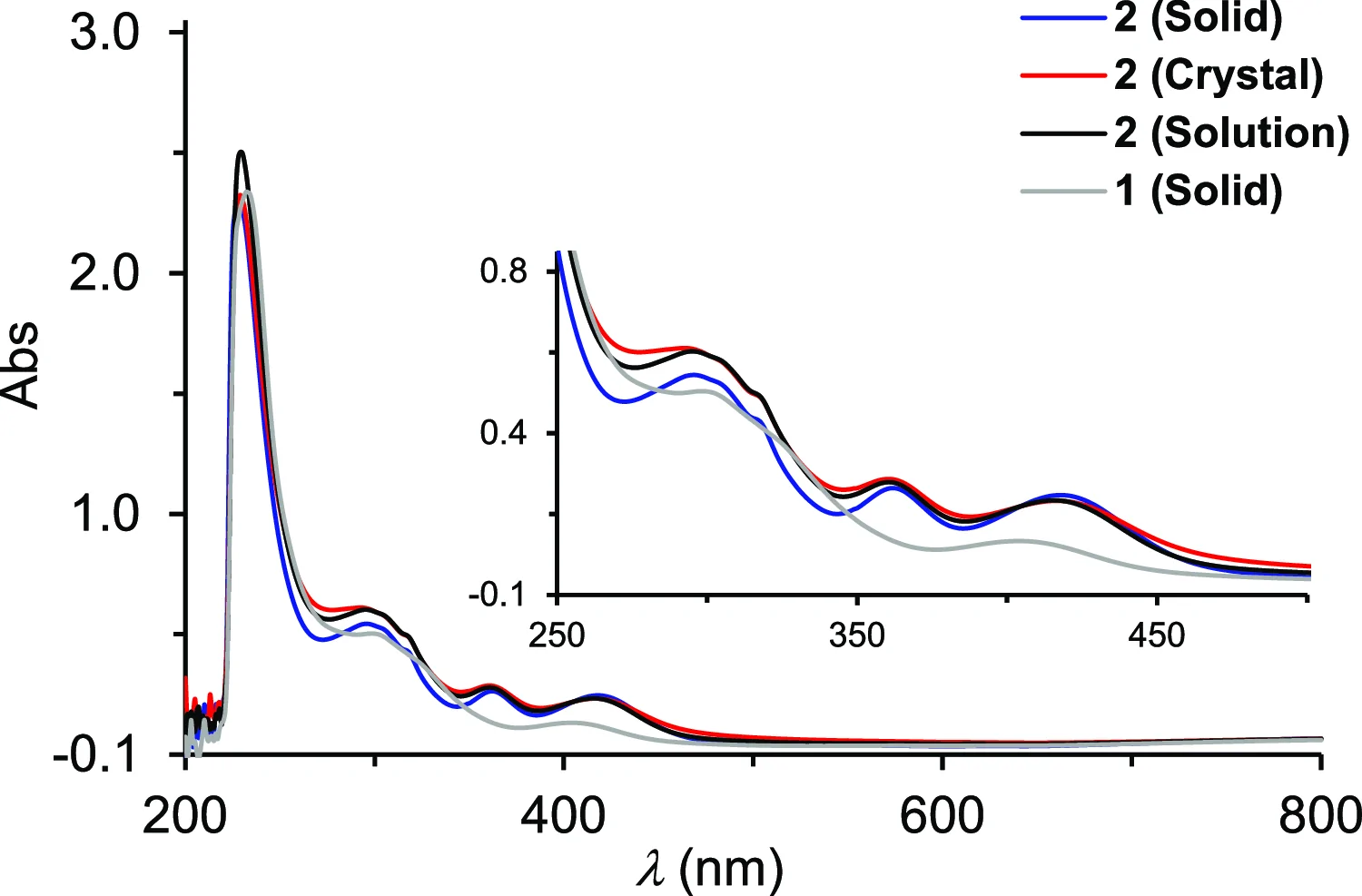
UV–vis spectra of complexes **1** and **2** in DCM solvent. Note: **2** (Solid) refers to the isolated solid obtained from the reaction of **1** with 2 equiv of AgTFA; **2** (Crystal) refers to a single crystal grown from the isolated solid; **2** (Solution) refers to the solution obtained after addition of 2 equiv of AgTFA to **1** and stirring for 30 min.

ICP–OES analysis confirmed the presence of both Pt and Ag in all heterometallic complexes. The experimentally determined Pt/Ag ratios were 1:2.8 for **2** (calcd 1:2), 1:2.05 for **3** (calcd 1:1.5), and 1:0.6 for **4** (calcd 1:0.5) (see Table S4). Although the measured values deviate slightly from the ideal stoichiometries, the overall trends are consistent with the proposed formulations.

### Reactivity Study of Complexes **1**–**4** at High Temperature

Treatment of **1** with various Ag­(I) salts resulted in the formation of biphenyl, and the corresponding time-course profiles are shown in Figure 6. The reactions with AgTFA and AgOTf proceeded at comparable rates, affording biphenyl in >85% yield within 2 h; prolonging the reaction time did not lead to any significant increase in yield. In contrast, the use of AgNO_3_ resulted in substantially slower biphenyl formation, with the yield remaining below 20% even after extended reaction times (see Figures S26–S28).

**Figure 6:**
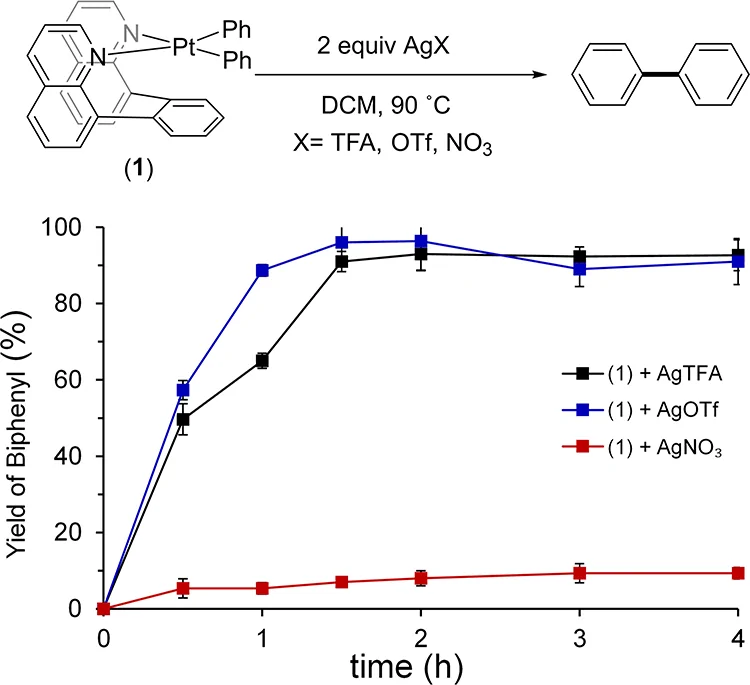
Kinetic plots for the formation of biphenyl from (6-Fp)­PtPh_2_ (**1**) with AgTFA (black), AgOTf (blue) or AgNO_3_ (red). Reaction conditions: 5 mM complex **1**, 10 mM Ag­(I) salt in J-Young NMR tube in CD_2_Cl_2_ solvent and heat the solution at 90 °C using a capillary of 1,3,5-trimethoxybenzene as internal standard. Each data point is the average of three independent experiments and error bars represent the standard deviation for the three experiments.

To probe the role of the proposed intermediates **2**, **3** and **4**, analogous time-course experiments were conducted using the independently synthesized complexes **2**–**4** under identical reaction conditions in DCM. As shown in Figure 7, (Figures S29–S31) these experiments revealed the same reactivity trend observed for complex **1** in combination with Ag­(I) salts. Collectively, these results support the assignment of complexes **2**–**4** as intermediates for the formation of biphenyl from **1**, indicating that biphenyl formation from the reaction of **1** and Ag­(I) salt likely proceeds through these Pt/Ag-heterometallic intermediates.

**Figure 7:**
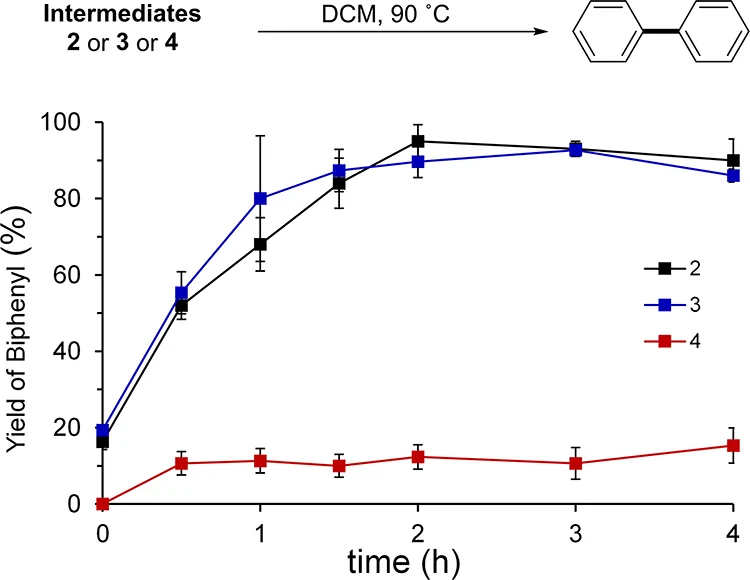
Kinetic plots for the formation of biphenyl from complexes **2** (black), **3** (blue) and **4** (red). Reaction conditions: 5 mg of the complexes dissolved in 0.5 mL CD_2_Cl_2_ in J-Young NMR tube using a capillary of 1,3,5-trimethoxybenzene as internal standard and heat the solution at 90 °C. Each data point is the average of three independent experiments and error bars represent the standard deviation for the three experiments.

Next, we sought to elucidate the fate of the Pt and Ag species at the end of the reaction following biphenyl formation. If the Ag­(I) entities are oxidizing Pt­(II) prior to biphenyl reductive elimination, the formation of Pt(s) might not be observed (e.g., oxidation to Pt­(IV) followed by reductive elimination would give a Pt­(II) product). Alternatively, if the role of Ag­(I) is to activate Pt­(II) toward biphenyl reductive elimination without prior oxidation of Pt­(II), the formation of Pt(s) is anticipated. Heating complex **1** with AgTFA, or independently prepared intermediate **2**, at 90 °C in DCM resulted in >90% biphenyl formation in both cases (Scheme 7). The reactions produced an off-white precipitate along with a brown-colored solution. ^1^H NMR analysis of the brown solution revealed the presence of a major 6-Fp-containing species accompanied by minor unidentified complexes/ligand. Single crystals suitable for XRD analysis were obtained from this solution, and structural analysis revealed the formation of a trimetallic silver complex ligated by a single 6-Fp ligand (complex **5**, Figure 8).

**Scheme 7:**
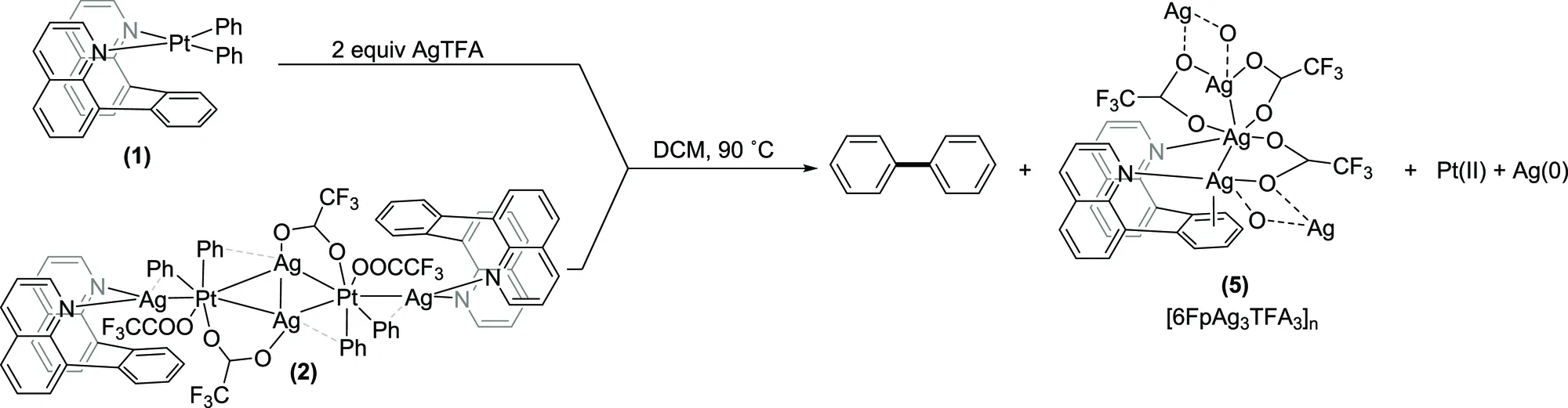
Reaction of **1** or **2** at Elevated Temperature and the End Products

**Figure 8:**
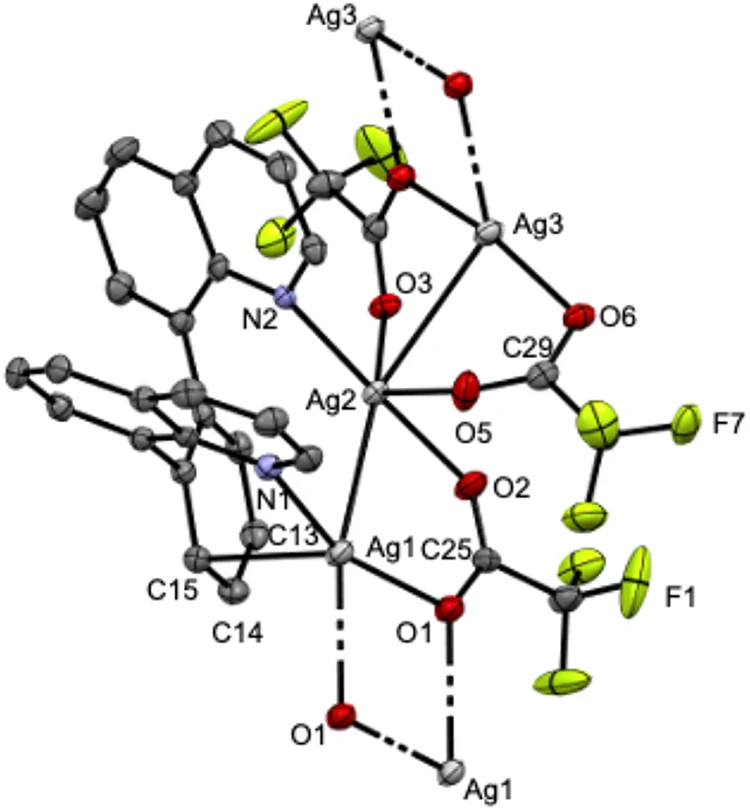
ORTEP of (6-Fp)­Ag_3_TFA_3_ (**5**). Ellipsoids are drawn at the 50% probability level, and hydrogen atoms are omitted for clarity. Selected distances [Å], and angles [deg]: Ag1–N1 2.225(3), Ag2–N2 2.263(3), Ag1–Ag2 2.9056(4), Ag2–Ag3 3.0658(4), Ag1–C15 2.619 (3), Ag1–C10 2.998(3), Ag2–C11 2.843(3), Ag1–O1 2.227(2), Ag2–O2 2.241(2), Ag2–O3 2.542(2), Ag2–O5 2.539(3), Ag3–O4 2.216(2), Ag3–O6 2.173(2) N1–Ag1–Ag2 82.89(7), N2–Ag2–Ag1 104.26(7), Ag1–Ag2–Ag3 139.701(12), Ag2–Ag1–O1 86.92(6), Ag1–Ag2–O2 75.89(6).

In the solid-state structure of **5**, two Ag­(I) centers are coordinated to the pyridyl nitrogen atoms of the 6-Fp ligand and are bridged to each other, while a third Ag­(I) ion is bound to this Ag–Ag unit. All three Ag centers are further coordinated by three bridging trifluoroacetate ligands, giving rise to a polymeric network composed of repeating (6-Fp)­Ag_3_(TFA)_3_ (**5**). The ^1^H NMR spectrum of the isolated crystalline material closely matches that of the major species observed in the brown reaction solution, indicating that this trimetallic silver complex is likely a dominant product in solutions after biphenyl formation (Figure S32).

We used X-ray photoelectron spectroscopy (XPS) in an effort to better understand the reactivity that leads to the formation of biphenyl. XPS method information is included in the Supporting Information. XPS analysis of the brown solution that is obtained after the reactions of complex **1** with AgTFA and AgOTf, respectively, confirmed the presence of Ag and Pt-species. The core level spectra are included in the Supporting Information (Figure S33). Pt 4f core level positions of complexes **2** and **3** are consistent with a Pt oxidation state most likely close to Pt­(II).^25^ XPS analysis of the off-white precipitate that is obtained from a reaction of **1** with AgTFA at 90 °C (labeled in Supporting Information as **2”**-**ppt**, see Scheme S2) does not reveal the presence of Pt. XPS analysis of the precipitate that forms from the reaction of **1** and AgOTf at 90 °C (**3”**-**ppt**) shows minimal Pt. These observations are consistent with the observation of a second ^195^Pt NMR signal in solution with AgTFA, indicating the Pt remains in solution after the reaction. XPS data reveal that both **2”**-**ppt** and **3”**-**ppt** contain Ag. The chemical shifts for Ag as a function of oxidation state are small (<0.5 eV) and higher oxidation states present with slightly lower binding energies contrary to most metals. From a comparison with the literature,^26^ and the spectra acquired in this work we suggest that the main composition of **2”**-**ppt** and **3”**-**ppt** is likely elemental Ag. The peak shape indicates the presence of more than one Ag species in **2”**-**ppt** with oxide, hydroxide or −N-Ag bonds the likely candidates as remnants of an incomplete reaction. XPS survey spectra do not show any halogens in the precipitates, and only small amounts of nitrogen, and as such is in agreement with our assignment of the Ag core level being dominated by elemental Ag.

Heating solutions of **1** with AgOTf, or independently prepared intermediate **3**, at 90 °C in DCM afforded biphenyl in >80% yield determined by ^1^H NMR spectroscopy. However, the ^1^H NMR spectrum of the crude reaction mixture at the end of the reaction was complicated, indicating the formation of multiple species (Figure S34). In contrast, treatment of **1** with AgNO_3_, or heating intermediate **4** under otherwise identical conditions, resulted in <15% biphenyl formation (Figure S35).

### Isotope Scrambling Experiment

To gain mechanistic insight into the biphenyl reductive elimination step, isotope scrambling experiments were conducted to assess whether phenyl exchange occurs prior to C–C bond formation. A 1:1 mixture of complexes **1** and **1**-***d*** **_10_** was stirred in DCM at room temperature for 4 days, and no significant formation of the crossover complex **1**-***d*** **_5_** was detected. In the presence of Ag­(I) salts and upon heating, the reaction produced biphenyl, biphenyl-*d*_10_, and the crossover product, biphenyl-*d*_5_. The appearance of biphenyl-*d*_5_ is consistent with phenyl group scrambling occurring before reductive elimination or that an intermolecular reaction mechanism in which two Pt centers react to give biphenyl is operative. The product ratios (biphenyl:biphenyl-*d*_5_:biphenyl-*d*_10_) differed for each Ag­(I) salt tested, as shown in Scheme 8, suggesting that the extent of isotopic mixing is sensitive to the reaction conditions. Because only partial scrambling was observed, the data do not exclude the possibility of multiple competing pathways, which complicates a definitive assignment of the mechanism.

**Scheme 8:**
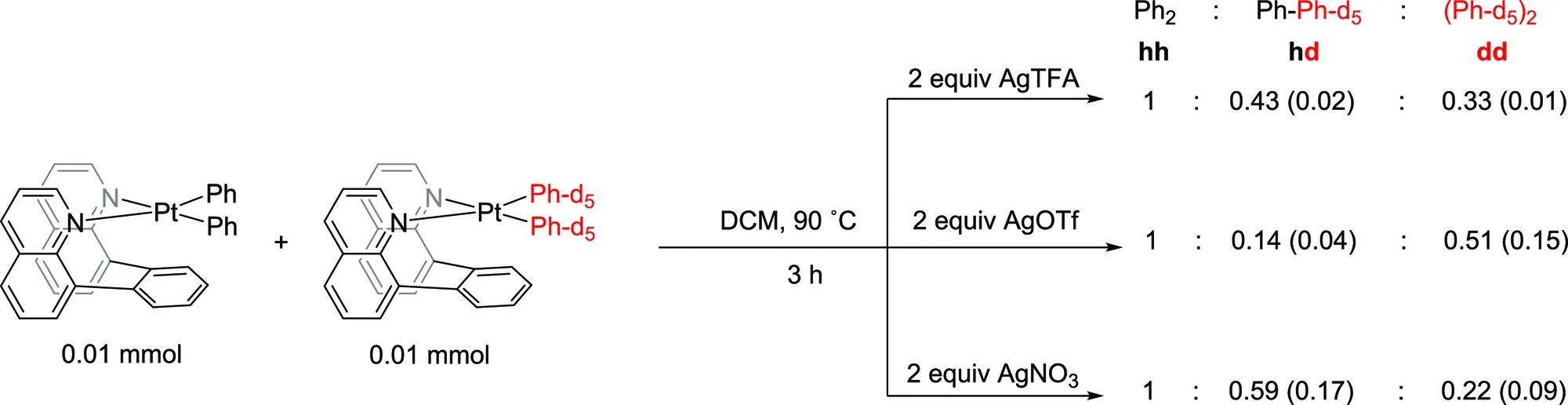
Isotope Scrambling Experiment

### Computational Evaluation of Structures and Reductive Elimination Barriers

Because multimetallic structures **2**, **3** and **4** have several metal–metal interactions and differing metal coordination environments, we first examined if the X-ray structures are the lowest energy configurations. To do this, we used the GOAT-explore program combined with g-xTB^27^ in ORCA^28^ and manual changes to generate new conformations and configurations for **2**, **3** and **4**. This resulted in the generation of thousands of alternative conformations and bonding configurations. Perhaps surprisingly, almost all alternative conformations and configurations were >10 kcal/mol higher in energy, except for one alternate configuration for **3**, which was 1 kcal/mol lower than the DFT optimized X-ray structure. However, this alternative structure does not provide a direct route for biphenyl formation. This analysis suggests that the formation of these specific multimetallic structures with the specific Ag and counterion is determined by thermodynamic stability. Molecular orbital analysis of structure **2** suggests that it forms through a combination of some Pt–Ag orbital/covalent bonding, which is an indication of some Pt center oxidation, and Lewis acid type interactions between Ag and phenyl groups. However, we note that natural bond orbital and intrinsic bond orbital analysis did not show significant Pt–Ag covalent character.

With the X-ray structures generally being the most stable structures and with a relatively large energy penalty to adopt an alternative conformation or bonding configuration, we used these structures to locate the biphenyl forming reductive elimination transition structures with M06/def2-SVP^29^ and continuum solvent. Quasiharmonic corrections for enthalpy and vibrational entropy calculations were applied (at 298.15 K and 1 atm) with a cutoff frequency of 100 cm^–1^. Note that due to the very large and flexible size of these structures some optimized structures have very small residual imaginary frequencies that were inverted to be positive.

Scheme 9 shows the calculated ground and transition structures and gives the corresponding activation enthalpy (Δ*H* ^‡^) and Gibbs free energies (Δ*G* ^‡^). All three transition structures have the expected one-step three-centered elimination partial bonding pattern that breaks two Pt–C bonds with incipient formation of the biphenyl C–C bond. All transition structures also feature one significantly elongated Pt–N­(diquinoline) distance, which is an effective ligand dissociation event prior to enabling reductive elimination structure. Importantly, the relative reductive elimination transition barriers for multimetallic structures **2**–**4** are consistent with the experimentally observed reaction conditions and biphenyl yields, ranging from Δ*H* ^‡^ ≈ 16 kcal/mol for **3** to Δ*H* ^‡^ ≈ 37 for **4**. We initially thought that the relative enthalpy of activations are related to the partial diquinoline dissociation, but this was ruled out by computing this dissociation energy for the respective ground states, which are all within 2 kcal/mol. Comparisons of the ground state structures to the transition structures reveal that for structures **2** and **3** the Pt–Ph group that is eliminated has significant direct π interactions with adjacent Ag metal centers and these interactions are mostly maintained in the corresponding transition structure. Furthermore, the triflate in structure **3** coordinates to an additional Ag center. In contrast, for **4**, the ground state Ph-to-Ag interaction is severed in the transition structure, which leads to the much higher enthalpy of activation and provides an explanation for the experimentally observed much slower biphenyl elimination and lower biphenyl yields. This highlights the role of the Ag modulating structures and reactivity at the Pt centers.

**Scheme 9:**
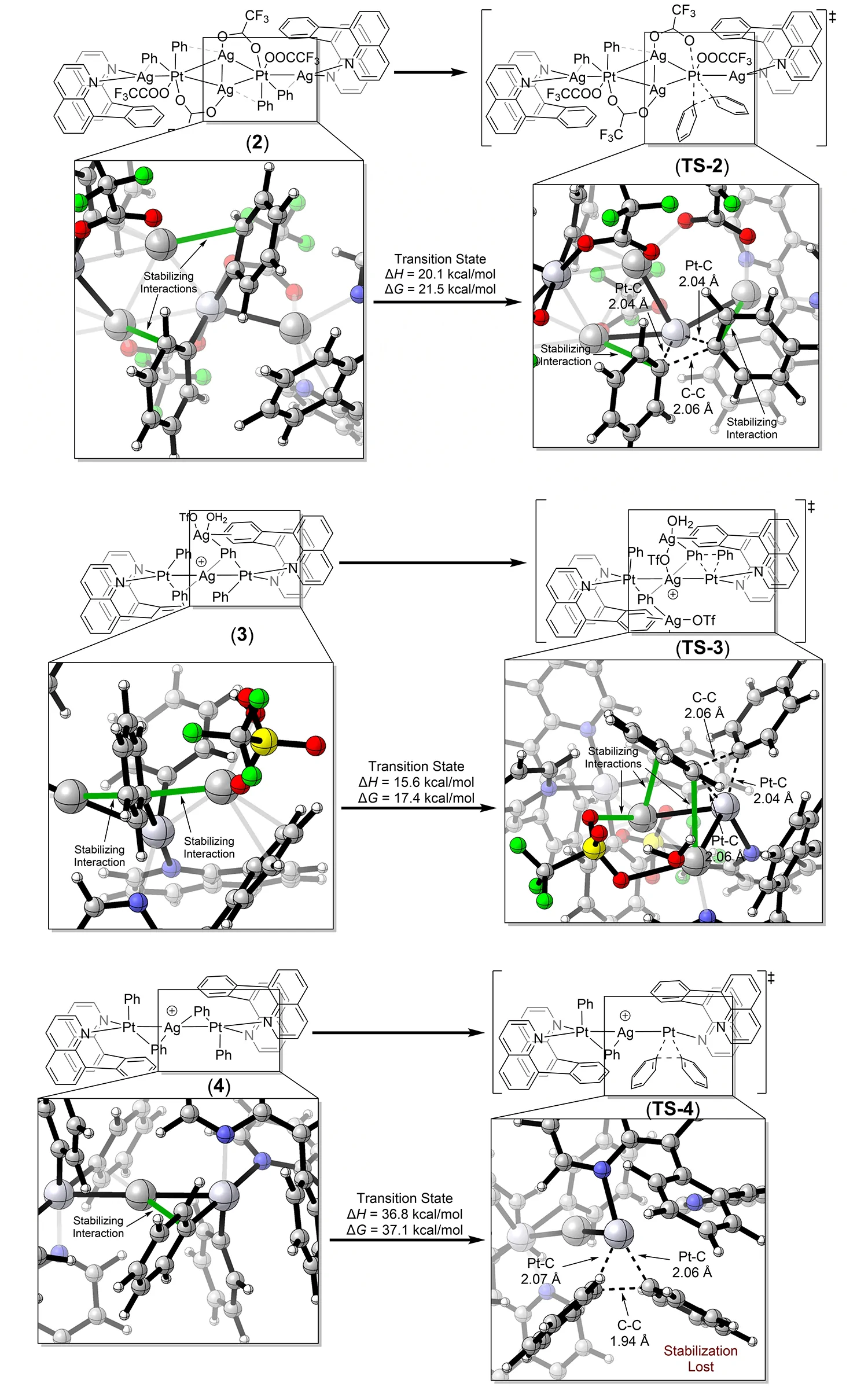
M06 Ground and Transition State Structures as Well as the Calculated Activation Enthalpy and Gibbs Energies

## Conclusions

The results described herein elucidate the role of Lewis acidic Ag­(I) additives in Pt-mediated C–C coupling reactions. In this study, we focused on understanding the influence of Ag­(I) salts in promoting reductive elimination from the (6-Fp)­PtPh_2_ (**1**) for biphenyl formation. Our findings demonstrate that treatment of **1** with Ag­(I) salts leads to the formation of heterometallic Pt/Ag intermediates featuring Pt–Ag interactions prior to C–C bond formation. The identity of the Ag counteranion was found to exert an influence on the rate and efficiency of biphenyl formation. While AgTFA and AgOTf displayed comparable reactivity, affording biphenyl in high yields, the use of AgNO_3_ exhibited markedly diminished activity. Structural studies revealed that the nature of the counteranion directs the formation of distinct reactive intermediates. Reaction of **1** with AgTFA resulted in metal transposition and formation of Pt–Ag species **2**. In contrast, treatment of **1** with AgOTf or AgNO_3_ produced trimetallic, bimolecular assemblies (**3** and **4**) in which two molecules of **1** are bridged by Ag­(I); in the case of **3**, additional AgOTf units engage in weak η^2^ or κ^1^-coordination to the arene moiety of the capping ligand and the phenyl group. Solution-state ^195^Pt NMR spectra support the formation of Pt/Ag bimetallic complexes elucidated by single-crystal X-ray diffraction. All three intermediates **2**–**4** undergo C–C reductive elimination to release biphenyl, albeit with different reactivity profiles: intermediates **2** and **3** led to near-quantitative biphenyl formation, whereas **4** displayed substantially lower activity. Computational studies further indicate that coordination of Ag­(I) induces structural and electronic perturbations at the Pt center that lower the barrier for biphenyl reductive elimination. Experimental isotopic scrambling experiments suggest that an intermolecular pathway could compete with the proposed intramolecular biphenyl reductive elimination. Overall, this work establishes that Ag­(I) additives play a complicated role in modulating C–C elimination that is more nuanced than simple electron transfer chemistry. These insights provide a broader framework for understanding the possible specific function(s) of Lewis acidic additives in transition-metal-catalyzed bond-forming reactions.

## Supplementary Material





## Notes

XYZ file for computational studies (XYZ)
Experimental details, additional screening results, control experiment, and compound characterization data with NMR spectra (PDF)
