# Supplementary material for: Ag(I)-Induced C–C Reductive Elimination from Pt(II): Role of Ag(I) and Counteranion
Source: Organometallics. 2026 Oct 15;45(20):2533–45. doi: 10.1021/acs.organomet.6c00318 (PMC13715644; doi:10.1021/acs.organomet.6c00318)
Supplement: Supplementary file 1 [file om6c00318_si_002.pdf]

Supporting Information for

Ag(I) Induced C–C Reductive Elimination from Pt(II): Role of Ag(I) and Counteranion

Md Asmaul Hoque[†], Zoë M. Gehman[†], Farzad Bastani[‡], Jonah G. Clark[Δ], Kevin P. Quirion[Δ], Aakashi Jain[†], Diane A. Dickie[†] Amrit Venkatesh[†,*], Petra Reinke[‡,*] Daniel H. Ess[Δ,*] and T. Brent Gunnoe[†,*]

[†]Department of Chemistry, University of Virginia, Charlottesville, Virginia, United States, 22904

[‡]Department of Materials Science and Engineering, University of Virginia; Charlottesville, Virginia 22904, United States

[Δ] Department of Chemistry and Biochemistry, Brigham Young University, Provo, Utah, United States, 84604

*Correspondence to: T. Brent Gunnoe: tbg7h@virginia.edu; Amrit Venkatesh: amrit.venkatesh@virginia.edu; Daniel H. Ess: dhe@byu.edu; Petra Reinke: pr6e@virginia.edu.

Table of contents

Contents

1. **General considerations** ... S2
2. **Synthesis of Pt-Complexes** ... S2
   Synthesis of (6-Fp)PtPh$_2$ (1). ... S4
   Synthesis of [(6-Fp)AgPtPh$_2$(TFA)(μ-TFA)Ag]$_2$ (2). ... S4
   Synthesis of {[(6-Fp)PtPh$_2$Ag(OTf)(H$_2$O)]$_2$(μ-Ag)}OTf (3). ... S5
   Synthesis of {[(6-Fp)PtPh$_2$]$_2$(μ-Ag)}NO$_3$, (4). ... S6
3. **Experimental procedure for oxidatively induced reductive elimination** ... S9
4. **Reaction of (6-Fp)PtPh$_2$ (1) with Ag salts at room temperature** ... S12
   4.1 Solution state characterization for the complexes ... S12
   4.2 ^{195}Pt NMR analysis for the complexes ... S18
   4.3 Solid state characterization (single crystal X-ray diffraction) ... S27
5. **UV-Vis spectroscopy** ... S30
6. **ICP-OES measurement** ... S31
7. **Reaction of (6-Fp)PtPh$_2$ (1) with Ag(I) salts at high temperature** ... S32
8. **XPS Study** ... S36
9. **Isotope scrambling experiment** ... S41
10. **Computational Study** ... S43
11. **References** ... S45

1. General considerations

Chemicals and solvents were purchased from commercially available sources and used without any purification. K_2PtCl_4, AgTFA, AgOTf, $AgNO_3$, quinolin-8-ylboronic acid, 1,2-diiodobenzene, Cs_2CO_3, $Pd(PPh_3)_4$, PhLi (1.6 M in hexane) were purchased from commercial sources including Combi-Blocks, TCI America, Ambeed, Stream, Sigma-Aldrich and VWR. The 6-Fp ligand was synthesized based on a reported procedure.[1] For other spectroscopic and electrochemical studies, HPLC-grade dichloromethane solvent was used.

NMR spectra (^1H, ^{13}C, and ^{19}F) were obtained using either a Bruker Avance NEO 400 MHz, 600 MHz or Avance III 800 MHz spectrometer with reference to residual solvent peaks: CD_2Cl_2 peaks at 5.32 ppm (^1H) and 53.84 ppm (^{13}C). Multiplicities are described using the following abbreviations: s = singlet, bs = broad singlet, d = doublet, dd = doublet of doublet, ddd = doublet of doublet of doublet, dt = doublet of triplet, ddt = doublet of doublet of triplet, t = triplet, td = triplet of doublet, q = quartet, m = multiplet. High-resolution mass spectrometry data were obtained using an Agilent 6545B Q-TOF mass spectrometer equipped with an electrospray ionization source, operating in positive and negative ion modes, at the University of Virginia. Voltammetric experiments were performed using an IJCambria CHI-700D potentiostat with a three-electrode one-compartment cell for Differential Pulse Voltammetry (DPV).

2. Synthesis of Pt-Complexes

Synthesis of $Cl_2Pt(SEt_2)_2$.

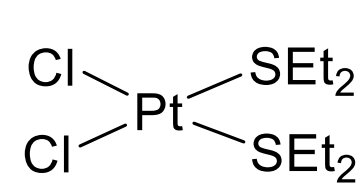

$Cl_2Pt(SEt)_2$

The complex $Cl_2Pt(SEt_2)_2$ was prepared according to a literature procedure.[2] In a 100 mL two-neck round-bottom flask, K_2PtCl_4 (2.00 g, 4.80 mmol) was dissolved in H_2O (80 mL). Diethyl sulfide (SEt_2, 2.0 mL, 18 mmol) was added with vigorous stirring, resulting in the formation of a pink suspension. The reaction mixture was heated at 80 °C overnight,

during which time the color changed to yellow, indicating the formation of $Cl_2Pt(SEt_2)_2$. The mixture was allowed to cool to room temperature and extracted with DCM (3 × 100 mL) until the aqueous layer became colorless. The combined organic extracts were dried over anhydrous $MgSO_4$, filtered, and concentrated under reduced pressure to afford $Cl_2Pt(SEt_2)_2$ as a yellow solid (1.70 g, 3.93 mmol, 82% yield).

Synthesis of [$Ph_2Pt(\mu\text{-}SEt_2)]_2$. The synthesis of [$Ph_2Pt(\mu\text{-}SEt_2)]_2$ was carried in glovebox under inert atmosphere at lower temperature obtained by using external dry ice-acetone mixture by adopting the reaction condition from the literature.[3] A solution of $Cl_2Pt(SEt_2)_2$ (1.20 g, 3.0 mmol) in dry THF (30 mL) was cooled to low temperature (~ −78 °C) using an external dry ice-acetone bath and stirred for 30 min. To this solution, a 1.6 M solution of phenyllithium in hexanes (8.0 mL, 15 mmol, 5 equiv) was added dropwise with stirring. During the addition, an initially formed yellow suspension gradually dissolved to give a homogeneous brown solution. The reaction mixture was allowed to warm to room temperature and stir overnight. The reaction flask was then removed from the glovebox, and the solution was maintained under a dinitrogen atmosphere. The reaction was quenched by slow addition of water (10 mL) to consume any unreacted lithium salt. The volume of the reaction mixture was reduced to approx. 10 mL and additional 10 mL water was added and the entire solution was extracted with DCM (3 × 20 mL). The combined organic extracts were dried over anhydrous $MgSO_4$, filtered, and concentrated under reduced pressure to afford [$Ph_2Pt(\mu\text{-}SEt_2)]_2$ as a gray solid (1.10 g, 1.25 mmol, 83% yield).

Synthesis of (6-Fp)PtPh$_2$ (1).

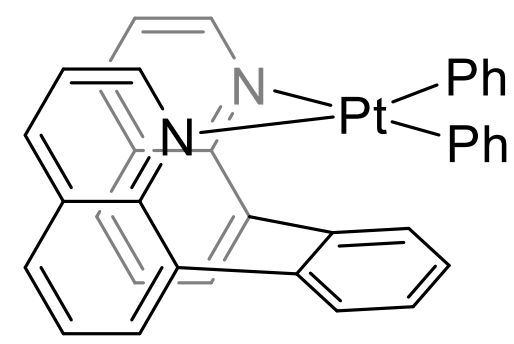

[(6-Fp)PtPh$_2$ **(1)**

A 500 mL two-neck round-bottom flask was charged with 6-Fp (450 mg, 1.40 mmol), $[Ph_2Pt(\mu\text{-}SEt_2)]_2$ (615 mg, 0.70 mmol) and diethyl ether (300 mL). The resulting suspended mixture was stirred at room temperature for 16 h, during which time it gradually transformed from a white suspension into a bright orange suspension solution. The solvent was removed under reduced pressure to afford an orange residue, which was dissolved in dichloromethane (5 mL). The resulting solution was treated with hexanes (200 mL), leading to the formation of an orange precipitate. The solid was collected by filtration, washed with hexanes (3 × 20 mL), and dried to yield (6-Fp)PtPh$_2$ (**1**) as an orange solid (660 mg, 72%). ^{1}H NMR (600 MHz, CD_2Cl_2) δ 9.26 (dd, $^3J_{HH}$ = 5.0, $^4J_{HH}$ =1.8 Hz, 1H), 8.12 (dd, $^3J_{HH}$ = 8.2, $^3J_{HH}$ = 1.8 Hz, 1H), 7.71 (dd, $^3J_{HH}$ = 5.6, $^4J_{HH}$ =3.4 Hz, 1H), 7.64 (dd, $^3J_{HH}$ = 8.0, $^4J_{HH}$ 1.6 Hz, 1H), 7.46 (dd, $^3J_{HH}$ = 7.1, $^4J_{HH}$ =1.6 Hz, 1H), 7.40 (dd, $^3J_{HH}$ = 5.6, $^4J_{HH}$ = 3.3 Hz, 1H), 7.34 (t, $^3J_{HH}$ = 7.6 Hz, 1H), 7.21 (dd, $^3J_{HH}$ = 8.2, $^3J_{HH}$ 4.9 Hz, 1H), 7.05 – 6.75 (m, 2H), 6.73 – 6.54 (m, 3H). ^{13}C NMR (201 MHz, CD_2Cl_2) δ 153.7, 146.9, 140.2, 140.1, 139.7, 139.2, 137.6, 133.7, 131.1, 130.2, 128.4, 128.1, 126.8, 125.7, 121.7, 121.1. HRMS (ESI) exact mass calculated for $[M+H]^+$, $[PtC_{36}H_{27}N_2]^+$: 682.1817, found m/z 682.1812.

Synthesis of [(6-Fp)AgPtPh$_2$(TFA)(μ-TFA)Ag]$_2$ (2).

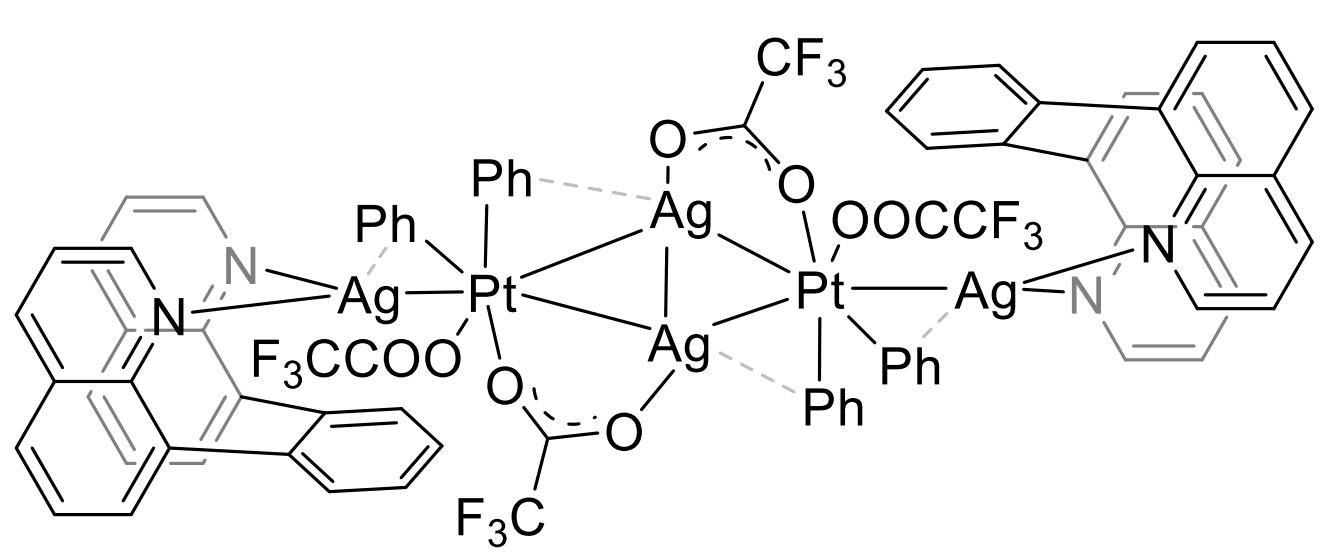

[(6-Fp)AgPtPh$_2$(TFA)(μ-TFA)Ag]$_2$ **(2)**

A 20 mL vial was charged with (6-Fp)$PtPh_2$ (**1**, 68 mg, 0.10 mmol) and dichloromethane (10 mL) to afford a clear orange solution. Silver trifluoroacetate (AgTFA; 44.2 mg, 0.20 mmol, 2.0 equiv) was added in one portion, and the reaction mixture was gently swirled at room temperature for 30 min. During this time, the solution color gradually deepened from orange to dark orange. The reaction mixture was then concentrated under reduced pressure to approximately 2 mL, and hexanes (10 mL) were added to induce precipitation. The resulting dark orange solid was collected by filtration, washed with hexanes (3 × 10 mL), and dried under vacuum afforded yield [(6-Fp)$AgPtPh_2$(TFA)(μ-TFA)$Ag]_2$ (**2**) as a dark orange solid (96 mg, 0.04 mmol, 86%). Note: The use of 2.0 equiv of AgTFA to complex **1** occasionally resulted in incomplete conversion under these conditions. Increasing the loading to 4.0 equiv led to complete conversion within 10–15 min. In both cases, the same product **2** was obtained, as confirmed by ^{1}H NMR spectroscopy and single-crystal X-ray diffraction. ^{1}H NMR (600 MHz, CD_2Cl_2) δ 8.63 (d, $^3J_{HH}$ = 3.8 Hz, 1H), 8.11 (dd, $^3J_{HH}$ = 8.3, $^4J_{HH}$ =1.8 Hz, 1H), 7.66 (dd, $^3J_{HH}$ = 8.2, $^4J_{HH}$ =1.5 Hz, 1H), 7.49 (dd, $^3J_{HH}$ = 7.1, $^4J_{HH}$ =1.5 Hz, 1H), 7.47 – 7.36 (m, 4H), 7.26 (dd, $^3J_{HH}$ = 8.3, $^3J_{HH}$ = 4.5 Hz, 1H), 7.19 (d, $^3J_{HH}$ = 7.4 Hz, 2H), 7.15 (t, $^3J_{HH}$ = 7.3 Hz, 1H), 7.08 (t, $^3J_{HH}$ = 7.5 Hz, 2H). ^{13}C NMR (201 MHz, CD_2Cl_2) δ 164.1 (q, J = 38.2 Hz), 162.6 (q, J = 38.2 Hz),) 153.0, 144.3, 138.7, 137.9, 136.6, 136.2, 133.4, 131.9, 129.4, 129.2, 129.0, 128.7, 126.5, 121.6. ^{19}F NMR (376 MHz, CD_2Cl_2) δ –73.5, –74.3. In the HRMS (ESI) analysis the molecular ion peak was not observed. A major fragmentation of $[6\text{-FpAg}]^+$ was observed. $[AgC_{24}H_{16}N_2]^+$: 439.0359, found m/z 439.0364.

Synthesis of {[(6-Fp)$PtPh_2$Ag(OTf)(H_2O)$]_2$(μ-Ag)}OTf (3).

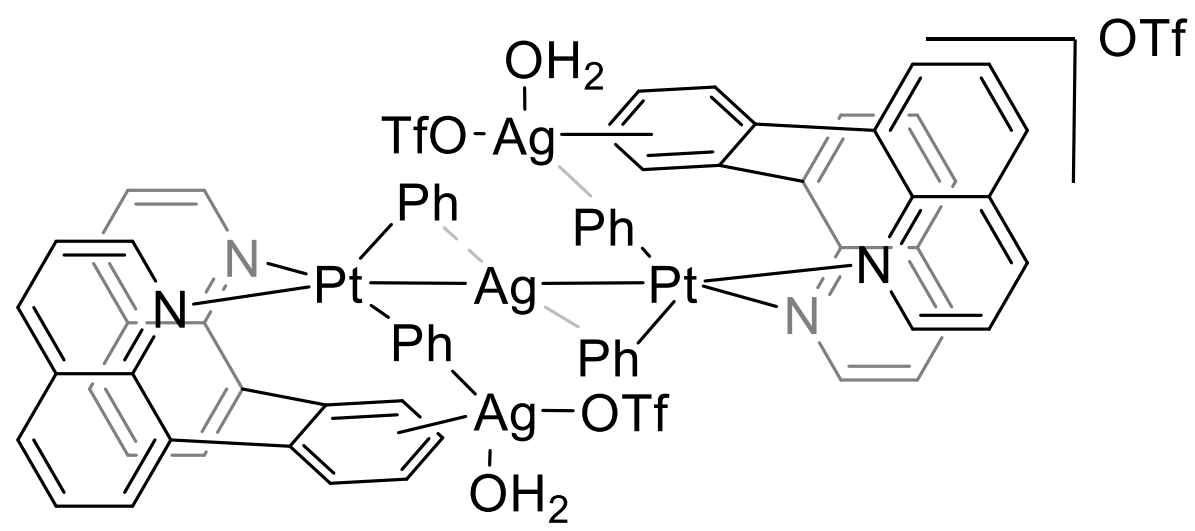

{[(6-Fp)$PtPh_2$Ag(OTf)(H_2O)$]_2$(μ-Ag)}OTf **(3)**

A 20 mL vial was charged with (6-Fp)PtPh$_2$ (**1**, 68 mg, 0.10 mmol) and dichloromethane (10 mL) to afford a clear orange solution. Silver triflate (AgOTf; 57.2 mg, 0.20 mmol, 2.0 equiv) was added in one portion, and the reaction mixture was gently swirled at room temperature for 30 min. During this time, the solution color gradually changed from orange to brown. The reaction mixture was then concentrated under reduced pressure to approximately 2 mL, and hexanes (10 mL) were added to induce precipitation. The resulting gray solid was collected by filtration, washed with hexanes (3 × 10 mL), and dried under vacuum afforded {[(6-Fp)PtPh$_2$Ag(OTf)(H$_2$O)]$_2$(μ-Ag)}OTf (3) as a gray solid (76 mg, 0.035 mmol, 70%). ^{1}H NMR (600 MHz, CD$_2$Cl$_2$) δ 9.23 (d, $^3J_{HH}$ = 5.1 Hz, 1H), 8.20 (d, $^3J_{HH}$ = 8.4 Hz, 1H), 7.69 (dd, $^3J_{HH}$ = 6.6, $^4J_{HH}$ = 2.9 Hz, 2H), 7.47 – 7.34 (m, 3H), 7.35 – 7.24 (m, 1H), 7.20 – 6.99 (m, 2H), 6.98 – 6.84 (m, 3H). ^{13}C NMR (201 MHz, CD$_2$Cl$_2$) δ 154.7, 146.0, 140.8, 139.7, 137.9, 137.8, 134.8, 132.4, 130.4, 129.2, 127.5, 127.2, 126.9, 124.5, 122.6, 119.3. ^{19}F NMR (377 MHz, CD$_2$Cl$_2$) δ –77.6. In the LCMS (ESI) analysis the molecular ion peak was not observed. A major fragmentation of [M-2Ag(OTf)(H$_2$O)]$^+$ was observed. Calculated for [$C_{72}H_{54}AgN_4Pt_2$]$^+$, 1471.2695 found m/z 1471.2572.

Synthesis of {[(6-Fp)PtPh$_2$]$_2$(μ-Ag)}NO$_3$, (4).

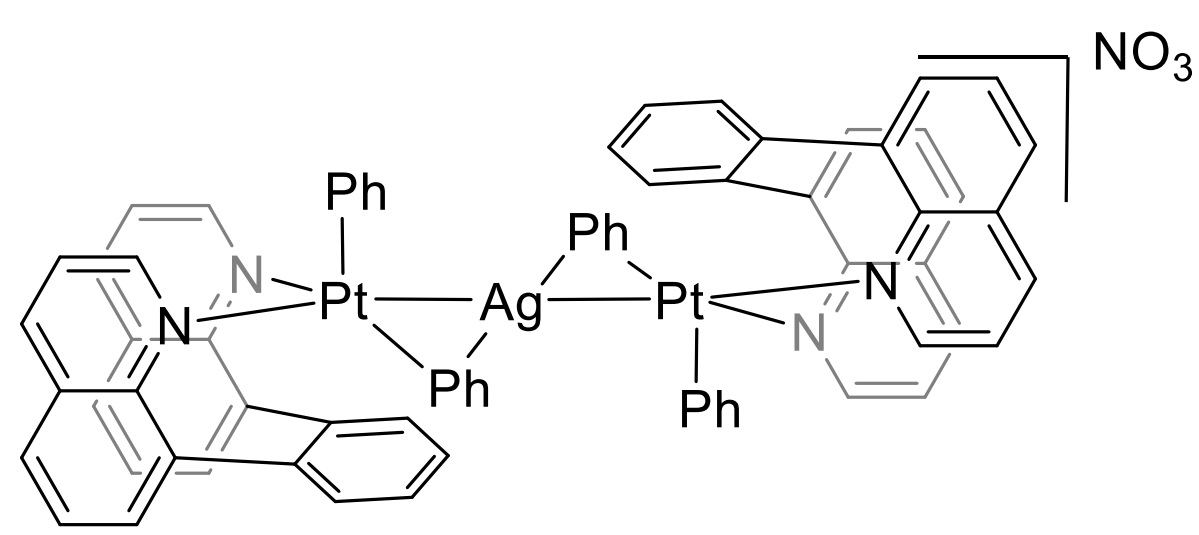

{[(6-Fp)PtPh$_2$]$_2$(μ-Ag)}NO$_3$ **(4)**

A 20 mL vial was charged with (6-Fp)PtPh$_2$ (**1**, 68 mg, 0.10 mmol) and dichloromethane (10 mL) to afford a clear orange solution. Silver Nitrate (AgOTf; 34 mg, 0.20 mmol, 2.0 equiv) was added in one portion, and the reaction mixture was gently swirled at room temperature for 30 min. During this time, the solution color gradually changed from orange to light orange. The reaction mixture was then concentrated under reduced pressure to approximately 2 mL, and hexanes (10 mL) were added to induce precipitation. The

resulting off-white solid was collected by filtration, washed with hexanes (3 × 10 mL), and dried under vacuum afforded {[(6-Fp)PtPh$_2$]$_2$(μ-Ag)}NO$_3$ (**4**) as an off-white solid (65 mg, 0.042 mmol, 85%). ^{1}H NMR (600 MHz, CD$_2$Cl$_2$) δ 8.50 (s, 1H), 8.16 (dd, $^3J_{HH}$ = 8.4, $^4J_{HH}$ = 1.7 Hz, 1H), 7.65 (dd, $^3J_{HH}$ = 7.6, $^4J_{HH}$ = 2.1 Hz, 1H), 7.48 (dd, $^3J_{HH}$ = 5.7, $^3J_{HH}$ = 3.3 Hz, 1H), 7.41 – 7.30 (m, 2H), 7.24 (dd, $^3J_{HH}$ = 5.6, $^4J_{HH}$ = 3.3 Hz, 1H), 7.00 (dd, $^3J_{HH}$ = 8.4, $^4J_{HH}$ = 5.0 Hz, 1H), 6.94 (d, $^3J_{HH}$ = 6.8 Hz, 1H), 6.88 – 6.66 (m, 4H). ^{13}C NMR (201 MHz, CD$_2$Cl$_2$) δ 153.3, 146.4, 139.6, 138.9, 138.8, 138.4, 134.1, 132.2, 129.9, 128.4, 128.3, 127.1, 126.5, 124.1, 121.6. HRMS (ESI) exact mass calculated for [M+2H]$^+$: [$C_{72}H_{54}AgN_4Pt_2$]$^+$, 1471.2695 found m/z 1471.2710.

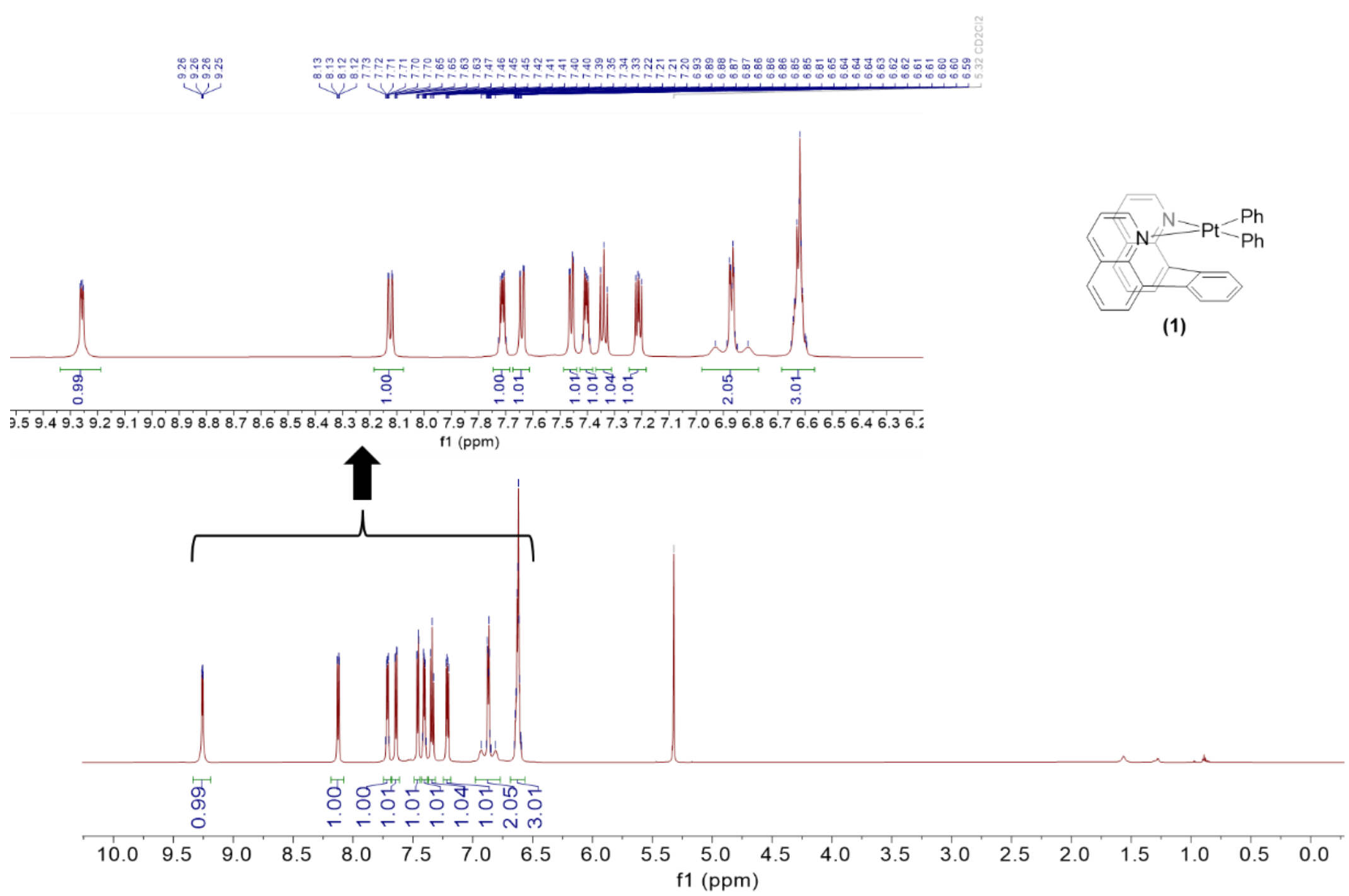

Figure S1. ^{1}H NMR spectrum of (6-Fp)PtPh$_2$ (**1**) in CD$_2$Cl$_2$.

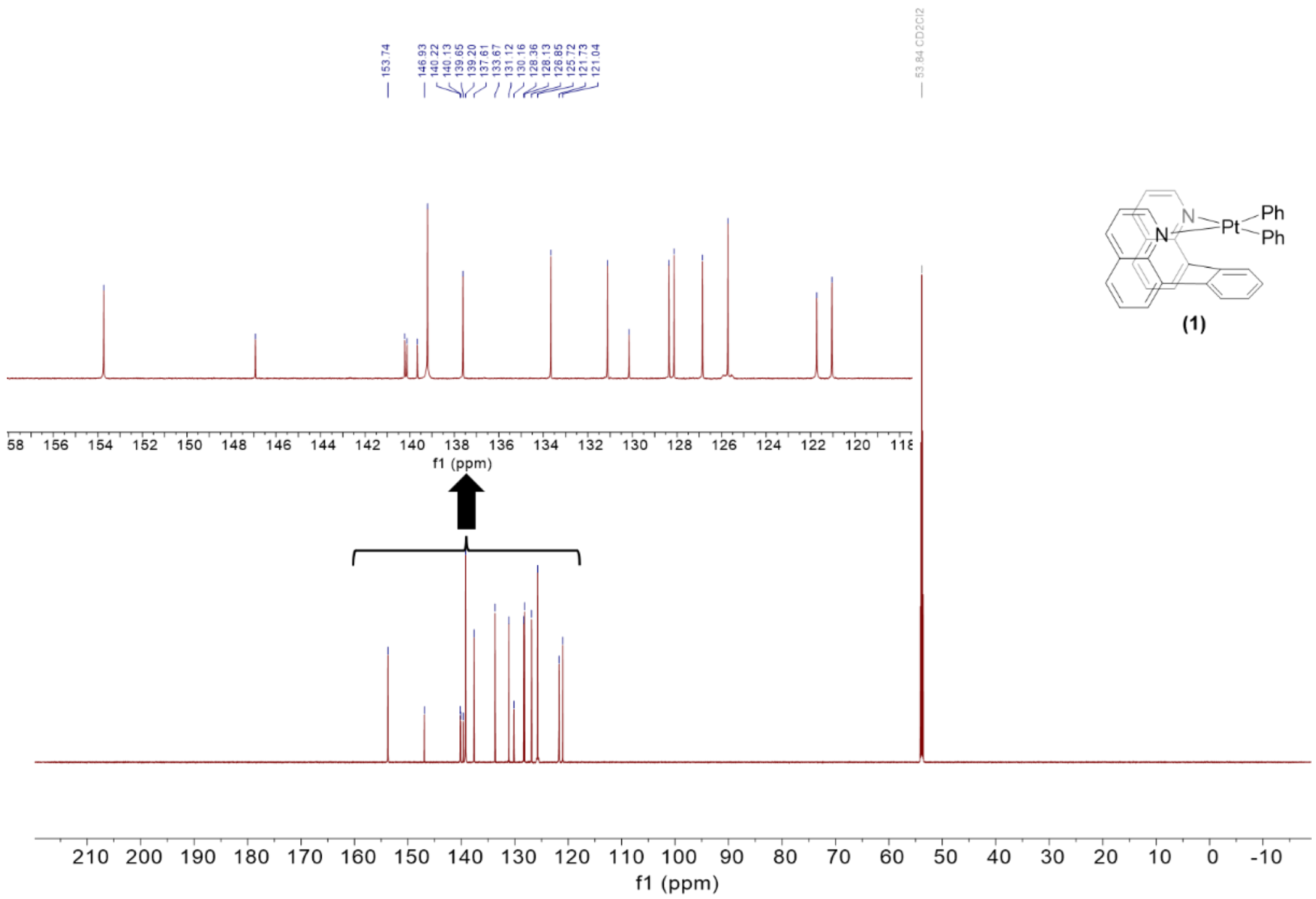

Figure S2. ^{13}C NMR spectrum of complex (6-Fp)PtPh$_2$ (**1**) in CD_2Cl_2.

3. Experimental procedure for oxidatively induced reductive elimination

Reactions were conducted in J-Young NMR tubes or standard NMR tubes sealed with the cap wrapped in electrical tape to minimize solvent loss during heating. Each tube was charged with a solution of (6-Fp)$PtPh_2$ (**1**) (0.5 mL, 5.0 mM in CD_2Cl_2) prepared from a stock solution. The appropriate Ag(I) reagent (1, 2 or 4 equiv) was then added directly to the NMR tube. A sealed capillary containing 1,3,5-trimethoxybenzene in CD_2Cl_2 was inserted into each tube as an internal standard and ^{1}H NMR spectra was acquired to integrate against standard before the reaction started. The NMR tube was placed in a preheated sand bath maintained at 90 °C. Reaction progress and yields were determined by ^{1}H NMR spectroscopy using the internal standard. For time-course studies, the NMR tube was removed from the sand bath and cooled to room temperature prior to data acquisition. After each ^{1}H NMR measurement, the sample was reheated under identical conditions to continue the reaction and obtain subsequent time points

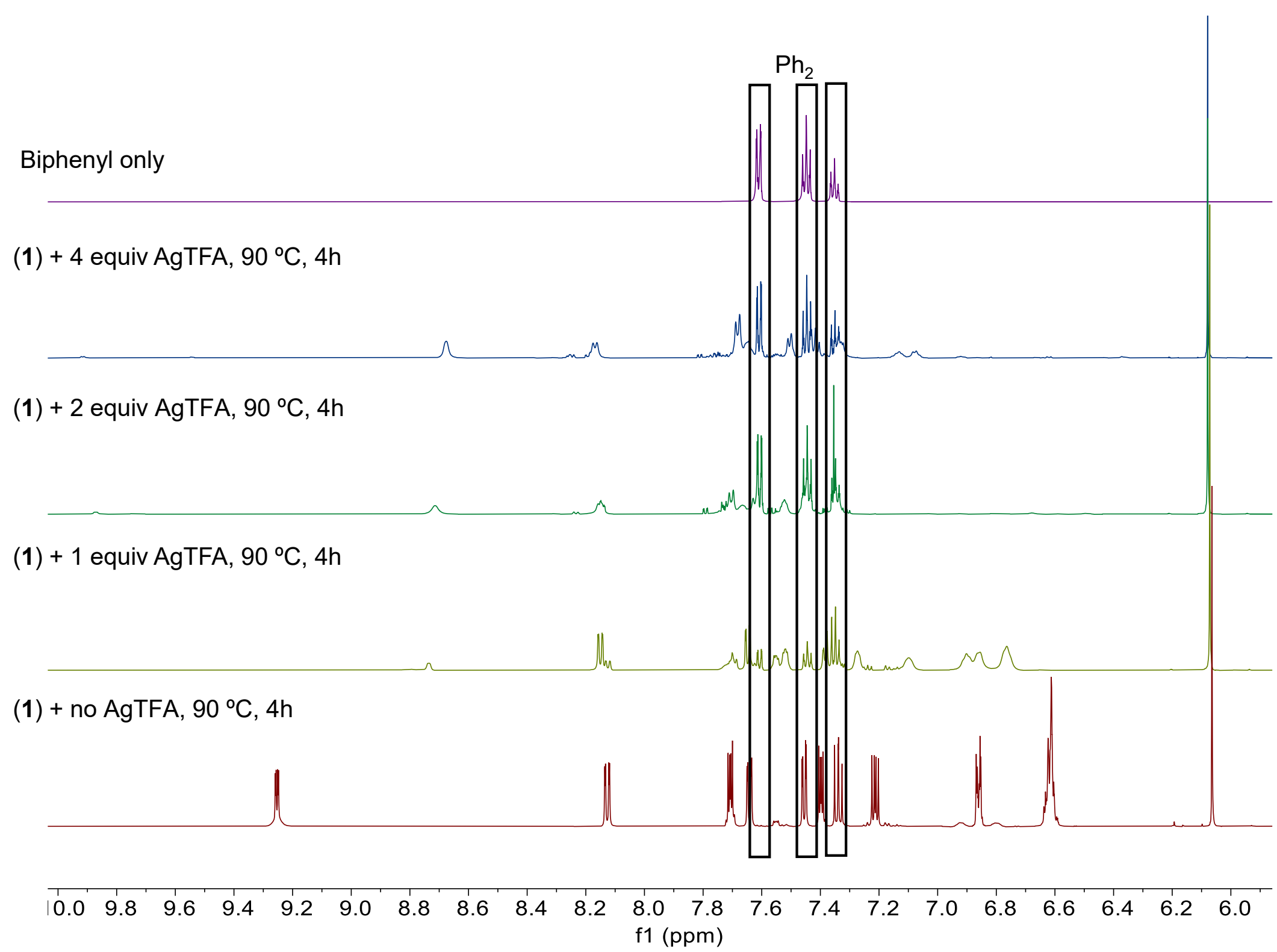

Figure S3. ^{1}H NMR spectra for the reaction of (6-Fp)$PtPh_2$ (**1**) with 1, 2 and 4 equiv of AgTFA in CD_2Cl_2 at 90 ºC.

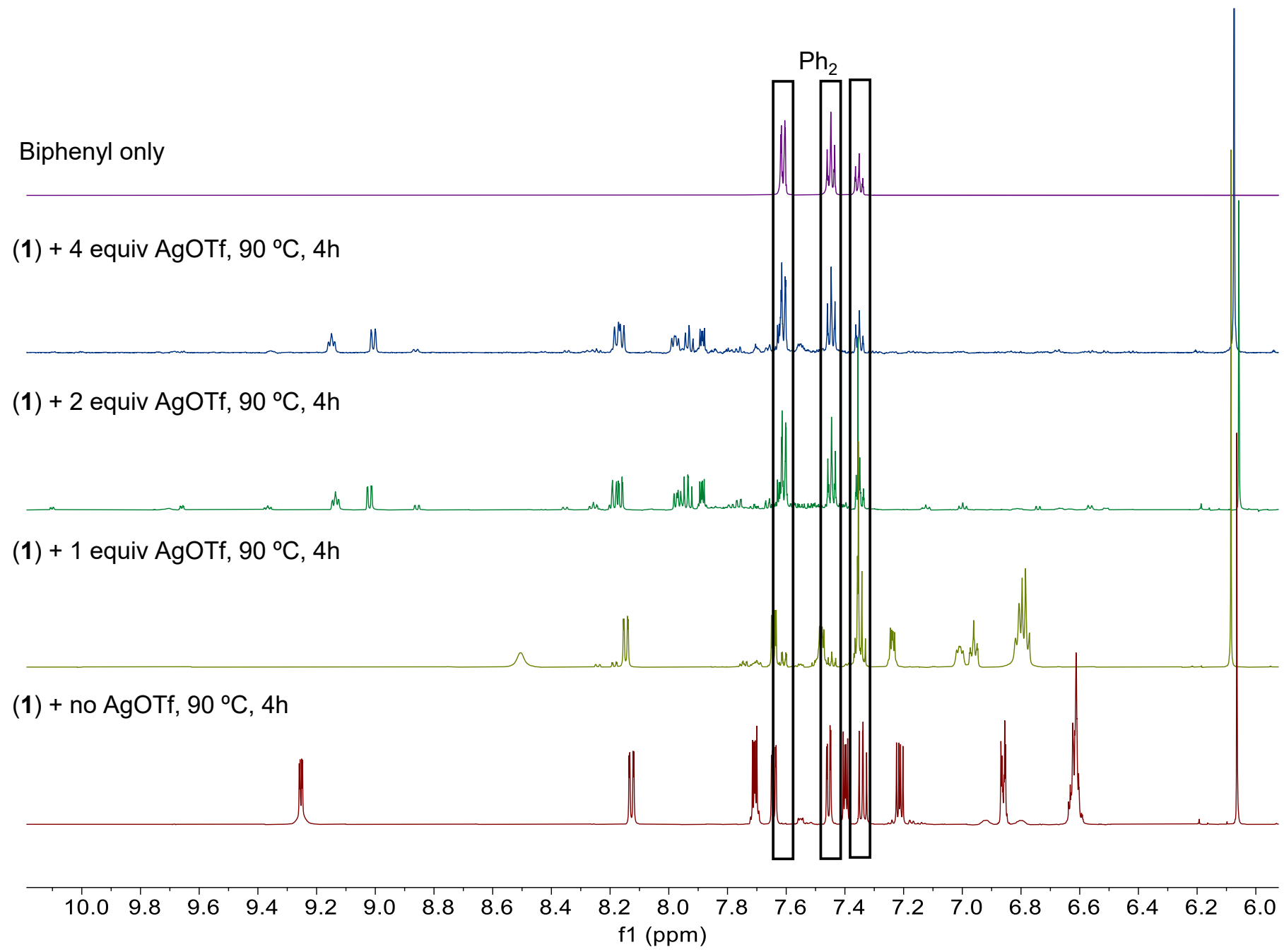

Figure S4. ^{1}H NMR spectra for the reaction of (6-Fp)PtPh$_2$ (**1**) with 1, 2 and 4 equiv of AgOTf in CD_2Cl_2 at 90 ºC.

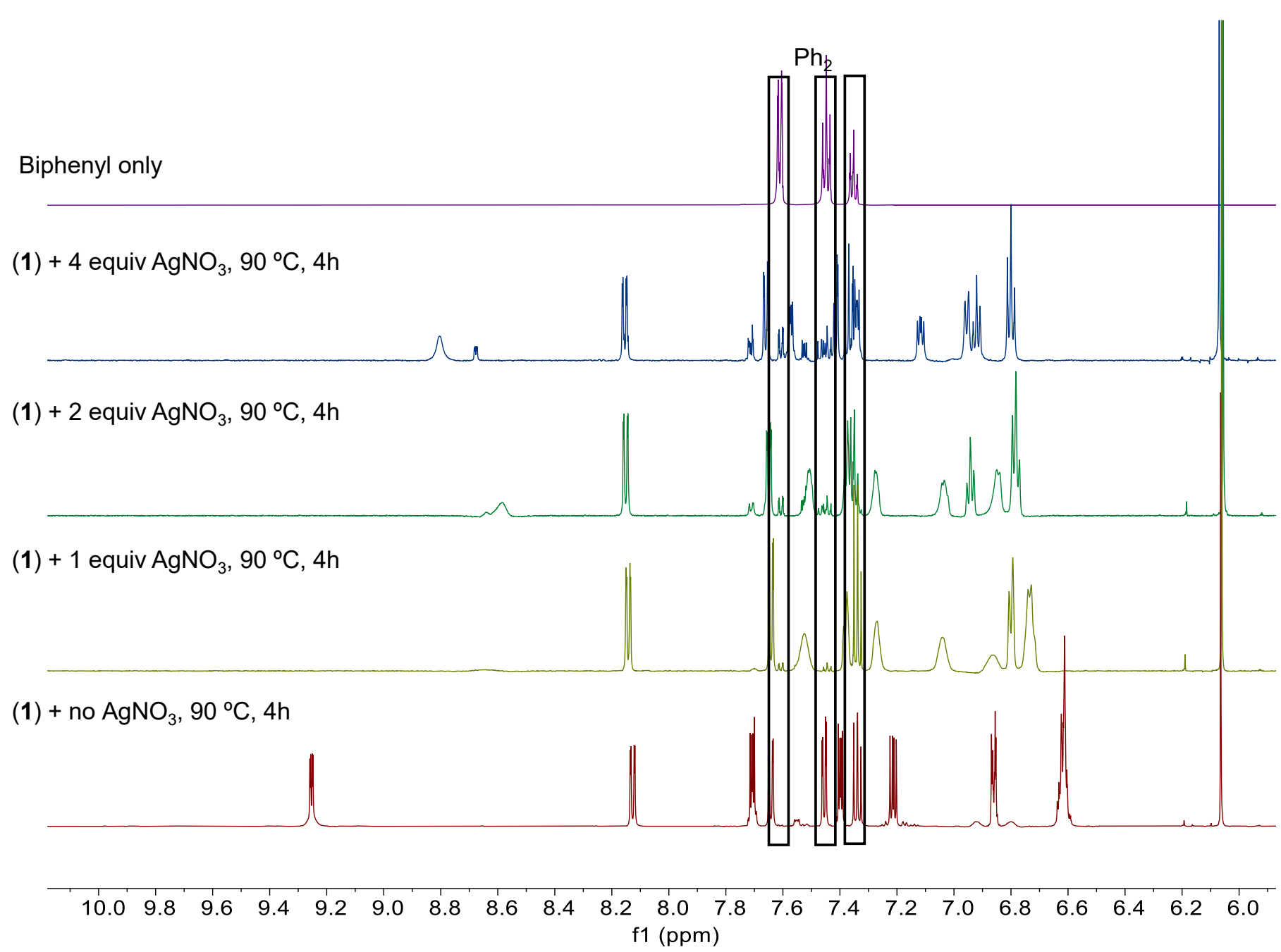

Figure S5. ^{1}H NMR spectra for the reaction of (6-Fp)PtPh$_2$ (**1**) with 1, 2 and 4 equiv of $AgNO_3$ in CD_2Cl_2 at 90 ºC.

Redox Potential measurement. The spectral change of (6-Fp)PtPh2 (**1**) with various Ag(I) salts leads to the formation of different complexes in room temperature as well as at 90 ºC. In order to understand the different reactivity of Ag(I) with various counteranion such as trifluoroacetate, triflate and nitrate, the redox potential of Ag(I) salts was measured using differential pulse voltammetry (DPV) as well as cyclic voltammetry (CV).

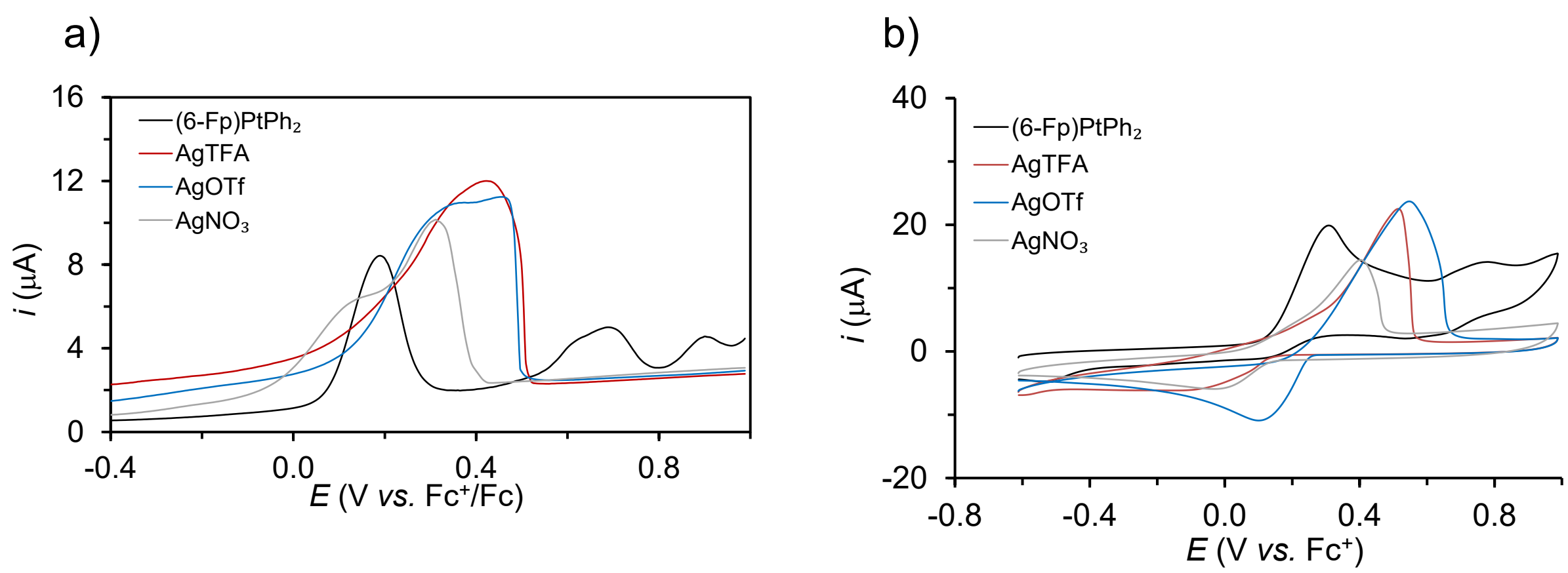

Figure S6. a) Differential pulse voltammetry (DPV) and b) Cyclic Voltammetry (CV) of complex (6-Fp)PtPh2 (**1**, Black) and silver salts, AgTFA (red), AgOTf (blue) and $AgNO_3$ (gray) in DCM solvent using 0.1 M $TBAPF_6$ as an electrolyte, Glassy carbon as working electrode, Pt as counter electrode and Ag/Ag^+ as reference electrode. Potential was adjusted in reference to Fc/Fc^+. The DPV parameters were ΔE = 4 mV, amplitude = 5 mV, pulse width = 0.05 s, sampling width = 0.0167 s, and pulse period = 0.5 s. For CV, scan rate is 50 mV/s. Scan direction initiated from low potential.

Note: Because AgTFA, AgOTf, and $AgNO_3$ have limited solubility in DCM, the CV and DPV measurements were conducted under partially heterogeneous conditions. The non-ideal voltammetric profiles suggest possible Ag electrodeposition at the electrode surface. Nevertheless, the distinct redox potentials observed with the three Ag(I) salts indicate that the counteranion influences the electrochemical behavior of Silver salt.

4. Reaction of (6-Fp)$PtPh_2$ (1) with Ag salts at room temperature

The reaction of (6-Fp)$PtPh_2$ (1) with AgTFA, AgOTf and $AgNO_3$ at room temperature leads to the formation of new complexes which we define as an intermediate. Upon heating those intermediate leads to the formation of biphenyl.

4.1 Solution state characterization for the complexes

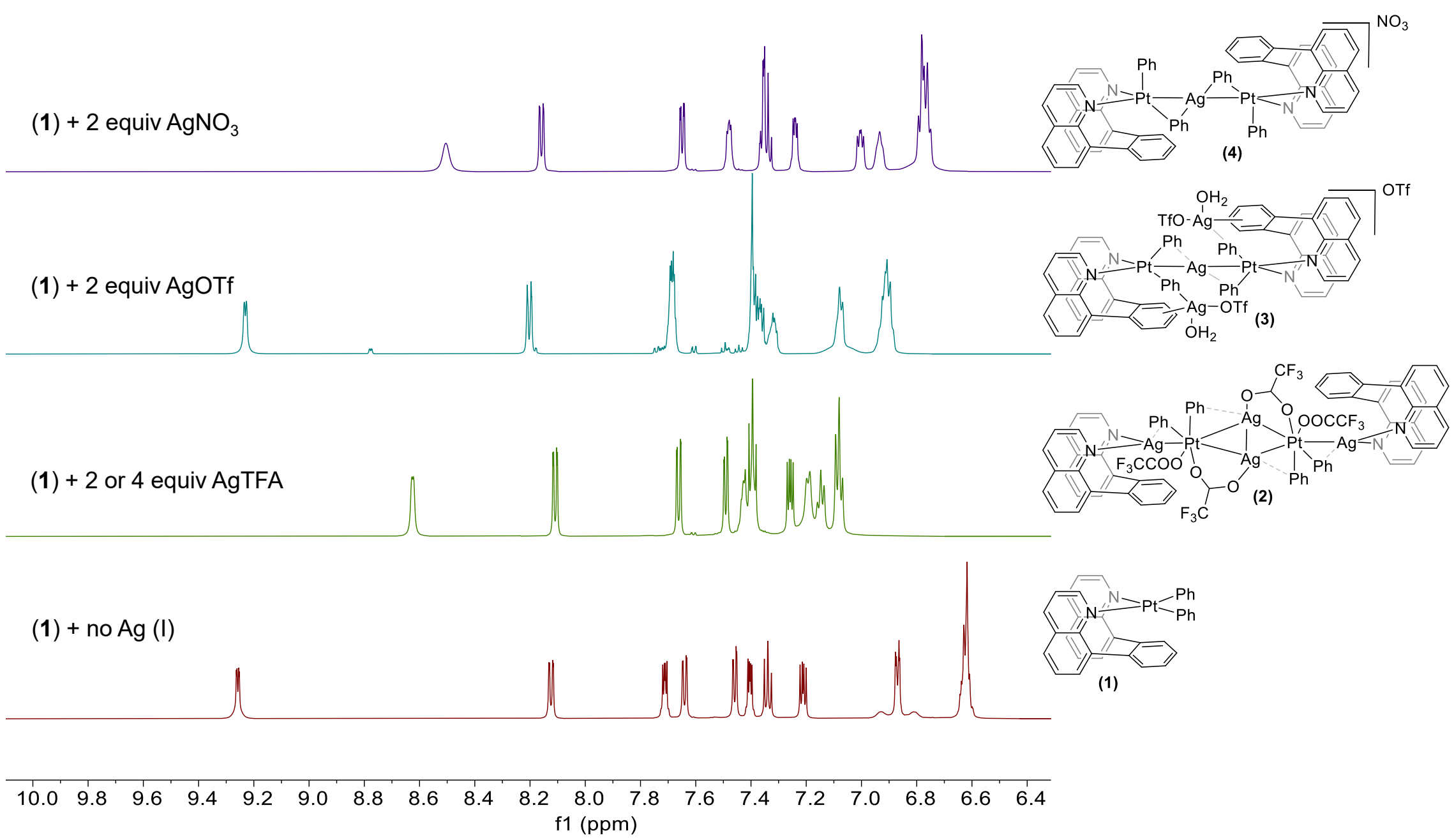

Figure S7. ^{1}H NMR spectra of complex (6-Fp)$PtPh_2$ (**1**) with 2 equivalents of AgTFA, AgOTf or $AgNO_3$ in CD_2Cl_2.

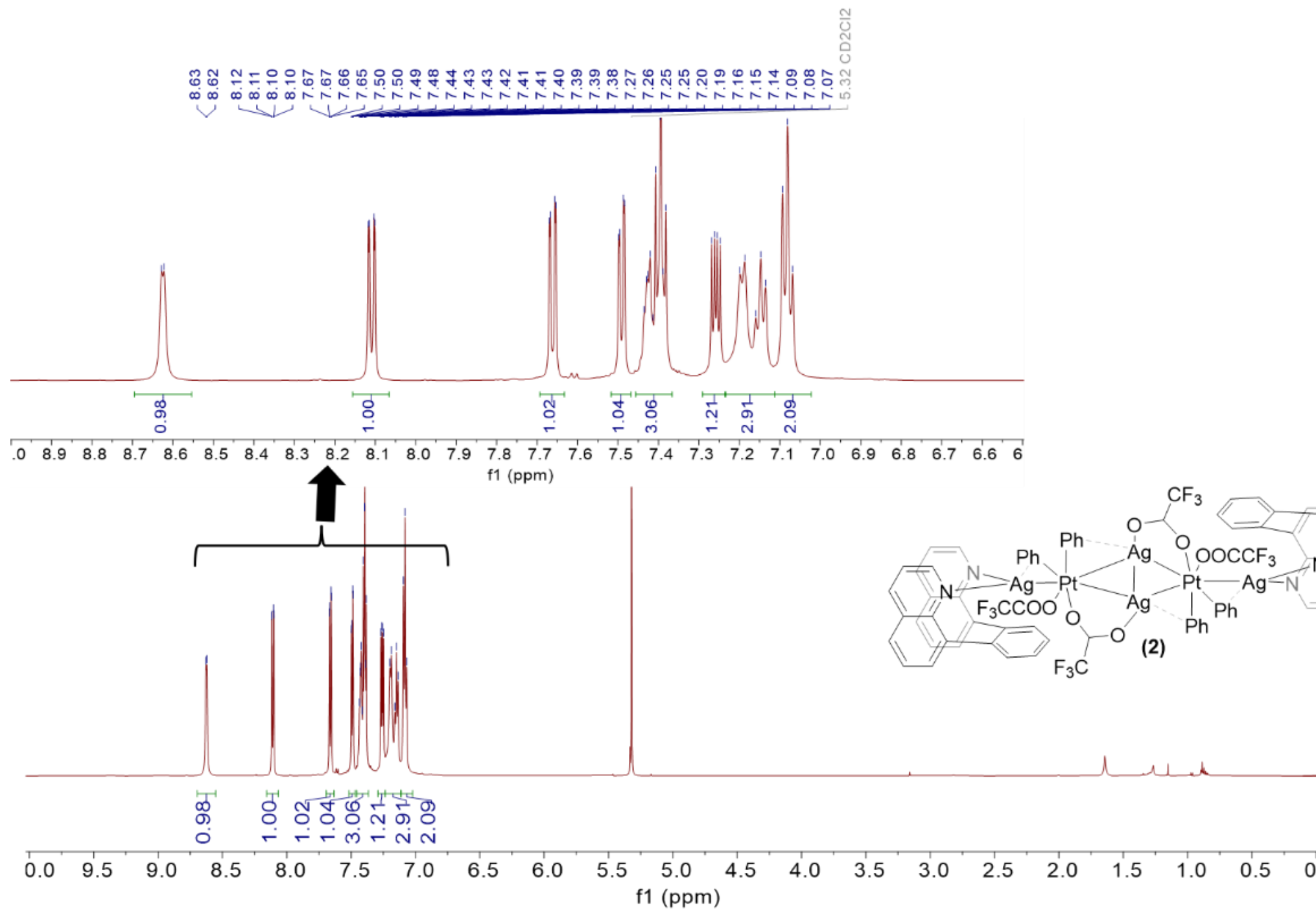

Figure S8. ^{1}H NMR spectrum of $[(6\text{-Fp})AgPtPh_2(TFA)(\mu\text{-TFA})Ag]_2$ (**2**) obtained from the reaction of (6-Fp)$PtPh_2$ (**1**) with 4 equiv of AgTFA in CD_2Cl_2.

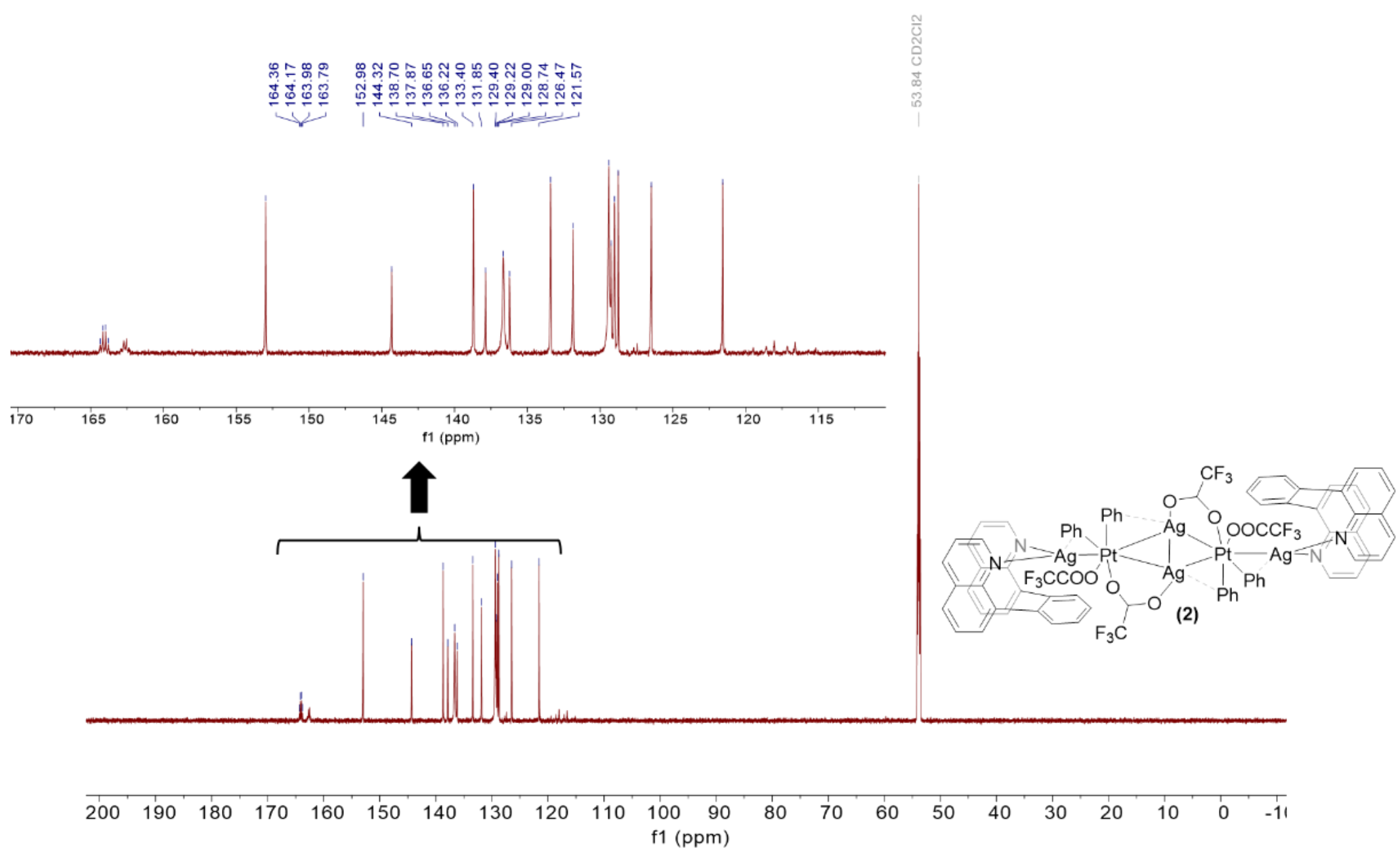

Figure S9. ^{13}C NMR spectrum of $[(6\text{-Fp})AgPtPh_2(TFA)(\mu\text{-TFA})Ag]_2$ (**2**) obtained from the reaction of (6-Fp)$PtPh_2$ (**1**) with 4 equiv of AgTFA in CD_2Cl_2.

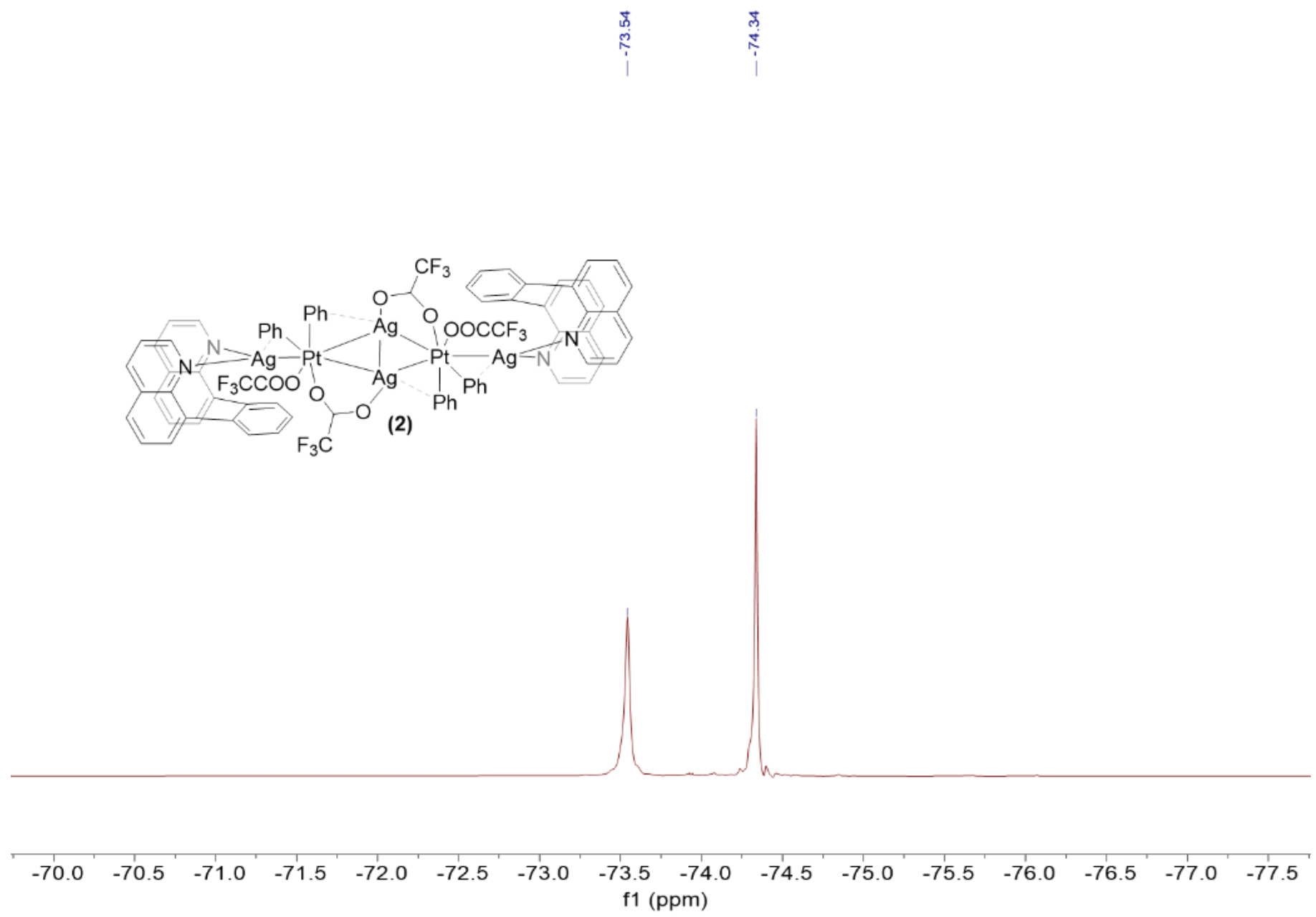

Figure S10. ^{19}F NMR spectrum of [(6-Fp)AgPtPh$_2$(TFA)(μ-TFA)Ag]$_2$ (**2**) obtained from the reaction of (6-Fp)PtPh$_2$ (**1**) with 4 equiv of AgTFA in CD_2Cl_2.

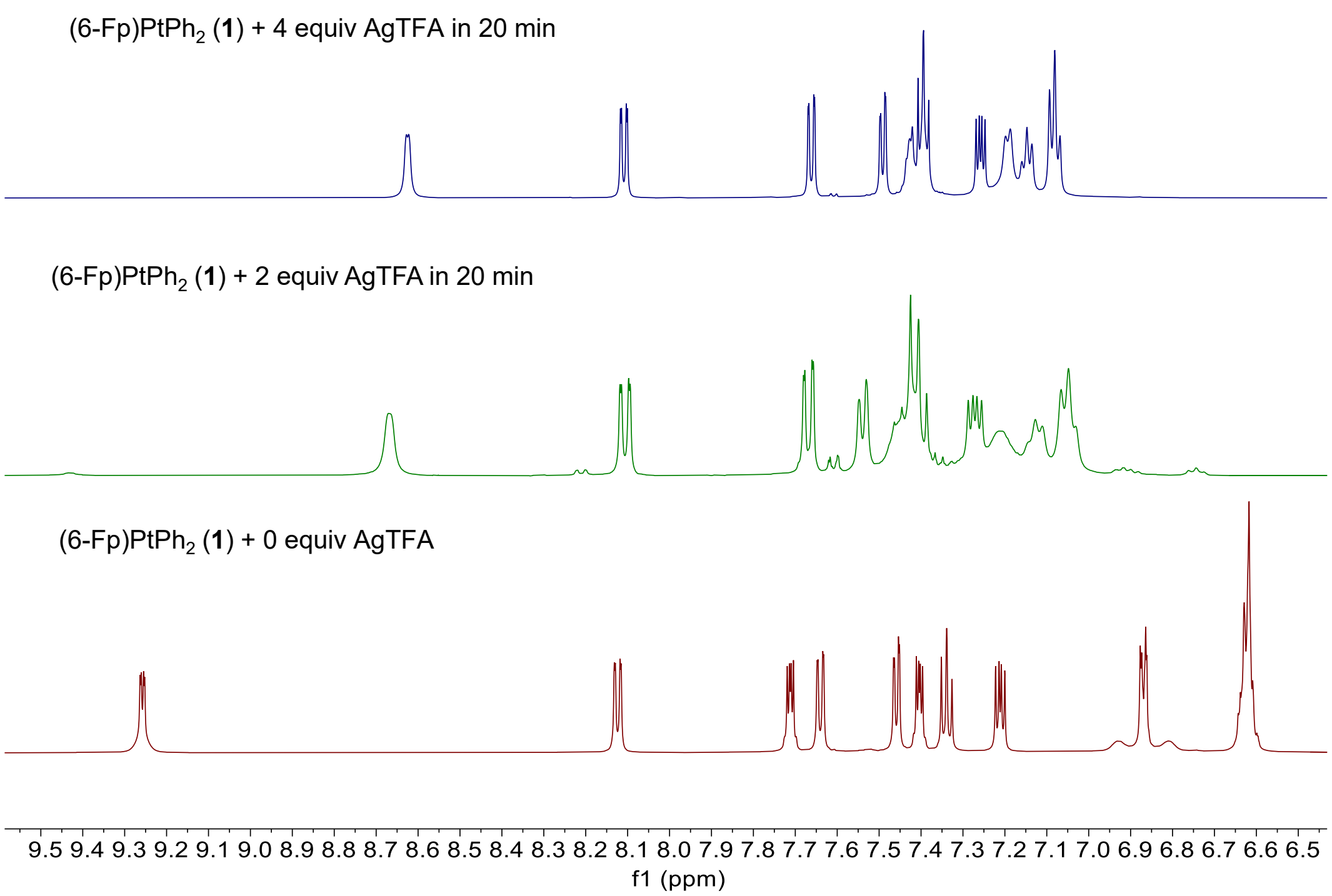

Figure S11. ^{1}H NMR spectra of [(6-Fp)AgPtPh$_2$(TFA)(μ-TFA)Ag]$_2$ (**2**) obtained from the reaction of (6-Fp)PtPh$_2$ (**1**) with 2 and 4 equiv of AgTFA in CD_2Cl_2.

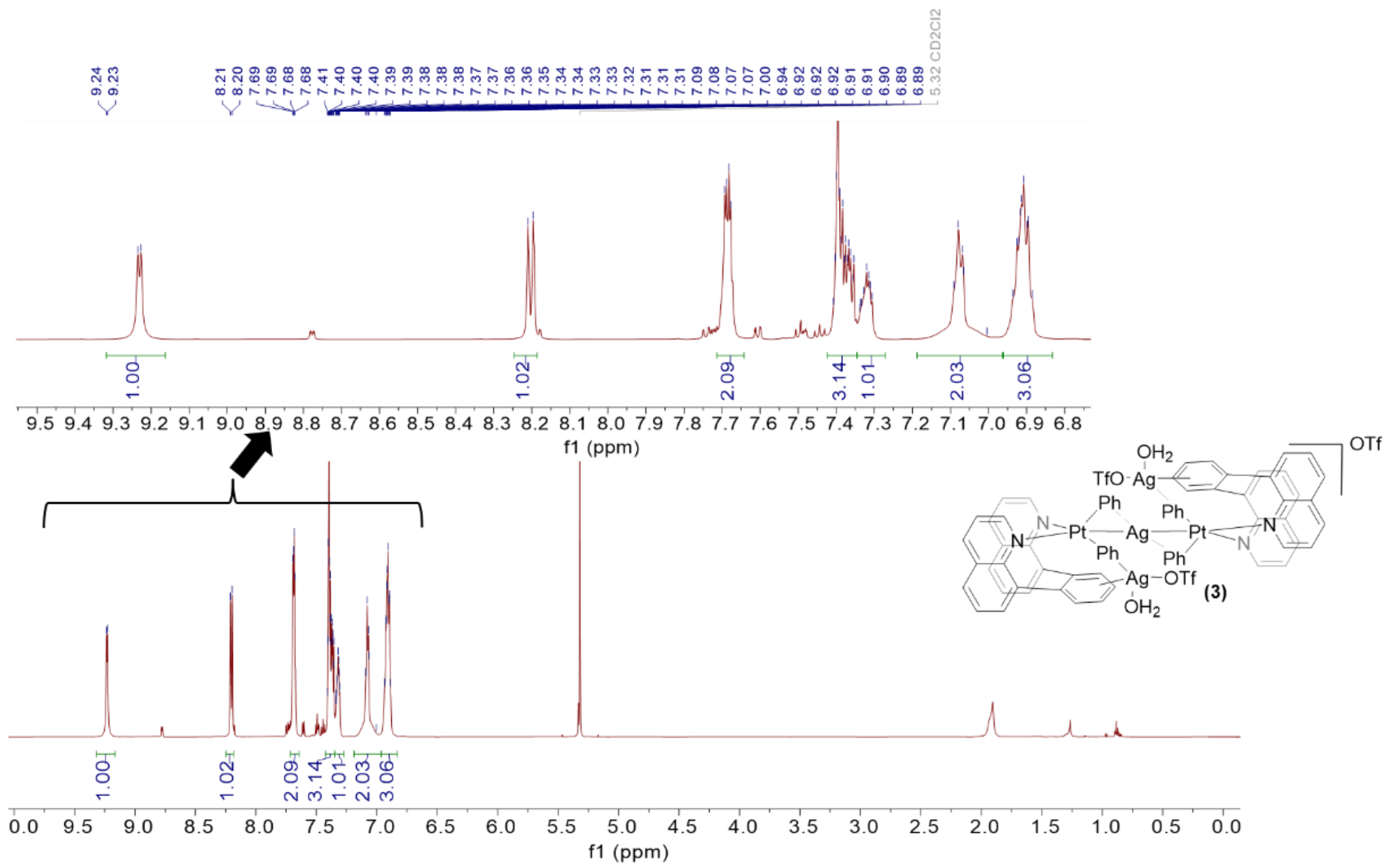

Figure S12. ^{1}H NMR spectrum of {[(6-Fp)$PtPh_2Ag(OTf)(H_2O)]_2$(μ-Ag)}OTf (**3**) obtained from the reaction of (6-Fp)$PtPh_2$ (**1**) with 2 equiv of AgOTf in CD_2Cl_2.

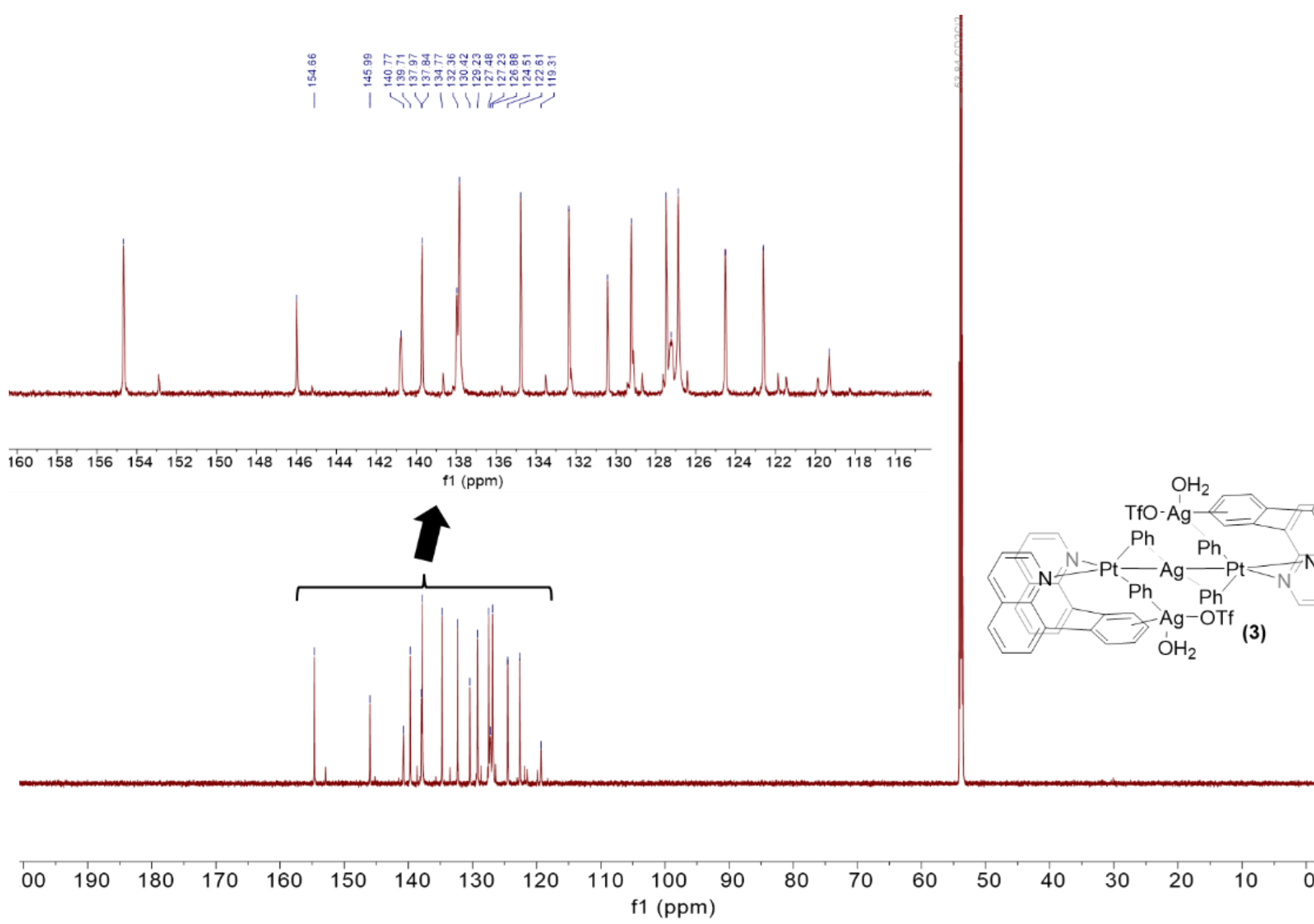

Figure S13. ^{13}C NMR spectrum of {[(6-Fp)$PtPh_2Ag(OTf)(H_2O)]_2$(μ-Ag)}OTf (**3**) obtained from the reaction of (6-Fp)$PtPh_2$ (**1**) with 2 equiv of AgOTf in CD_2Cl_2.

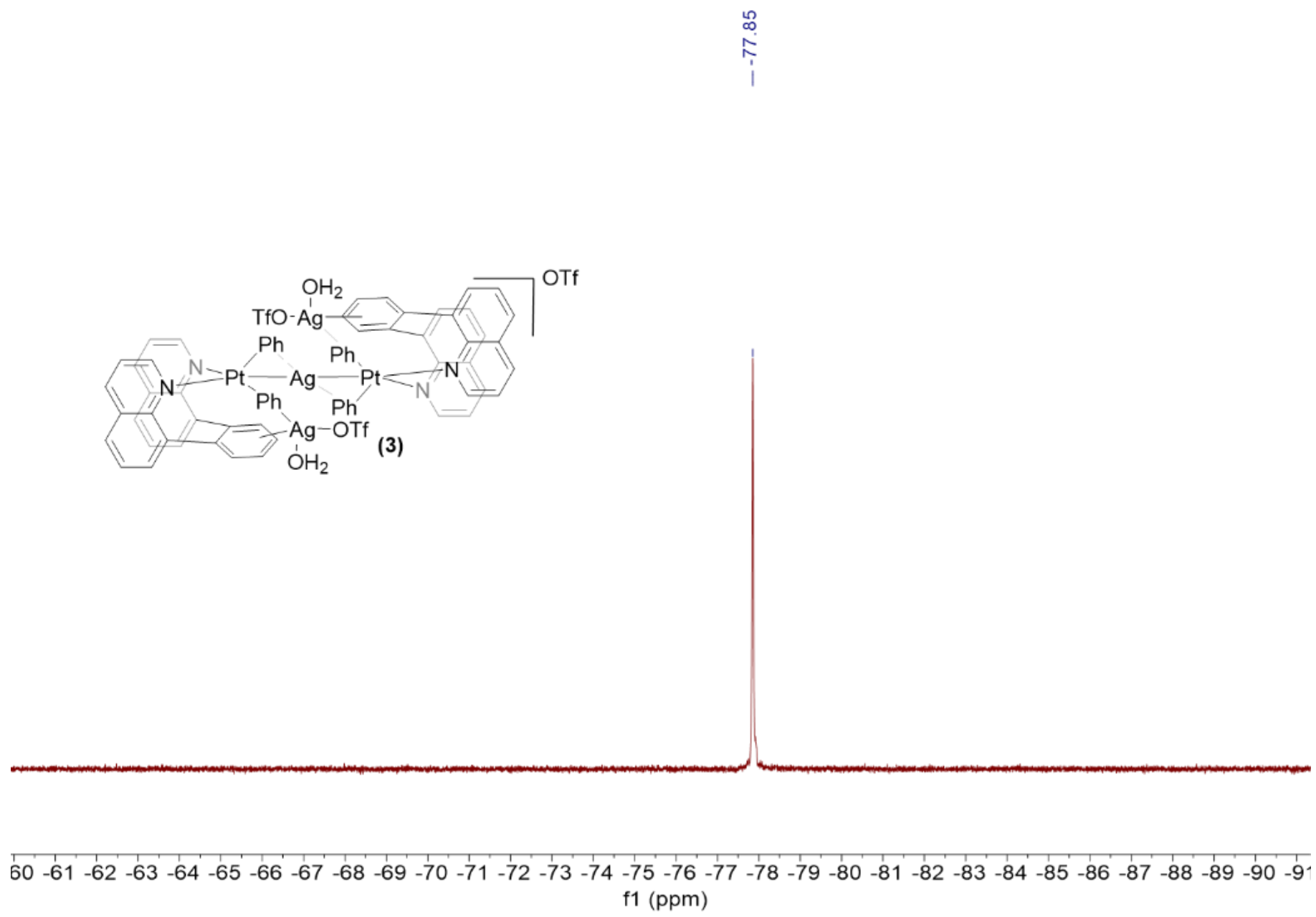

Figure S14. ^{19}F NMR spectrum of {[(6-Fp)PtPh$_2$Ag(OTf)(H$_2$O)]$_2$(*μ*-Ag)}OTf (**3**) obtained from the reaction of (6-Fp)PtPh$_2$ (**1**) with 2 equiv of AgOTf in CD_2Cl_2.

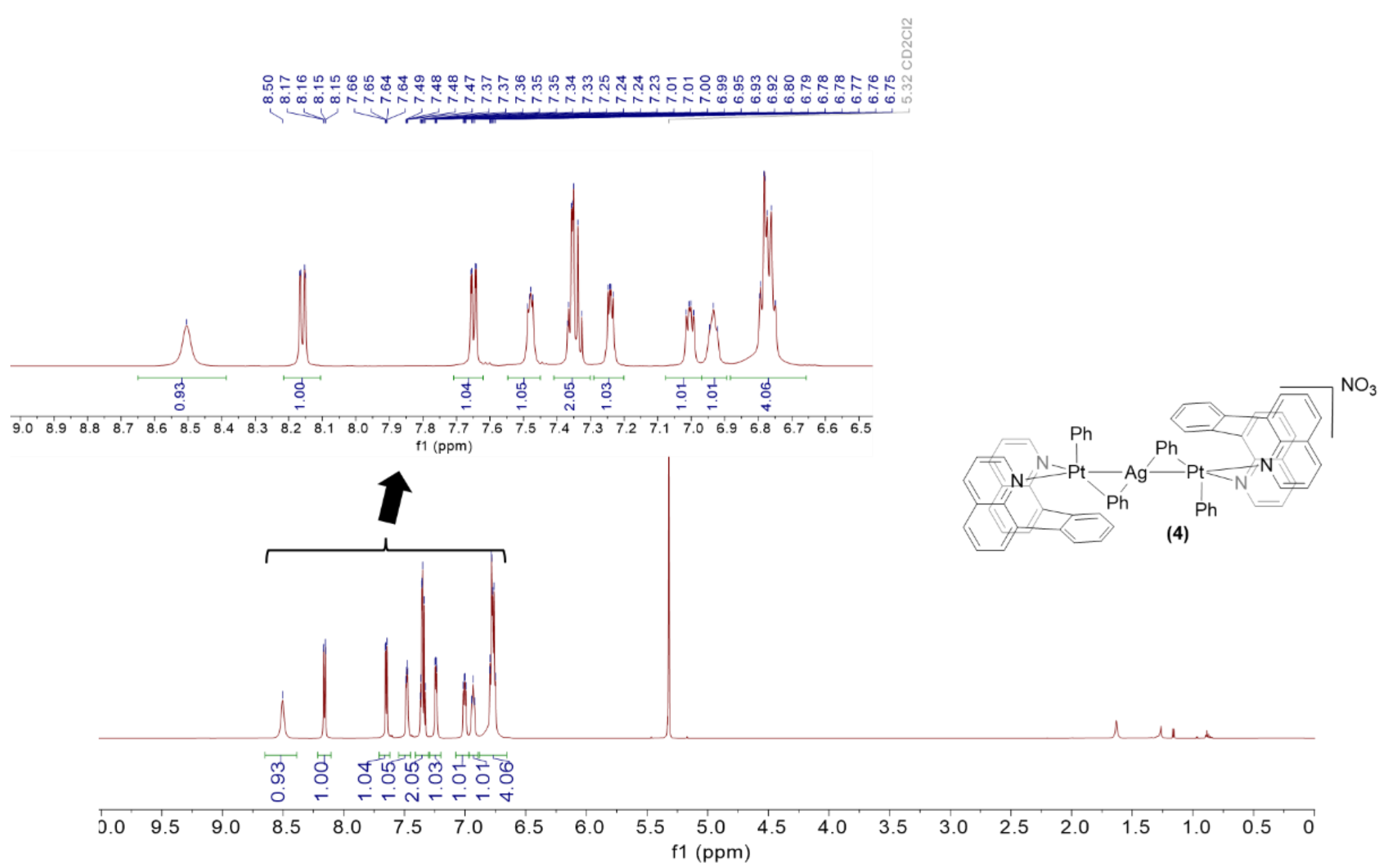

Figure S15. ^{1}H NMR spectrum of {[(6-Fp)PtPh$_2$]$_2$(μ-Ag)}NO$_3$ (**4**) obtained from the reaction of (6-Fp)PtPh$_2$ (**1**) with 2 equiv of AgNO$_3$ in CD_2Cl_2.

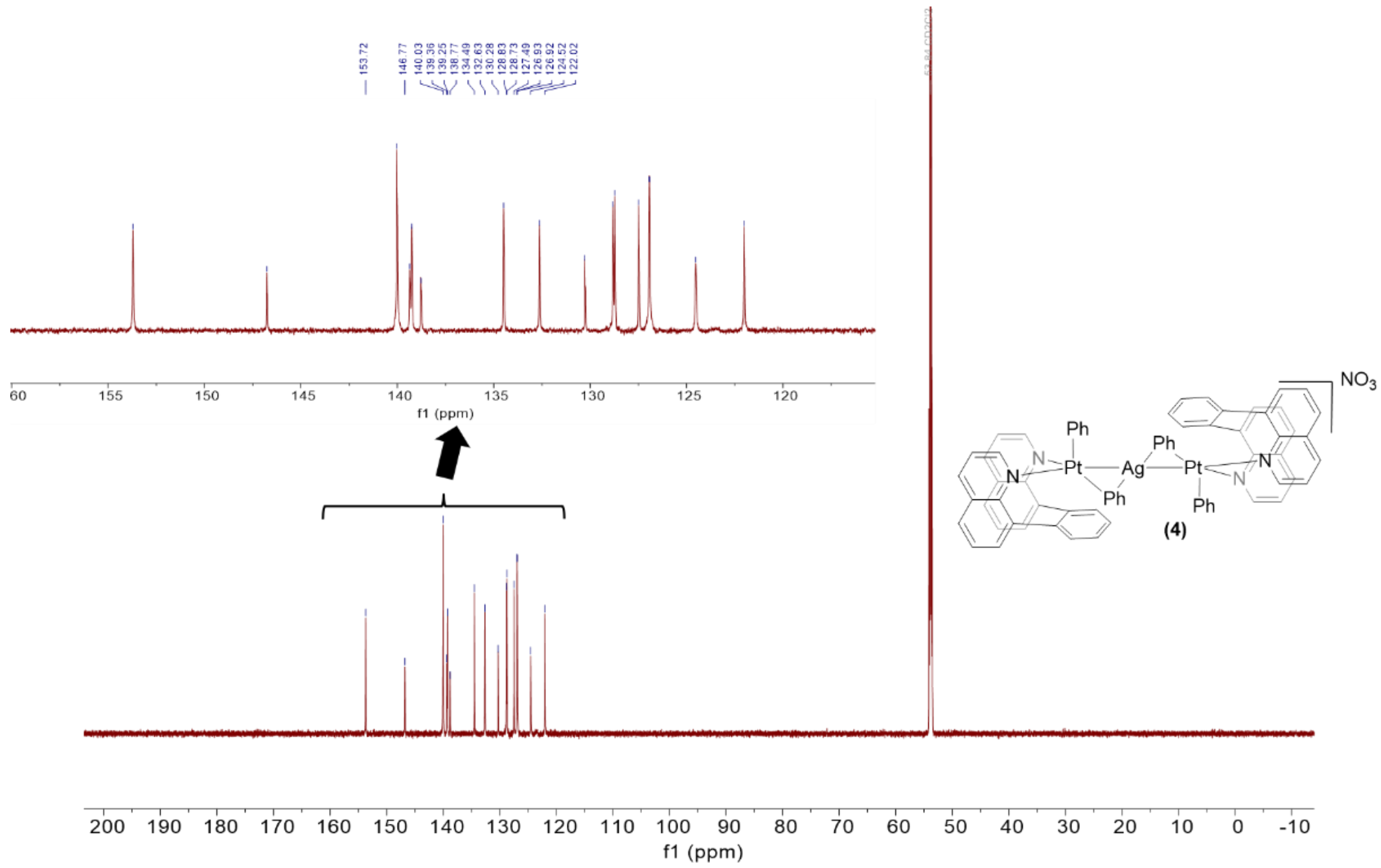

Figure S16. ^{13}C NMR spectrum of $\{[(6\text{-Fp})PtPh_2]_2(\mu\text{-Ag})\}NO_3$ (**4**) obtained from the reaction of (6-Fp)$PtPh_2$ (**1**) with 2 equiv of $AgNO_3$ in CD_2Cl_2.

4.2 ^{195}Pt NMR analysis for the complexes

To further evaluate the structure of Pt-Ag bimetallic complexes, we employed a detailed solution-state NMR characterization. First, we conducted a systematic assignment of ^{1}H and ^{13}C NMR chemical shifts of (6-Fp)PtPh$_2$ (**1**) using the one dimensional (1D) ^{1}H NMR relative integrated intensities and two-dimensional (2D) ^{1}H-^{1}H NOESY, ^{1}H-^{1}H COSY, ^{1}H-^{13}C HSQC, and ^{1}H-^{13}C HMBC NMR spectra (**Figure S17**). The chemical shift assignments are shown below in **Table S1** and **Scheme S1** shows the corresponding atom labels.

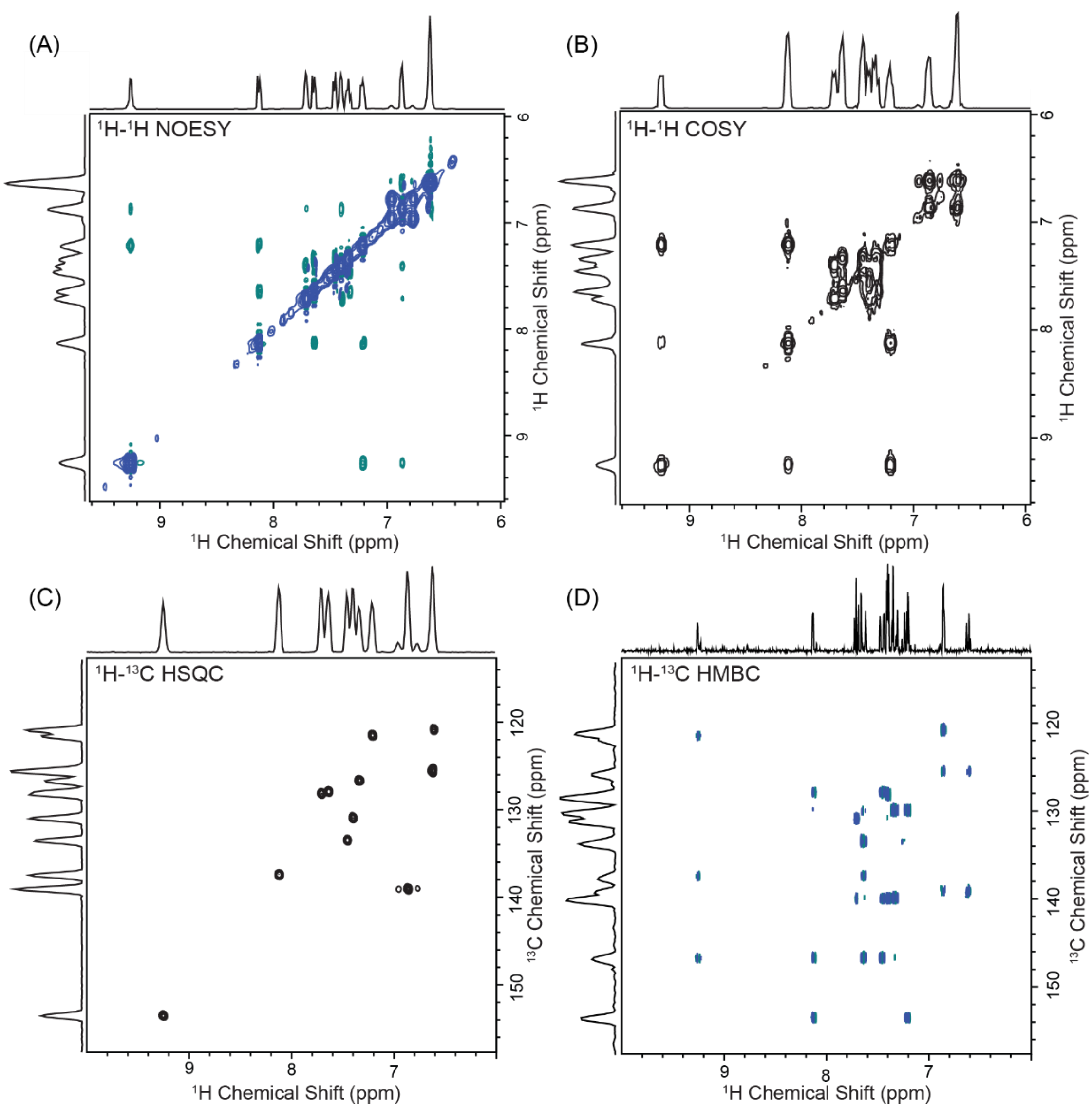

Figure S17. Two-dimensional (2D) (A) ^{1}H-^{1}H nuclear Overhauser effect spectroscopy (NOESY), (B) ^{1}H-^{1}H correlation spectroscopy (COSY), (C) ^{1}H-^{13}C heteronuclear single quantum coherence (HSQC), and (D)

^1H-^{13}C heteronuclear multiple bond coherence (HMBC) NMR spectra of (6-Fp)$PtPh_2$ (**1**) in CD_2Cl_2 solvent at 9.4 T.

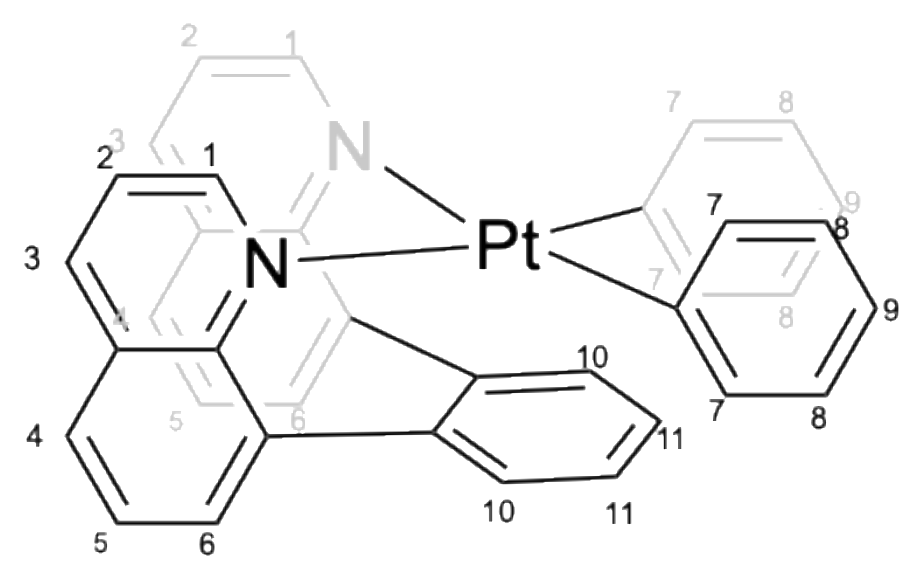

Scheme S1. Structure of (6-Fp)$PtPh_2$ (**1**) indicating atom labels (see **Table S1** for chemical shift assignments).

Table S1. ^1H and ^{13}C chemical shift assignments for all hydrogen atoms and protonated carbons in (6-Fp)$PtPh_2$ (**1**).

**Assignment**	**^1H shift (ppm)** #	**^{13}C shift (ppm)** #
8/9	6.62	121.1 (C8), 125.7 (C9)
7	6.87	139.2
2	7.21	121.7
5	7.34	126.8
10	7.40	131.1
6	7.46	133.7
4	7.64	128.1
11	7.71	128.3
3	8.12	137.6
1	9.26	153.7

#NMR chemical shifts were referenced with respect to residual ^1H and ^{13}C solvent signals at 5.32 ppm and 53.84 ppm, respectively.[4]

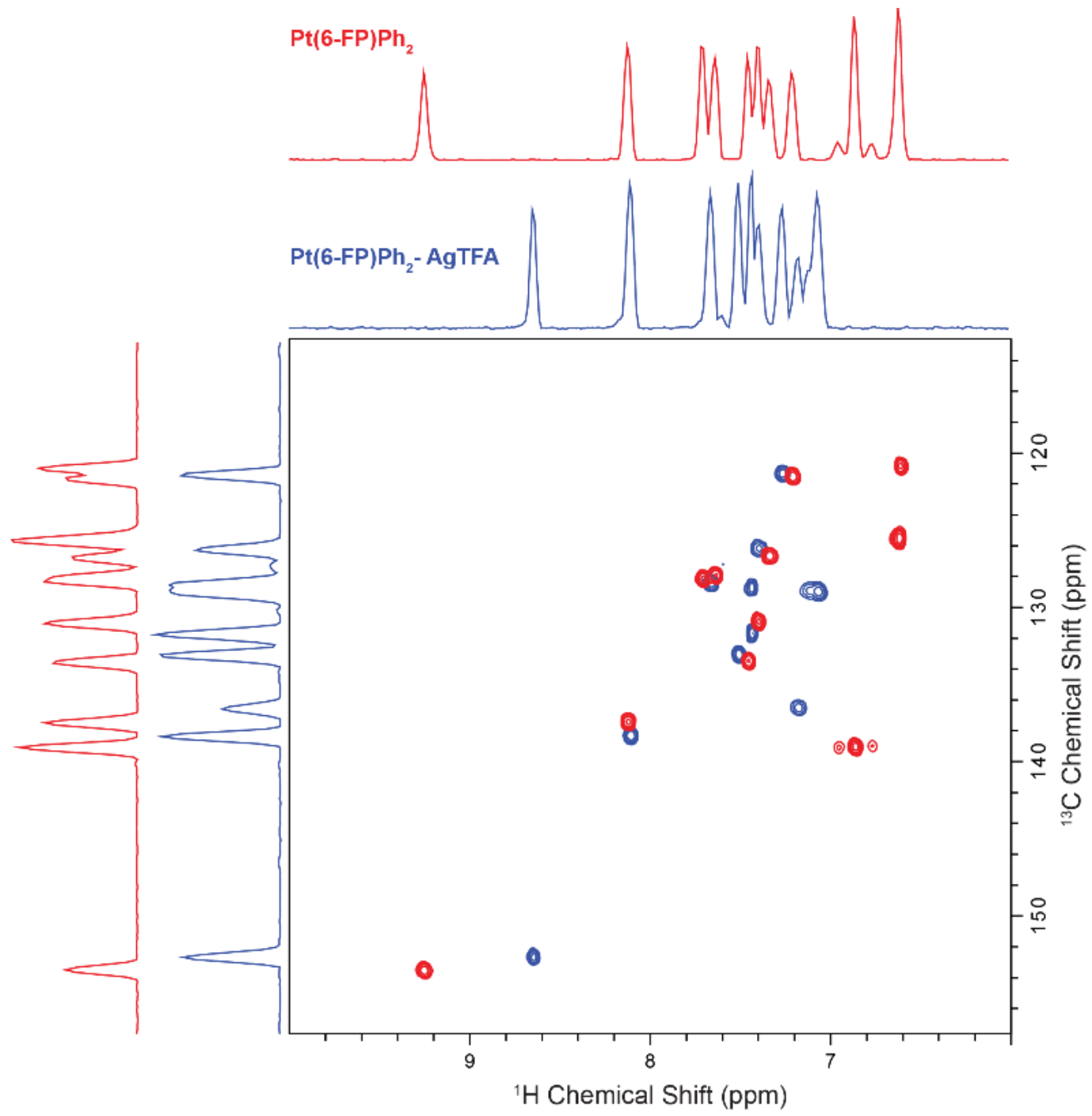

Figure S18. Overlay of 2D ^{1}H-^{13}C HSQC of (6-Fp)PtPh$_2$ (**1**) and [(6-Fp)AgPtPh$_2$(TFA)(μ-TFA)Ag]$_2$ (**2**), in CD_2Cl_2 solvent at 9.4 T.

Figure S18 compares the ^{1}H-^{13}C HSQC spectrum of **1** and [(6-Fp)AgPtPh$_2$(TFA)(μ-TFA)Ag]$_2$ (**2**). The one-to-one correspondence of ^{1}H and ^{13}C chemical shifts in both compounds agree strongly with the solid-state crystal structure of **2**, where one 6-Fp and two Ph ligands per Pt atom are still intact. Notably, the phenyl moieties showed the strongest perturbations in the ^{1}H and ^{13}C chemical shifts in **2**, which is also in good agreement with the significant changes in the phenyl group orientations, and close proximity to both Pt and Ag atoms (see **Table S1** for chemical shift assignments).

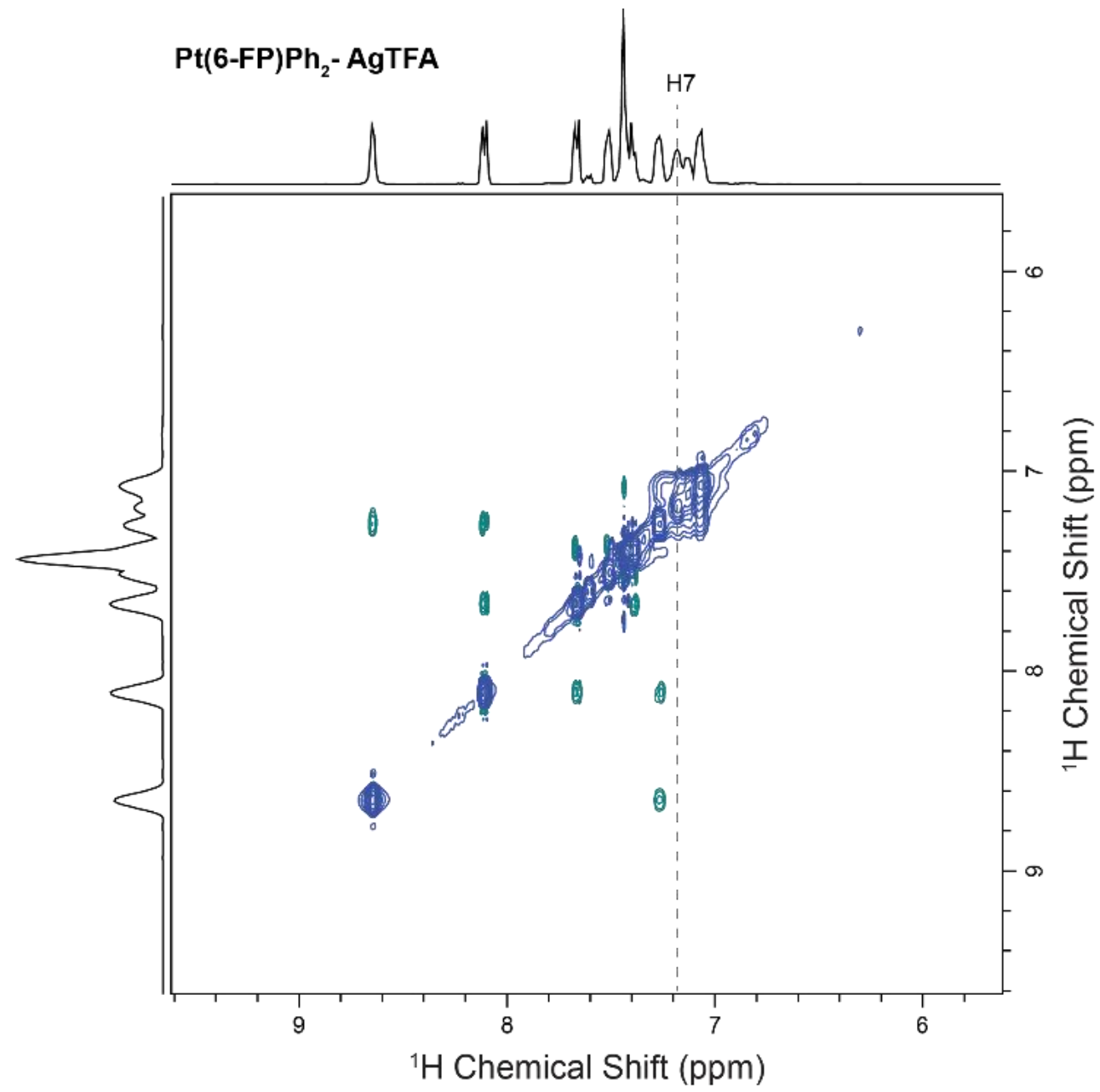

Figure S19. 2D ^1H-^1H NOESY spectrum of [(6-Fp)AgPtPh$_2$(TFA)(μ-TFA)Ag]$_2$ (**2**), in CD_2Cl_2 solvent at 9.4 T. Dashed line indicates no NOESY cross peaks for H7 at ca. 7.19 ppm.

Figure S19 shows the ^1H-^1H NOESY spectrum of **2**. The ^1H-^1H NOESY spectrum of **1** showed a stronger cross peak between H7 and H10, and a weaker cross peak between H7 and H11; these cross peaks indicated the close spatial proximity between the ortho hydrogen atoms in the phenyl groups (H7), to the arene ring below the Pt atom (**Figure S17**). Notably, these cross peaks are absent upon formation of the Pt-Ag complex with AgTFA (**Figure S19**), suggesting that the arene hydrogen atoms are further away in space from both the phenyl groups, and the ortho hydrogens of the pyridine units in the 6-Fp ligand. This conclusion is in line with the solid-state single-crystal X-ray diffraction structure. Analogous one-to-one correspondence in ^1H and ^{13}C chemical shifts supports the formation of **3** and **4**. While [(6-Fp)AgPtPh$_2$(TFA)(μ-TFA)Ag]$_2$ was stable in solution after storage at –20 °C, the corresponding AgOTf (**3**) and $AgNO_3$ (**4**) compounds were less stable over a period of a several days to a few weeks.

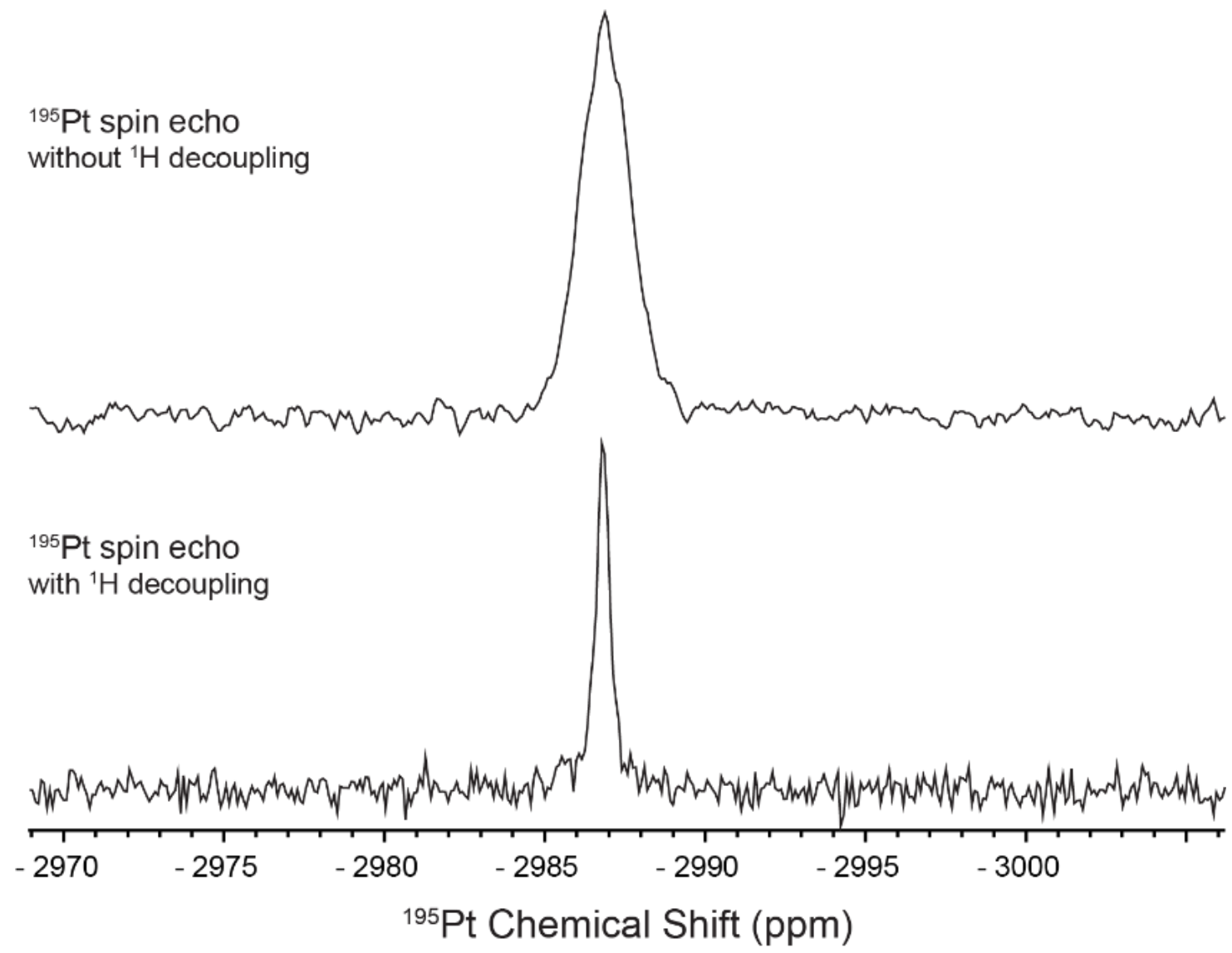

Figure S20. ^{195}Pt spin echo NMR spectra of (6-Fp)PtPh$_2$ (**1**), in CD_2Cl_2 solvent obtained with (bottom trace) and without (top trace) ^{1}H decoupling at 14.1 T.

Next, we obtained ^{195}Pt spin echo spectra of **1** using a simple π/2-τ-π-τ-echo pulse sequence (**Figure S20**). The spin echo pulse sequence was used to avoid probe ringing which results in baseline artifacts. ^{195}Pt NMR spectra obtained without ^{1}H decoupling showed a broad multiplet, which collapsed to a narrower signal with ^{1}H decoupling as expected. The ^{195}Pt NMR signal appears at –2987 ppm.

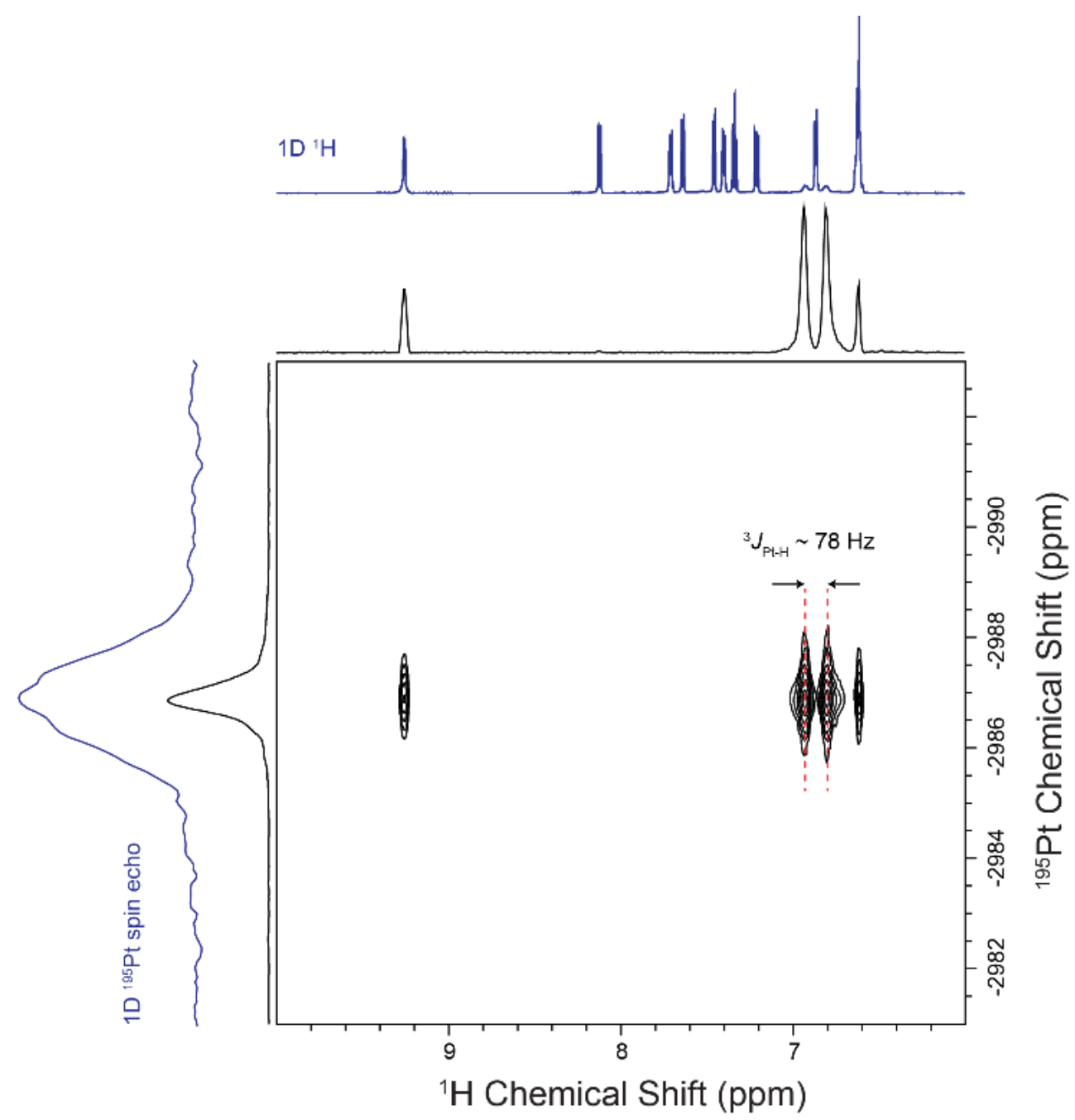

Figure S21. $^1H\{^{195}Pt\}$ heteronuclear single quantum coherence (HSQC) NMR spectrum of (6-Fp)$PtPh_2$ (**1**), in CD_2Cl_2 solvent at 14.1 T. The spectrum was obtained without ^{195}Pt decoupling which shows the $^3J_{Pt\text{-}H}$ of *ca.* 78 Hz in the ^1H dimension (F_2). 1D ^1H and ^{195}Pt NMR spectra are shown above the projections (in blue).

To confirm the coupling of the ^{195}Pt center to multiple protons, as observed in the 1D ^1H NMR spectrum of **1**, we obtained a ^1H-^{195}Pt HSQC NMR spectrum (**Figure S21**). Here, we clearly observed a doublet splitting in the ortho hydrogens of the phenyl groups, corresponding to a $^3J_{Pt\text{-}H}$ of 78 Hz. As described in the main text, the ortho hydrogens of the pyridine units in the 6-Fp ligand also showed a coupling to ^{195}Pt, but this was less clearly resolved suggesting a weak $^3J_{Pt\text{-}H}$ coupling on the order of 15-20 Hz. Note that the satellites in the ^1H NMR spectrum corresponding to the ^1H-^{195}Pt *J*-couplings are significantly broadened due to fast ^{195}Pt longitudinal relaxation times (T_1), likely due to large chemical shift anisotropy typically observed with square planar Pt complexes. We confirmed the fast ^{195}Pt T_1 relaxation time using an inversion recovery experiment, which showed a ^{195}Pt T_1 of ≈ 13 ms. The short ^{195}Pt T_1 relaxation time is on the order of $1/^3J_{Pt\text{-}H}$, which explains the observed line broadening and poor resolution of the *J*-splittings.

To directly probe the local environment of the Pt centers in the Pt-Ag complexes, we obtained ^{195}Pt spin echo spectra of [(6-Fp)$AgPtPh_2$(TFA)(μ-TFA)Ag]$_2$ (**2**), {[(6-

Fp)$PtPh_2Ag(OTf)(H_2O)]_2$(*μ*-Ag)}OTf (**3**), and {[(6-Fp)$PtPh_2]_2$(μ-Ag)}NO_3 (**4**) (**Figure S22**). These spectra were obtained without ^{1}H decoupling to minimize sample heating and enable rapid scanning. First, in all Pt-Ag compounds, we observe significant chemical shifts in comparison to **1**, which supports the changes in structure discussed above. Compounds **3** and **4** show ^{195}Pt NMR signals at –2638 ppm and –2627 ppm, respectively. According to the single crystal X-ray structures, **3** and **4** exhibit similar Pt coordination environments where each Pt is coordinated to two nitrogen atoms in the 6-Fp ligand, two phenyl groups, and one Ag atom. This 5-coordinate environment and presence of Ag in the local coordination sphere, results in a similar increase in chemical shift by ca. 350 ppm for both **3** and **4** in comparison to **1**. Previously, the presence of one Pt-Ag interaction showed a ca. 200 ppm increase in ^{195}Pt NMR chemical shift, whereas two Pt-Ag interactions showed a ca. 400 ppm increase in the ^{195}Pt NMR chemical shift.[5] The average increase in ^{195}Pt NMR chemical shift by ca. 350 ppm observed here is consistent with two Pt-Ag interactions in compounds **3** and **4**, in good agreement with the single crystal X-ray structures.

In compound **2** the local coordination environment of Pt is more significantly affected upon addition of AgTFA. The single crystal structure shows each Pt atom is coordinated to three Ag atoms, two phenyl groups, and two CF_3COO groups, resulting in a coordination number of seven, and a total of six Pt-Ag interactions. Following the trend observed with **3** and **4**, the ^{195}Pt chemical shift of **2** increased further to –2460 ppm (relatively narrow, intense signal), in comparison to **1**. However, a second weaker and broader signal was observed at ca. –2200 ppm which is tentatively assigned to an unidentified Pt-complex obtained after biphenyl elimination. In all cases, we do not observe scalar couplings between ^{195}Pt and ^{109}Ag, likely due to fast relaxation of ^{195}Pt and potentially also the coupled ^{109}Ag and $^{107/109}Ag$ spins. Furthermore, the short ^{195}Pt T_1 and T_2 relaxation times also precluded the acquisition of ^{1}H-^{195}Pt HSQC spectra with compounds **2**, **3** and **4**.

Finally, ^{1}H-^{15}N HMBC spectra of **1**, **2** and **3** showed changes in ^{15}N chemical shifts upon formation of Pt-Ag complexes with an increase in ^{15}N chemical shift of ≈ 15 ppm observed for **2** due to N-Ag interactions (**Figure S23**). Notably, with **2** and **3**, a single ^{15}N

resonance is observed, in good agreement with the ^{1}H and ^{13}C NMR data. The lack of ^{15}N-^{195}Pt or ^{15}N-$^{107/109}Ag$ *J*-couplings may be due to the fast relaxation effects described earlier, or potentially due to dynamic, labile N-metal interactions. Cumulatively, these results support the formation of the proposed Pt-Ag compounds in solution in good agreement with the X-ray crystal structures.

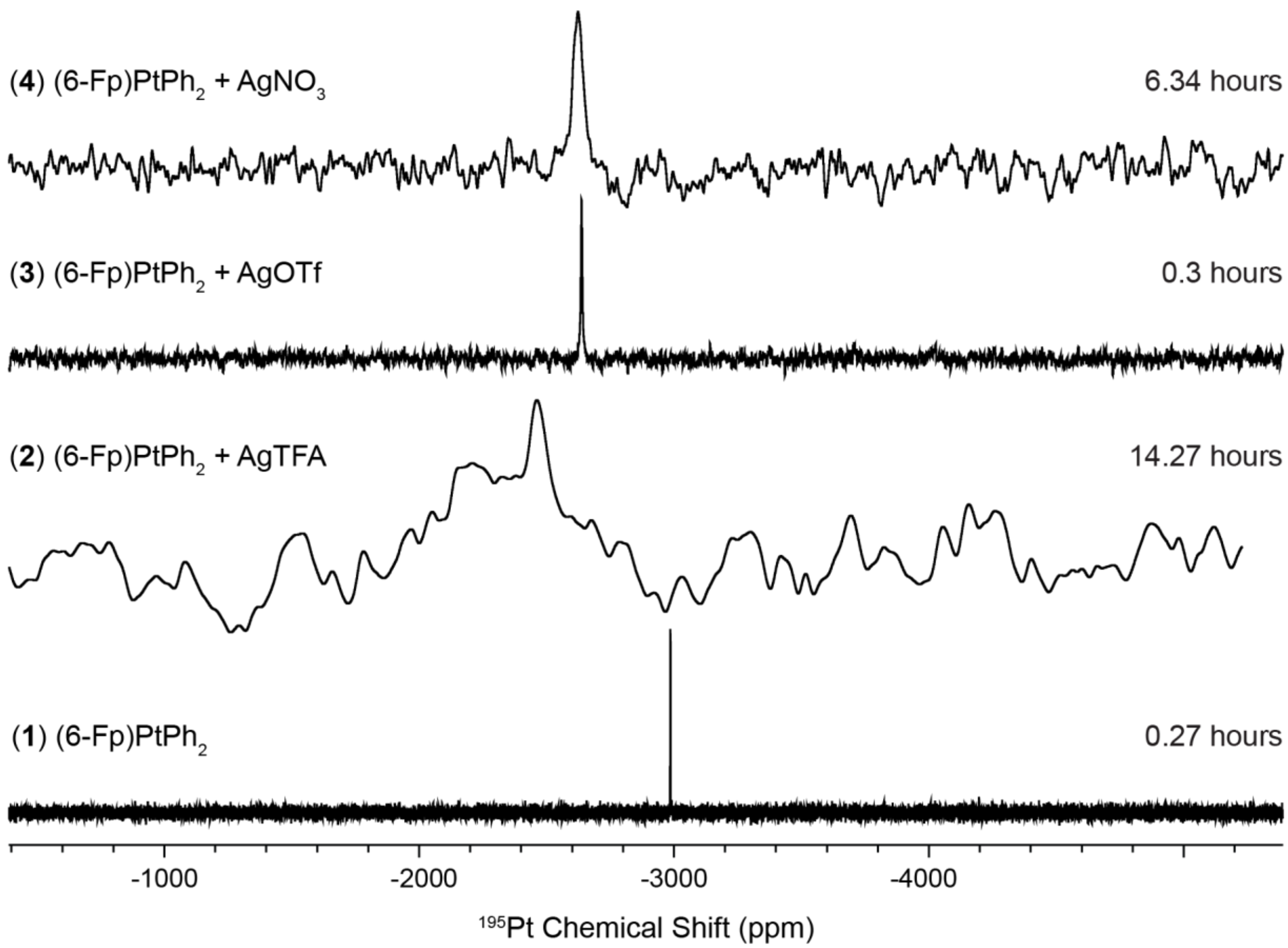

Figure S22. ^{195}Pt spin echo NMR spectra of (6-Fp)$PtPh_2$ (**1**), [(6-Fp)$AgPtPh_2$(TFA)(μ-TFA)Ag]$_2$ (**2**), {[(6-Fp)$PtPh_2$Ag(OTf)(H_2O)]$_2$(*μ*-Ag)}OTf (**3**), {[(6-Fp)$PtPh_2$]$_2$(μ-Ag)}NO_3 (**4**), in CD_2Cl_2 solvent at 14.1 T. Spectra were obtained without ^{1}H decoupling to minimize sample heating. Short recycle delays were used for rapid scanning as the measured T_1 relaxation time of **1** was short (~ 13 ms).^{195}Pt NMR Spectra were indirectly referenced using the ^{1}H CD_2Cl_2 residual solvent signal and IUPAC frequency ratios.[6]

Table S2. Experimental and processing parameters of ^{195}Pt spin echo NMR spectra obtained at 14.1 T.

	**1**	**2**	**3**	**4**
Number of scans	2048	414720	8588	184320
Recycle delay	0.2	0.05	0.05	0.05

π/2 pulse length (μs)	14.75	14.75	14.75	14.75
π pulse length (μs)	29.5	29.5	29.5	29.5
Half-echo delay, τ (μs)	200	50	50	50
Acquisition time	0.2457600	0.0436907	0.0436907	0.0436907
Total experimental time (hours)	0.27	14.27	0.30	6.34
Spectral width	750000	750000	750000	750000
Number of points in time domain	65536	65536	65536	65536
Line broadening (Hz)	25	7500	250	1500

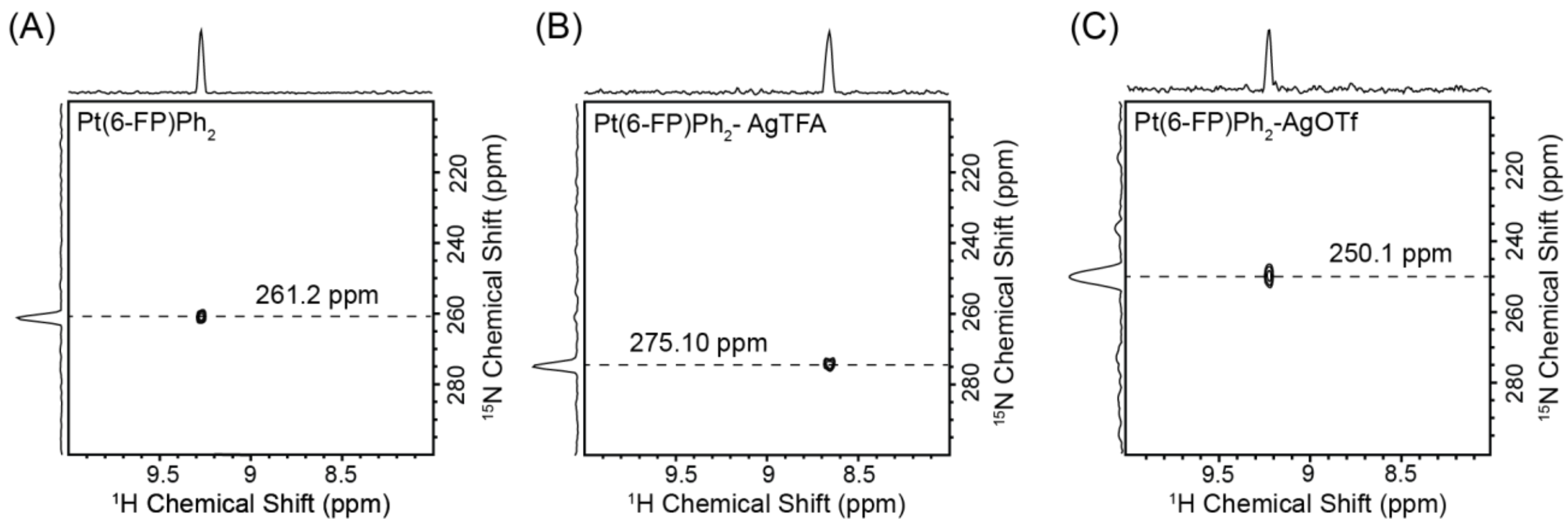

Figure S23. ^{1}H-^{15}N HMBC spectra of (6-Fp)$PtPh_2$ (**1**), [(6-Fp)$AgPtPh_2$(TFA)(μ-TFA)Ag]$_2$ (**2**), and {[(6-Fp)$PtPh_2$Ag(OTf)(H_2O)]$_2$(*μ*-Ag)}OTf (**3**), in CD_2Cl_2 solvent at 14.1 T. ^{195}Pt NMR Spectra were indirectly referenced using the ^{1}H CD_2Cl_2 residual solvent signal and IUPAC frequency ratios.6

4.3 Solid state characterization (single crystal X-ray diffraction)

A single crystal of (6-Fp)$PtPh_2$ (**1**), [(6-Fp)$AgPtPh_2$(TFA)(μ-TFA)$Ag]_2$ (**2**), {[(6-Fp)$PtPh_2Ag$(OTf)(H_2O)$]_2$(*μ*-Ag)}OTf (**3**), {[(6-Fp)$PtPh_2]_2$(μ-Ag)}NO_3 (**4**) and (6-Fp)Ag_3TFA_3 (**5**) were coated with Paratone oil and mounted on a MiTeGen micromount. Data were collected on a Bruker D8 VENTURE dual wavelength Mo/Cu Kappa four-circle diffractometer using a PHOTON III detector. The diffractometer was equipped with an Oxford Cryostream 800Plus low temperature device. The Mo K_α radiation (λ = 0.71073Å) from an Incoatec IμS 3.0 microfocus sealed X-ray tube with a HELIOS double bounce multilayer mirror as monochromator was used for each crystal except **4**. The data collection for **4** was done with Cu K_α radiation (λ = 1.54178Å) from an Incoatec IμS 3.0 microfocus sealed tube with a HELIOS EF double bounce multilayer mirror as monochromator.

All data collection and processing were done within the APEX6 software suite.[7] All data were integrated with SAINT using a narrow-frame algorithm and a Multi-Scan absorption correction using SADABS was applied.[8] Using Olex2 as a graphical interface,[9] each structure was solved by dual methods with SHELXT[10] and refined by full-matrix least-squares methods against F^2 using SHELXL.[11] All non-hydrogen atoms were refined with anisotropic displacement parameters. Hydrogen atoms in all structures were placed in calculated positions using a riding model with their U_{iso} values constrained to 1.5 times the U_{eq} of their pivot atoms for terminal sp^3 carbon atoms and 1.2 times for all other carbon atoms. Each CIF file was generated using FinalCif.[12]

For **1**, the Pt atom was modeled as disordered over two sites, but the occupancy of the second site was only about 3%, so it was not possible to model the disorder in the ligand although it must also be present. For **2**, **3**, and **4** the relative occupancy of the disordered atoms was freely refined. Constraints and restraints were used as needed on the anisotropic displacement parameters and/or bond lengths of the disordered atoms. For **4**, disordered solvent was accounted for with the OLEX2 solvent mask algorithm. It found void spaces of 313 Å^3 containing 76 electrons. This corresponds to a 1:1 mixture of Et_2O and CH_2Cl_2.

Table S3. Crystal data for complexes (6-Fp)PtPh$_2$ (**1**), [(6-Fp)AgPtPh$_2$(TFA)(μ-TFA)Ag]$_2$ (**2**), {[(6-Fp)PtPh$_2$Ag(OTf)(H$_2$O)]$_2$(*μ*-Ag)}OTf (**3**), {[(6-Fp)PtPh$_2$]$_2$(μ-Ag)}NO$_3$ (**4**) and (6-Fp)Ag$_3$TFA$_3$ (**5**).

	**1**	**2**	**3**	**4**	**5**
CCDC number	2548764	2548765	2548766	2548767	2548768
Empirical formula	$C_{36}H_{26}N_2Pt$	$C_{80}H_{52}Ag_4F_{12}N_4O_8Pt_2$	$C_{77}H_{60}Ag_3Cl_4F_9N_4O_{11}Pt_2S_3$	$C_{77}H_{64}AgCl_2N_5O_4Pt_2$	$C_{30}H_{16}Ag_3F_9N_2O_6$
Formula weight	681.68	2246.91	2340.06	1692.28	995.06
Temp [K]	100.00	100.00	100.00	100.00	100.00
Cryst. system	triclinic	Triclinic	Triclinic	Triclinic	triclinic
Space group	P -1	P -1	P -1	P -1	P -1
a [Å]	10.1421(4)	9.8887(3)	12.7097(4)	10.1483(2)	10.5353(4)
b [Å]	11.0056(5)	10.7016(4)	13.2419(5)	12.5491(5)	11.6159(4)
c [Å]	13.1406(6)	18.2926(6)	13.5270(5)	14.6612(3)	12.8850(5)
α [°]	72.956(2)	100.2110(10)	92.5890(10)	89.978(2)	86.1860(10)
β [°]	67.7410(10)	104.7520(10)	113.4370(10)	76.3270(10)	84.9040(10)
γ [°]	75.6210(10)	100.4140(10)	112.5870(10)	89.967(2)	73.0650(10)
Volume [Å^3]	1282.11(10)	1789.70(10)	1875.18(12)	1814.22(9)	1501.12(10)
Z	2	1	1	1	2
ρ_{calc} [gcm^{-3}]	1.766	2.085	2.072	1.549	2.201
μ [mm^{-1}]	5.502	5.059	4.800	10.281	2.043
F(000)	668	1076	1132	830	960
Crystal size [mm^3]	0.044×0.084×0.131	0.025×0.027×0.087	0.106×0.132×0.141	0.043×0.072×0.129	0.027×0.048×0.1
Crystal color	yellow	colorless	colorless	colorless	colorless
Crystal shape	plate	block	plate	plate	needle
λ [Å]	Mo $K_α$ (λ=0.71073 Å)	Mo $K_α$ (λ=0.71073 Å)	Mo $K_α$ (λ=0.71073 Å)	Cu $K_α$ (λ=1.54178 Å)	Mo $K_α$ (λ=0.71073 Å)
2Θ range [°]	3.92 to 59.19 (0.72 Å)	4.13 to 56.61 (0.75 Å)	3.89 to 66.31 (0.65 Å)	6.20 to 136.99 (0.83 Å)	4.05 to 56.64 (0.75 Å)
Index ranges	−14 ≤ h ≤ 12<br>−15 ≤ k ≤ 15<br>−18 ≤ l ≤ 18	−13 ≤ h ≤ 13<br>−14 ≤ k ≤ 14<br>−24 ≤ l ≤ 22	−19 ≤ h ≤ 19<br>−20 ≤ k ≤ 18<br>−20 ≤ l ≤ 20	−12 ≤ h ≤ 12<br>−15 ≤ k ≤ 15<br>−17 ≤ l ≤ 16	−14 ≤ h ≤ 14<br>−15 ≤ k ≤ 15<br>−17 ≤ l ≤ 17
Reflections collected	46906	64539	80413	34873	46643
Independent reflections	7179<br>R_{int} = 0.0574<br>R_{sigma} = 0.0352	8890<br>R_{int} = 0.0768<br>R_{sigma} = 0.0489	14301<br>R_{int} = 0.0318<br>R_{sigma} = 0.0240	6658<br>R_{int} = 0.0646<br>R_{sigma} = 0.0438	7462<br>R_{int} = 0.0521<br>R_{sigma} = 0.0358

Completeness [%]	99.9	99.9	99.9	99.9	99.9
Data / Restraints / Parameters	7179 / 0 / 362	8890 / 15 / 536	14301 / 43 / 588	6658 / 576 / 653	7462 / 0 / 451
Goodness-of-fit on F^2	1.097	1.020	1.026	1.074	1.025
Final R indexes [$I \geq 2\sigma(I)$]	R_1 = 0.0259<br>wR_2 = 0.0587	R_1 = 0.0307<br>wR_2 = 0.0628	R_1 = 0.0251<br>wR_2 = 0.0585	R_1 = 0.0445<br>wR_2 = 0.1120	R_1 = 0.0323<br>wR_2 = 0.0747
Final R indexes [all data]	R_1 = 0.0306<br>wR_2 = 0.0607	R_1 = 0.0458<br>wR_2 = 0.0683	R_1 = 0.0291<br>wR_2 = 0.0604	R_1 = 0.0593<br>wR_2 = 0.1257	R_1 = 0.0465<br>wR_2 = 0.0816
Largest peak/hole [eÅ^{-3}]	1.50/−1.23	0.94/−0.61	1.95/−2.44	1.97/−1.12	0.90/−0.61

5. UV-Vis spectroscopy

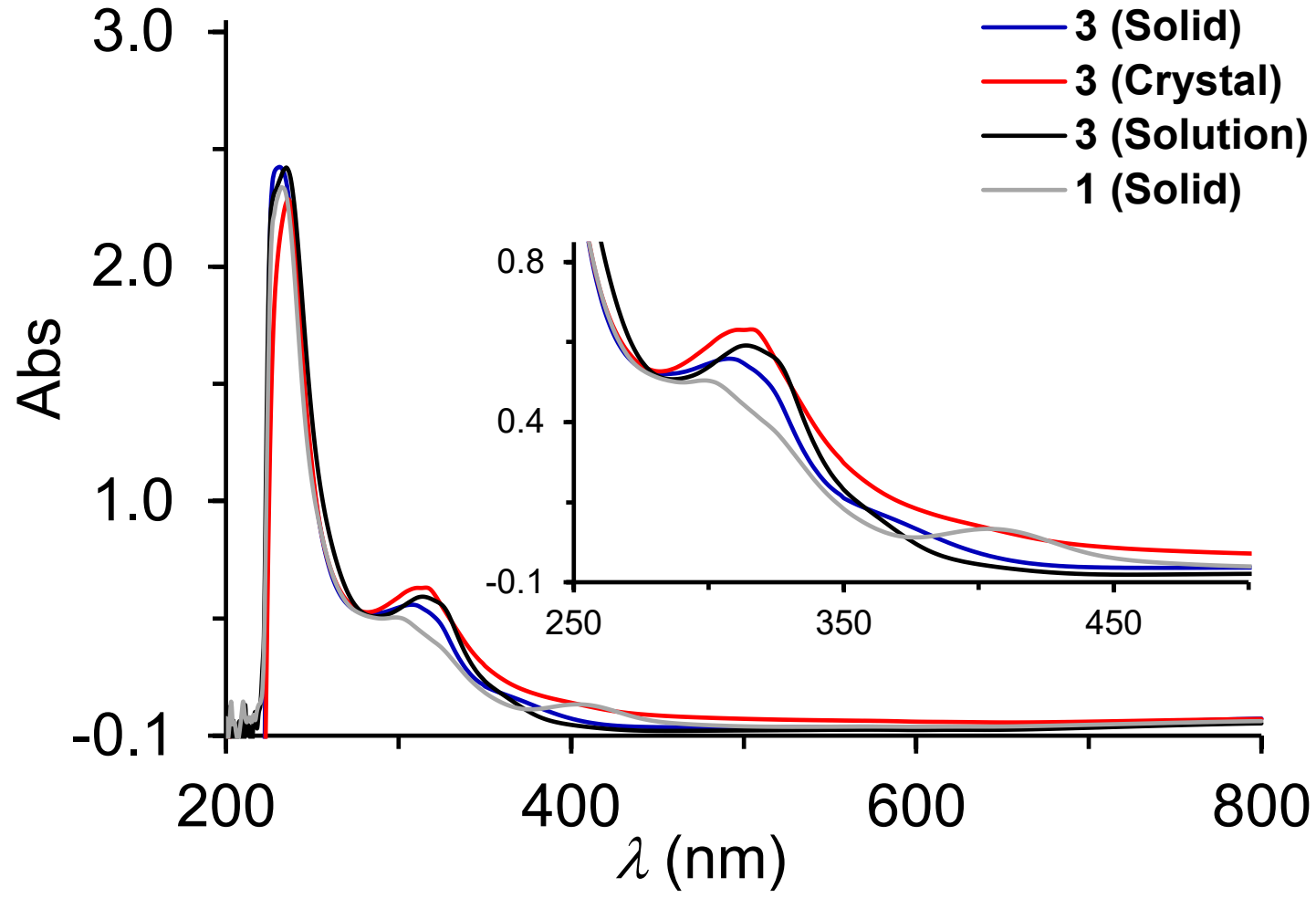

Figure S24. UV-vis spectra of complex (6-Fp)$PtPh_2$ (**1**) and {[(6-Fp)$PtPh_2Ag(OTf)(H_2O)]_2$(*μ*-Ag)}OTf (**3**), in DCM solvent. Note: **3 (Solid)** refers to the isolated solid obtained from the reaction of **1** with 2 equiv of AgOTf; **3 (Crystal)** refers to a single crystal grown from the isolated solid; **3 (Solution)** refers to the solution obtained after addition of 2 equiv of AgOTf to **1** and stirring for 30 minutes.

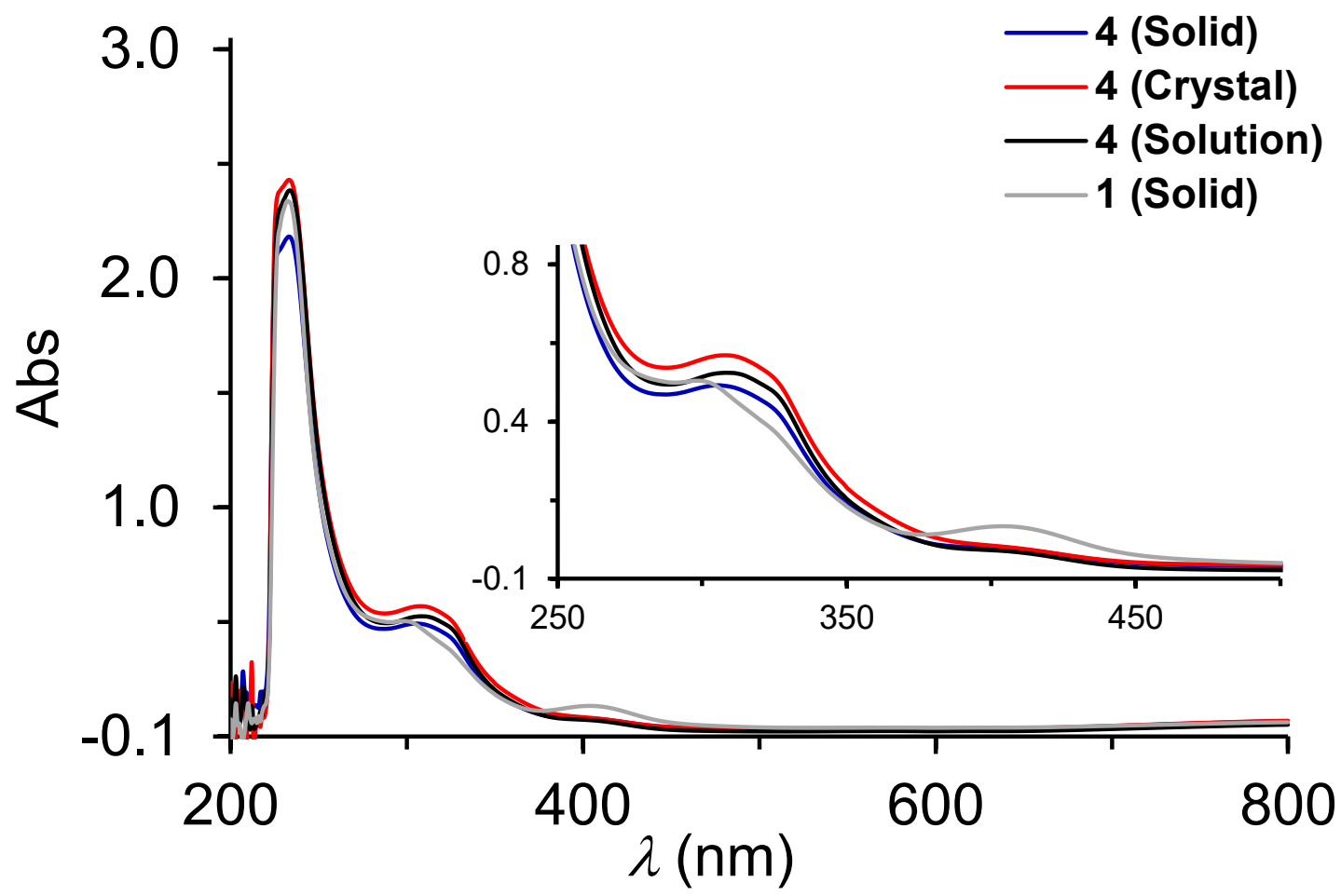

Figure S25. UV-vis spectra of complex (6-Fp)$PtPh_2$ (**1**), and {[(6-Fp)$PtPh_2]_2$(μ-Ag)}NO_3 (**4**) in DCM solvent. Note: **4 (Solid)** refers to the isolated solid obtained from the reaction of **1** with 2 equiv of $AgNO_3$; **4 (Crystal)** refers to a single crystal grown from the isolated solid; **4 (Solution)** refers to the solution obtained after addition of 2 equiv of $AgNO_3$ to **1** and stirring for 30 minutes.

6. ICP-OES measurement

The relative molar ratio Pt and Ag in the complexes were performed by ICP-OES using a PerkinElmer Avio 200 spectrometer. Approximately 5 mg of each complex was digested in 5 mL of concentrated nitric acid at 70 ºC for 12 h. The digested solution was subsequently diluted to 100 mL with deionized water and filtered through a 0.2 μm PTFE membrane filter before ICP–OES measurement.

Table S4. ICP-OES measurement of the complexes **1**-**4** to quantify the ratios of Pt and Ag.

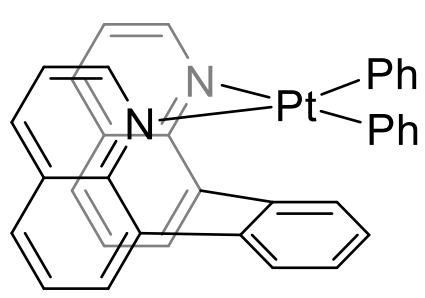

(1)

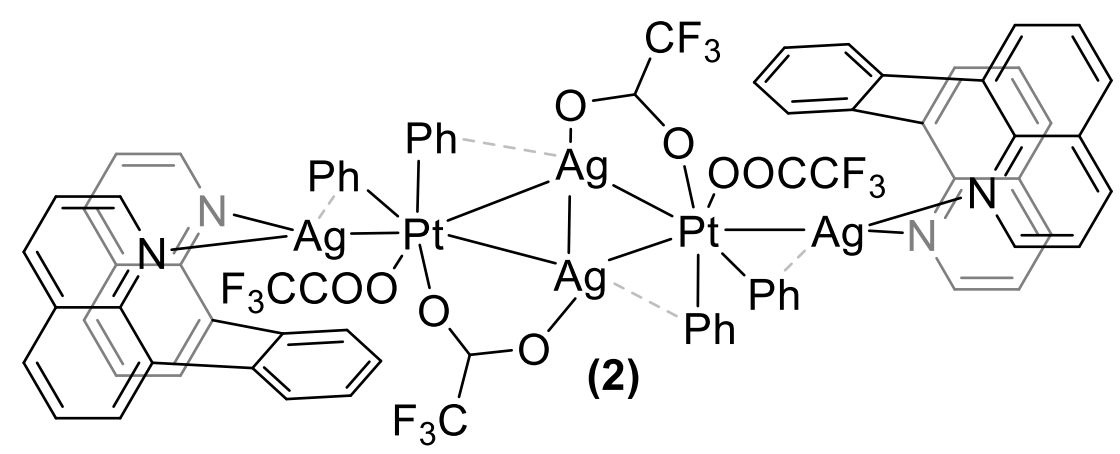

(2)

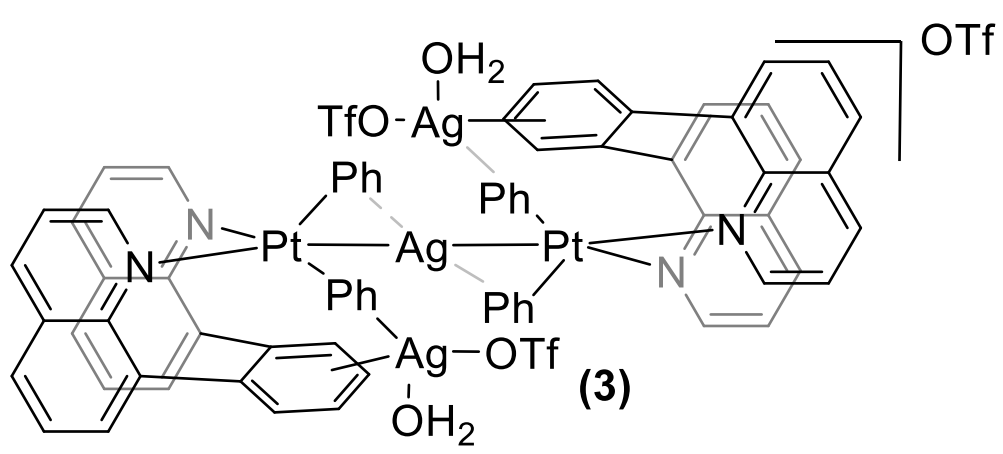

(3)

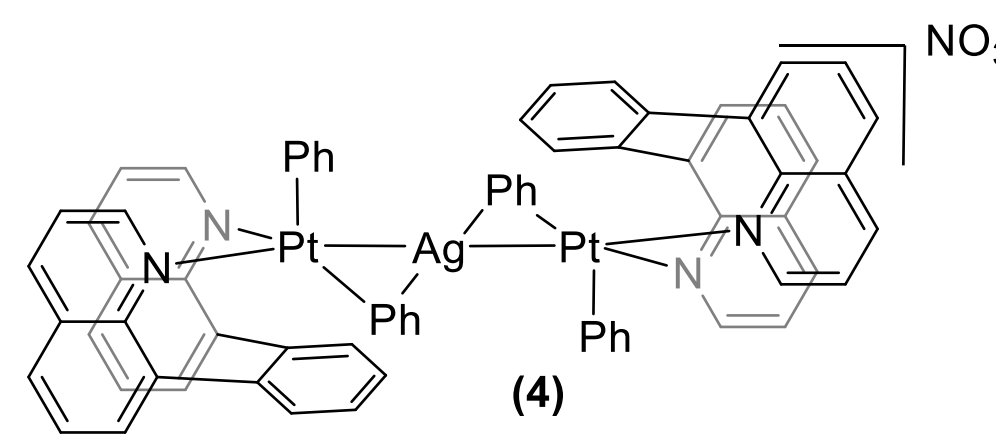

(4)

Complexes	Theoretical Pt:Ag ratio	ICP measured Pt:Ag ratio (Set 1)	ICP measured Pt:Ag ratio (Set 2)
6FpPtPh$_2$ (**1**)	1:0	1:0	-
[[(6-Fp)AgPtPh$_2$(TFA)(μ-TFA)Ag]$_2$ (**2**)	1:2	1:2.802	1:2.771
{[(6-Fp)PtPh$_2$Ag(OTf)(H$_2$O)](μ -Ag)}OTf, (**3**)	1:1.5	1:2.058	1:2.0717
{[(6-Fp)PtPh$_2$]$_2$(μ-Ag)}NO$_3$, (**4**)	1:0.5	1:0.601	1:0.7098

7. Reaction of (6-Fp)PtPh$_2$ (1) with Ag(I) salts at high temperature

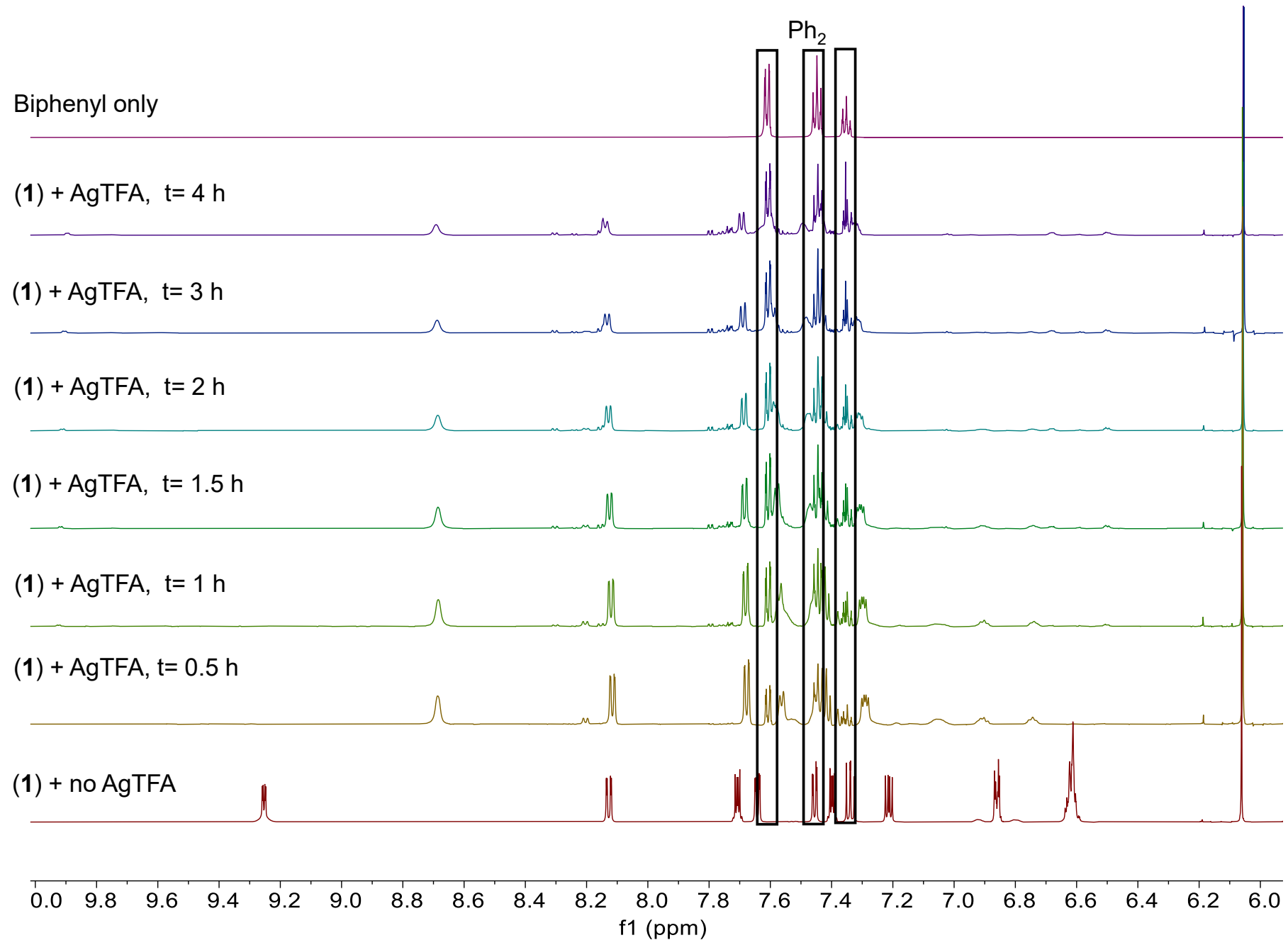

Figure S26.^{1}H NMR spectra for the time course data for the reaction of (6-Fp)PtPh$_2$ (**1**) with 2 equiv of AgTFA in CD_2Cl_2 at 90 ºC.

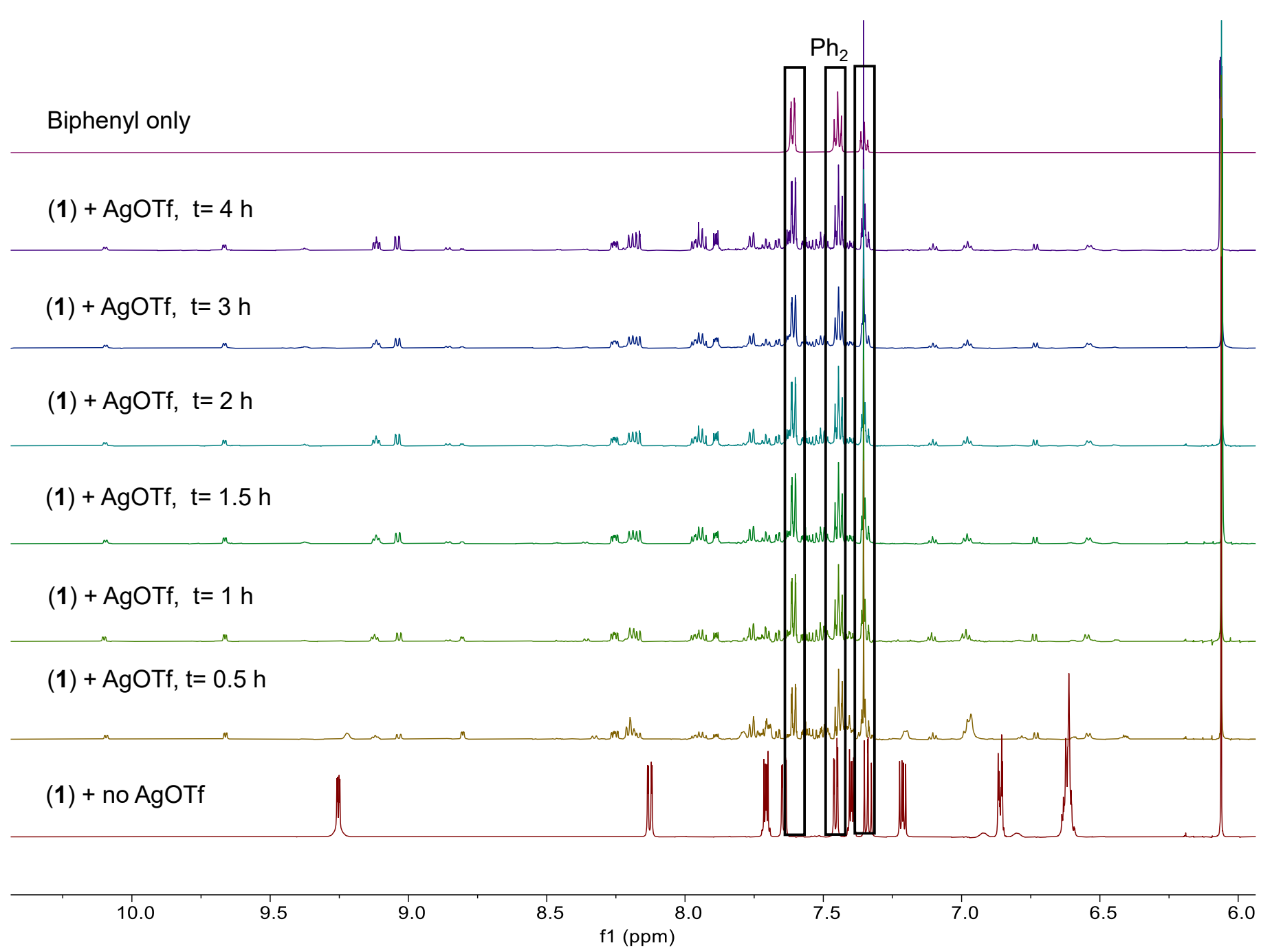

Figure S27.^{1}H NMR spectra for the time course data for the reaction of (6-Fp)PtPh$_2$ (**1**) with 2 equiv of AgOTf in CD_2Cl_2 at 90 ºC.

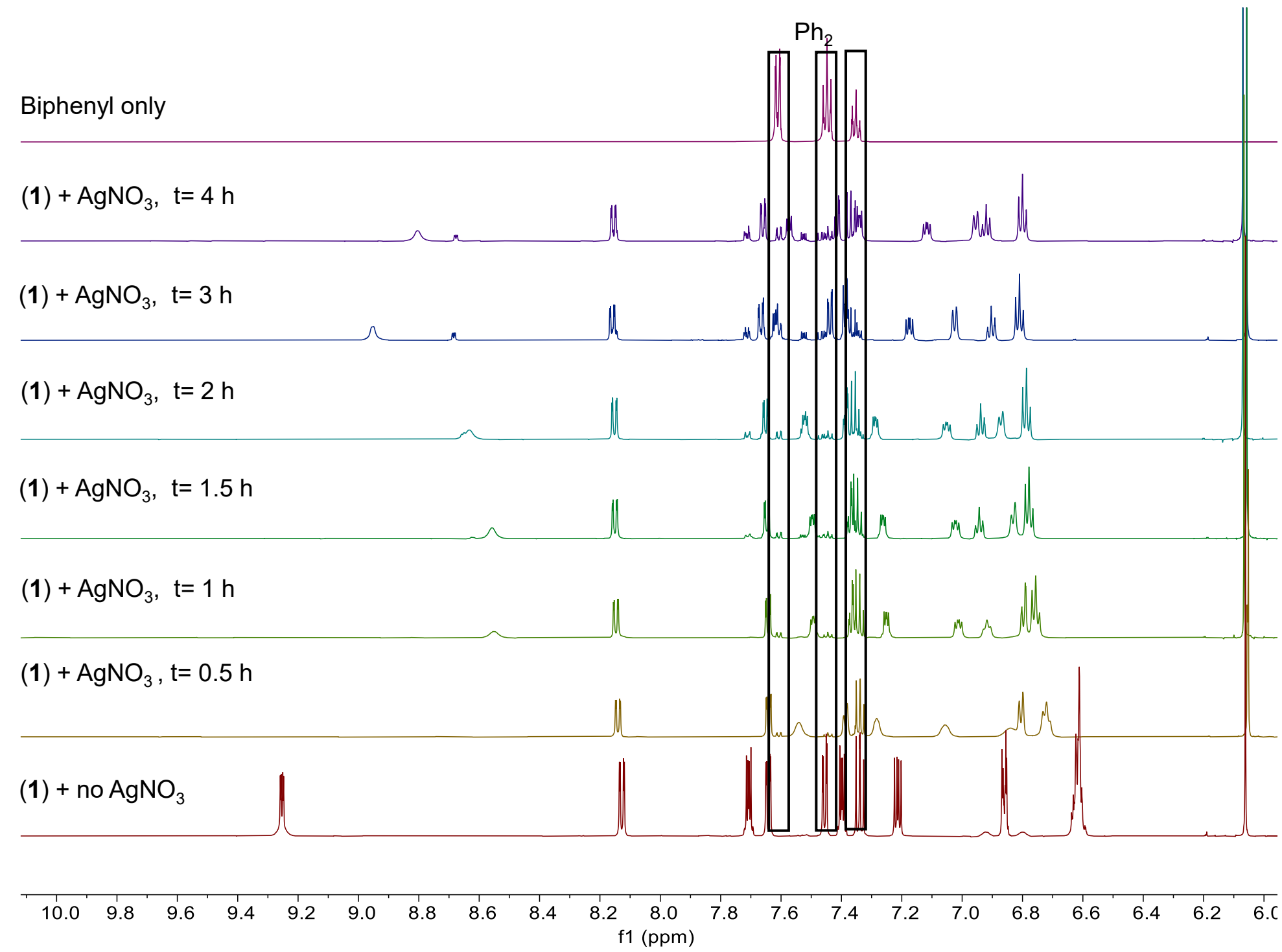

Figure S28.^{1}H NMR spectra for the time course data for the reaction of (6-Fp)PtPh$_2$ (**1**) with 2 equiv of AgNO$_3$ in CD$_2$Cl$_2$ at 90 ºC.

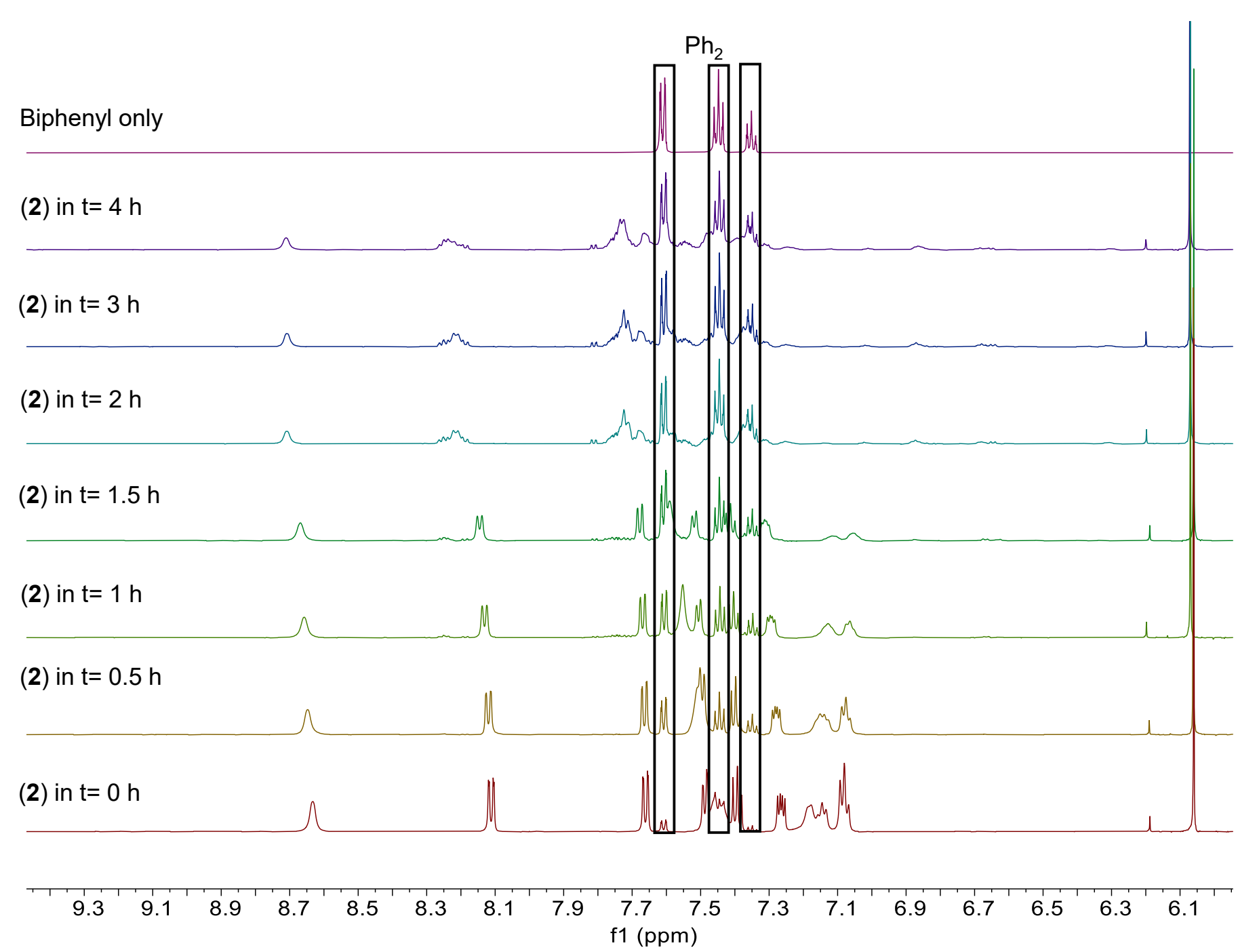

Figure S29.^{1}H NMR spectra for the time course data for the reaction of [(6-Fp)AgPtPh$_2$(TFA)(μ-TFA)Ag]$_2$ (**2**) CD$_2$Cl$_2$ at 90 ºC.

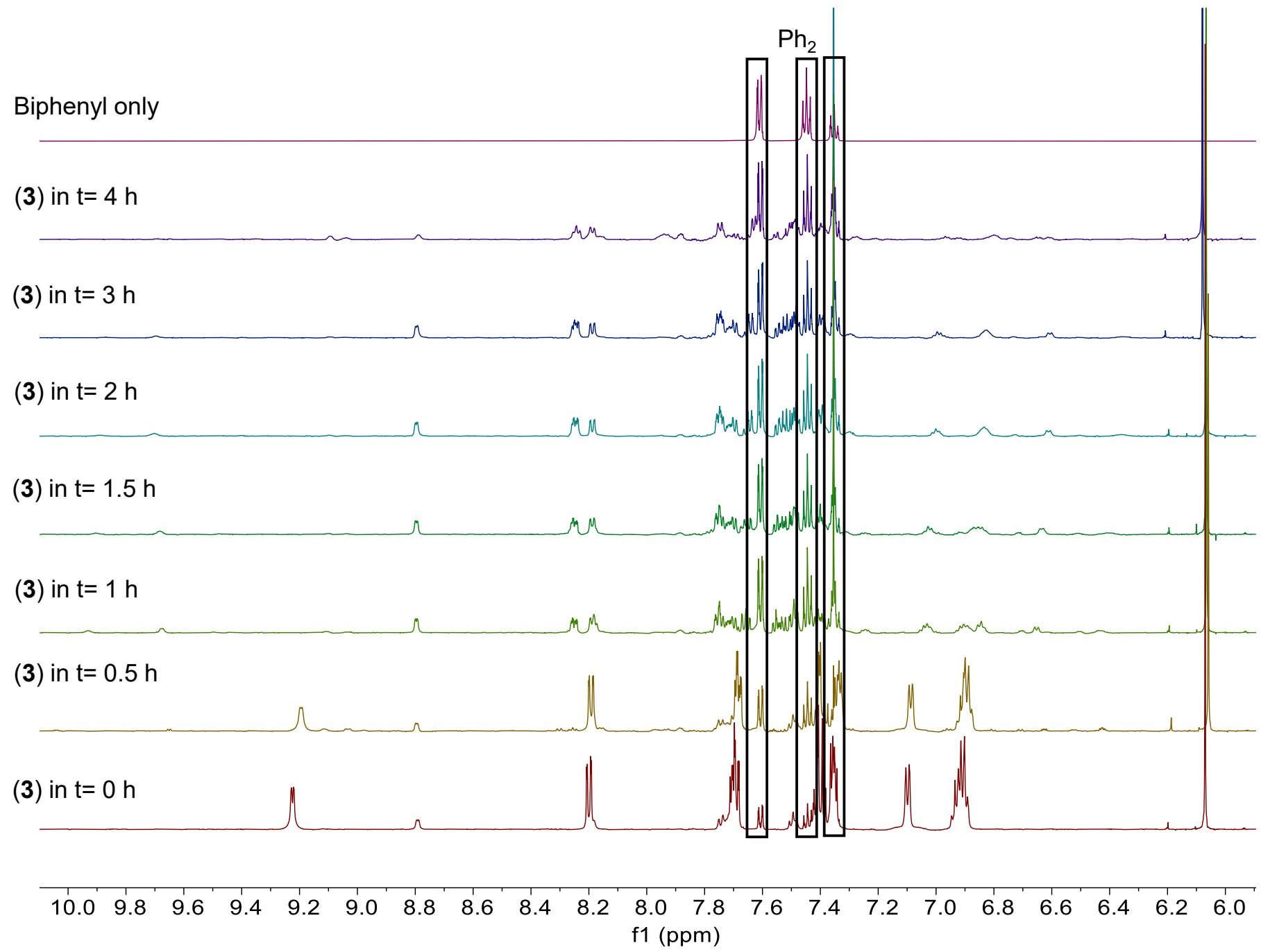

Figure S30.^{1}H NMR spectra for the time course data for the reaction of {[(6-Fp)PtPh$_2$Ag(OTf)(H_2O)]$_2$(*μ*-Ag)}OTf (**3**) in CD_2Cl_2 at 90 ºC.

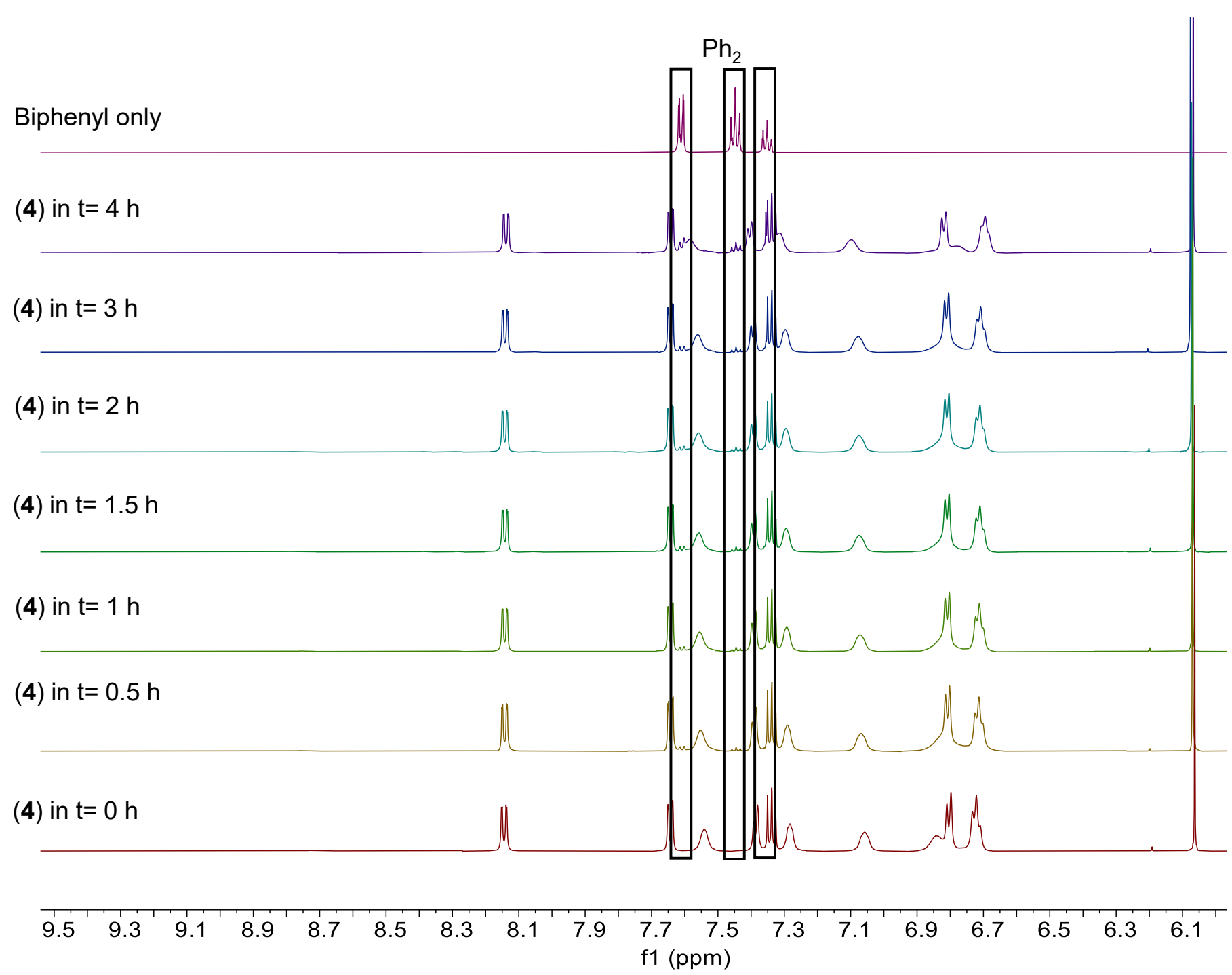

Figure S31.^{1}H NMR spectra for the time course data for the reaction of {[(6-Fp)PtPh$_2$]$_2$(μ-Ag)}NO_3, (**4**) in CD_2Cl_2 at 90 ºC.

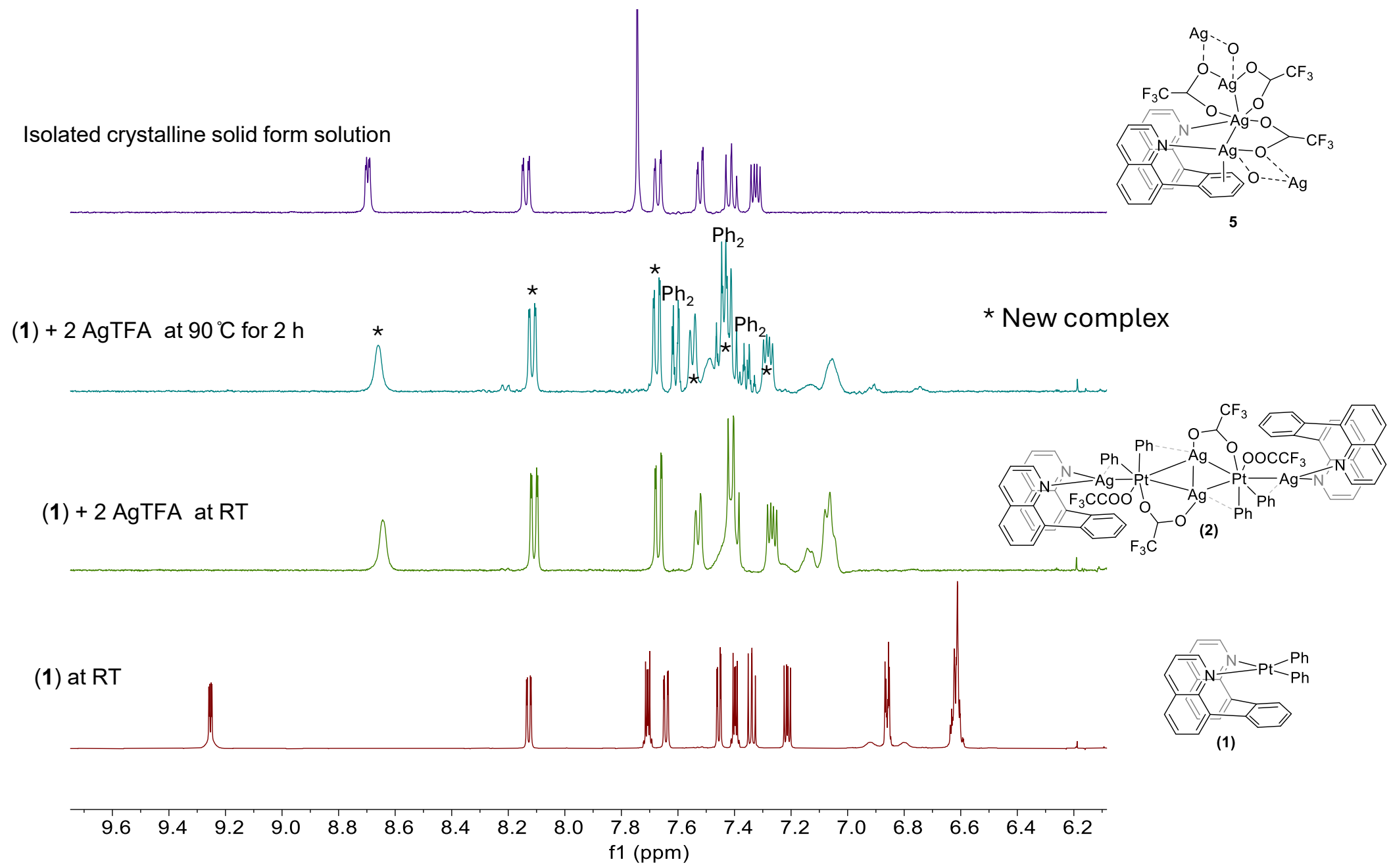

Figure S32. Crude ^{1}H NMR spectra for the reaction of (6-Fp)$PtPh_2$ (**1**) with AgTFA at room temperature and at 90 ºC in CD_2Cl_2.

8. XPS Study

X-ray photoelectron spectroscopy (XPS) was used to characterize a series of samples. The analysis began with the platinum precursor K_2PtCl_4 as a benchmark for Pt 4f peak position, followed by the complex (6-Fp)$PtPh_2$ (**1**). Two products derived from reactions of **1** with silver salts were examined: [(6-Fp)$AgPtPh_2$(TFA)(μ-TFA)Ag]$_2$ (**2**), obtained from the reaction with AgTFA, and {[(6-Fp)$PtPh_2$Ag(OTf)(H_2O)]$_2$(μ-Ag)}OTf (**3**), obtained from the reaction with AgOTf.

In addition, samples from reactions at 90 °C were studied. When **1** was heated with AgTFA, an off-white precipitate formed, designated **2″-ppt** where ppt stands in for "precipitate"; the analogous precipitate from the reaction with AgOTf was labeled **3″-ppt**. Finally, the complete reaction mixtures from the 90 °C reactions with AgTFA and AgOTf were analyzed as **2″-all** and **3″-all**, respectively. **Scheme S2** summarizes the reaction pathways and shows photographs of the corresponding solutions prior to XPS analysis.

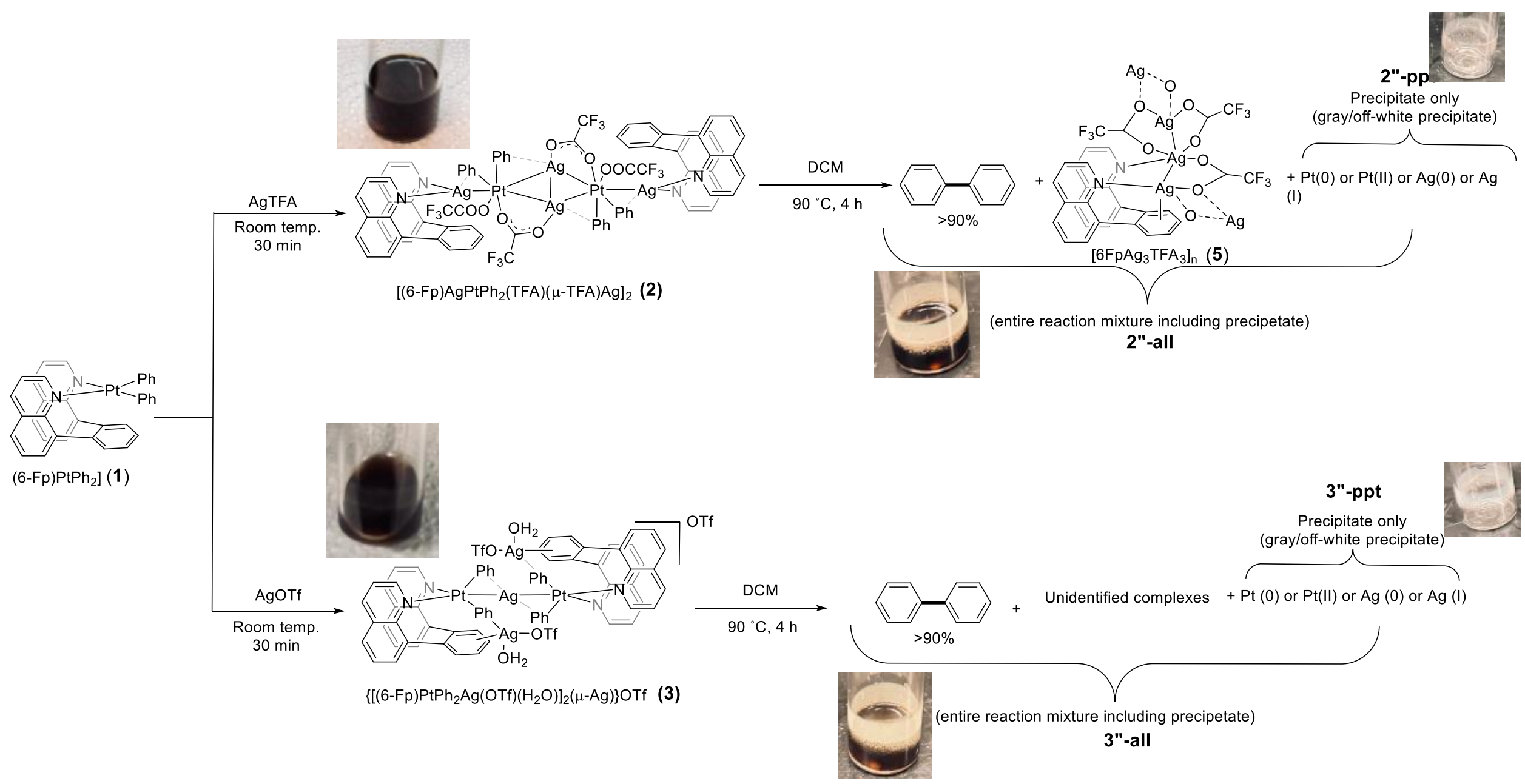

Scheme S2. Reaction scheme and image of the for the solution for XPS analysis

Measurements were taken with a Scienta Omicron Multiprobe MXPS equipped with a monochromatic Al Kα X-ray source (hν = 1486.7 eV), referenced to the Au $4f_{7/2}$ core level at 84.0 eV. Survey and valence band spectra were measured with a pass energy of 80 eV, while individual core levels were recorded with a pass energy of 26.0 eV. The fine-

grained molecular powders were mounted on a strip of carbon tape and densely packed layers. Samples were checked for charging and radiation damage by recording the survey spectra before and after the measurements for each complex. Neither charging nor radiation damage was observed. The Pt 4f, Ag 3d, O 1s, C 1s, and F 1s core levels were recorded, along with the valence band region. The compositions of all complexes were analyzed by XPS and the cation ratios are summarized in **Table S5**. The complexity of the bonding configurations did at present not support a meaningful analysis using fit of the core levels.

Table S5. Composition of compounds, mixtures, and precipitate from XPS.

Pt %	Ag %	Samples names	formula
100%	0%	**K_2PtCl_4**	
100%	0%	**1**	
25%	75%	**2**	2Pt - 4Ag
26%	74%	**2"-all**	0Pt - 5Ag
0%	100%	**2"-ppt**	
37%	63%	**3**	2Pt – 4Ag
65%	35%	**3"-all**	
3%	97%	**3"-ppt**	

The Pt:Ag ratios are calculated from analysis of the XPS data and use the respective core level area adjusted with the respective cross section[13]. Pt:Ag ratios provide a check on the expected stoichiometries. As anticipated, K_2PtCl_4 and **1** contain only platinum. Complex **2** shows a Pt:Ag ratio of 25:75, in reasonable agreement (within experimental error) with the expected 2Pt:4Ag stoichiometry, while complex **3** (37:63) matches the same expected ratio well. The heated reaction mixture **2″-all** gives a similar ratio (26:74), whereas its precipitate **2″-ppt** contains no detectable Pt at all. In contrast, **3″-all** shows an unexpectedly Pt-rich composition (65:35) that does not correspond to any identified

structure, and **3″-ppt** contains only trace Pt (3:97), confirming that both precipitates are dominated by silver.

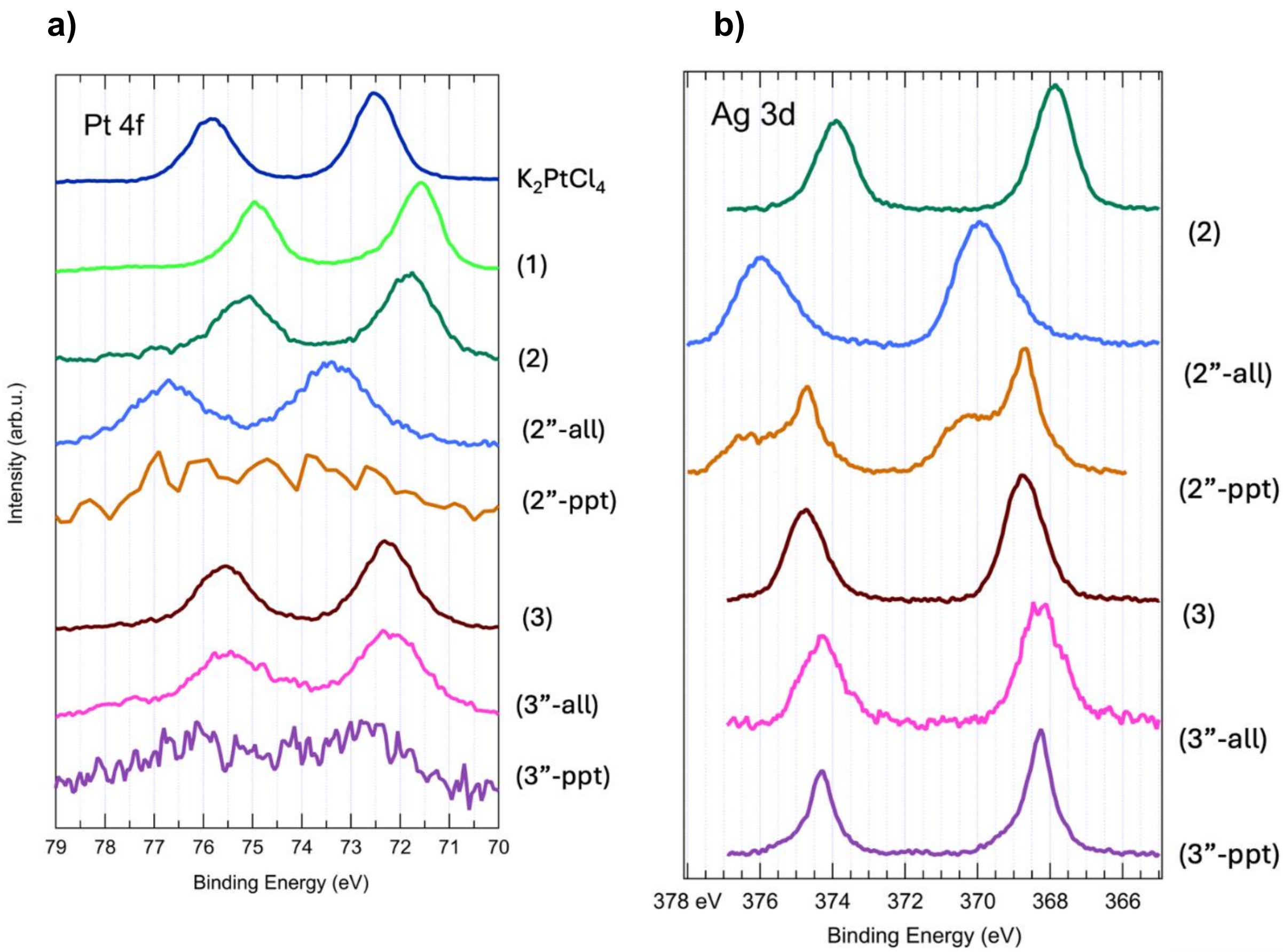

Figure S33: a) Pt 4f and b) Ag 3d core level for compounds for the first reaction sequence with the precursor 6-Fp)$PtPh_2$ (**1**), complex [(6-Fp)$AgPtPh_2$(TFA)(*μ*-TFA)Ag]$_2$ (**2**), reaction product after heating in DCM (**2"-all**), and the precipitate only (**2"-ppt**). The second reaction sequence: complex {[(6-Fp)$PtPh_2$Ag(OTf)(H_2O)]$_2$(*μ*-Ag)}OTf (**3**), reaction product after heating in DCM (**3"-all**) and the isolated precipitate (**3"-ppt**).

The inorganic salt K_2PtCl_4, part of the synthesis sequence for **1**, is included for comparison; its peak, representative of Pt(II), appears at 72.7 eV, in agreement with the literature.[14] For reference, Pt(0) appears at 71.1 eV. The core levels for **1** and **2** are commensurate with a Pt(I)-like oxidation state, but the presence of Pt at the Pt(II) position in **2″-all** cannot be explained. No Pt is present in the **2″-ppt** precipitate, and only traces are found in **3″-ppt**, confirming that the precipitates are dominated by Ag. The unusual shift for **2″-all** in the Ag 3d and Pt 4f core levels is also reflected in the C 1s and F 1s

spectra and is therefore tentatively attributed to band bending which affects all core levels. The mixture of different compounds in these samples prohibits the use of an internal reference such as the valence band maximum, so an unambiguous assignment cannot be made. The small shifts for Ag 3d also described in the manuscript prohibit a conclusive assignment of oxidation states. Both **2”-all** and **2”-ppt** are relatively broad and two distinct contributions are seen most prominently for 2”-ppt albeit we cannot attribute them with confidence to specific oxidation states.

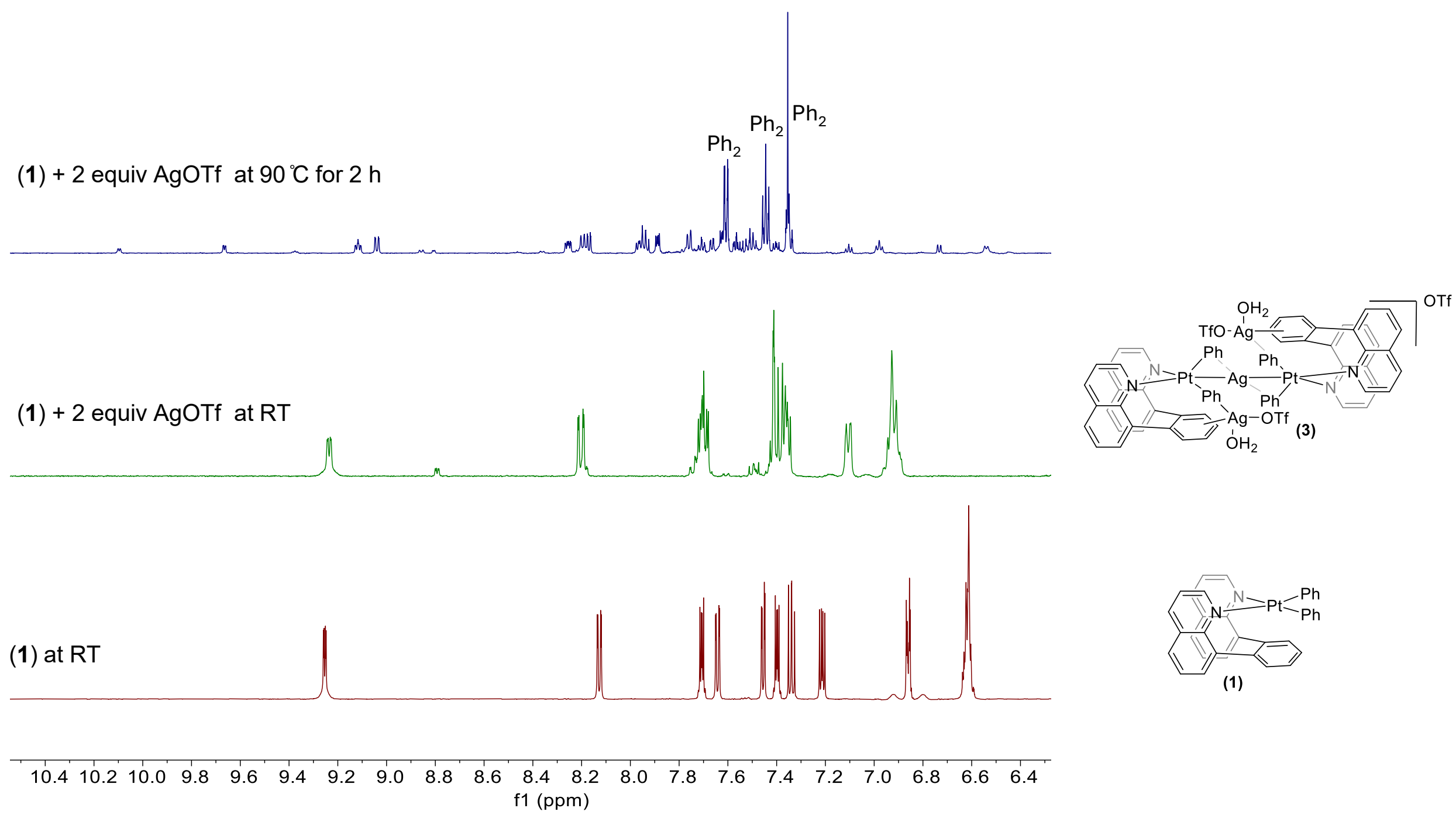

Figure S34. Crude ^{1}H NMR spectra for the reaction of (6-Fp)PtPh$_2$ (**1**) with AgOTf at room temperature and at 90 ºC in CD_2Cl_2.

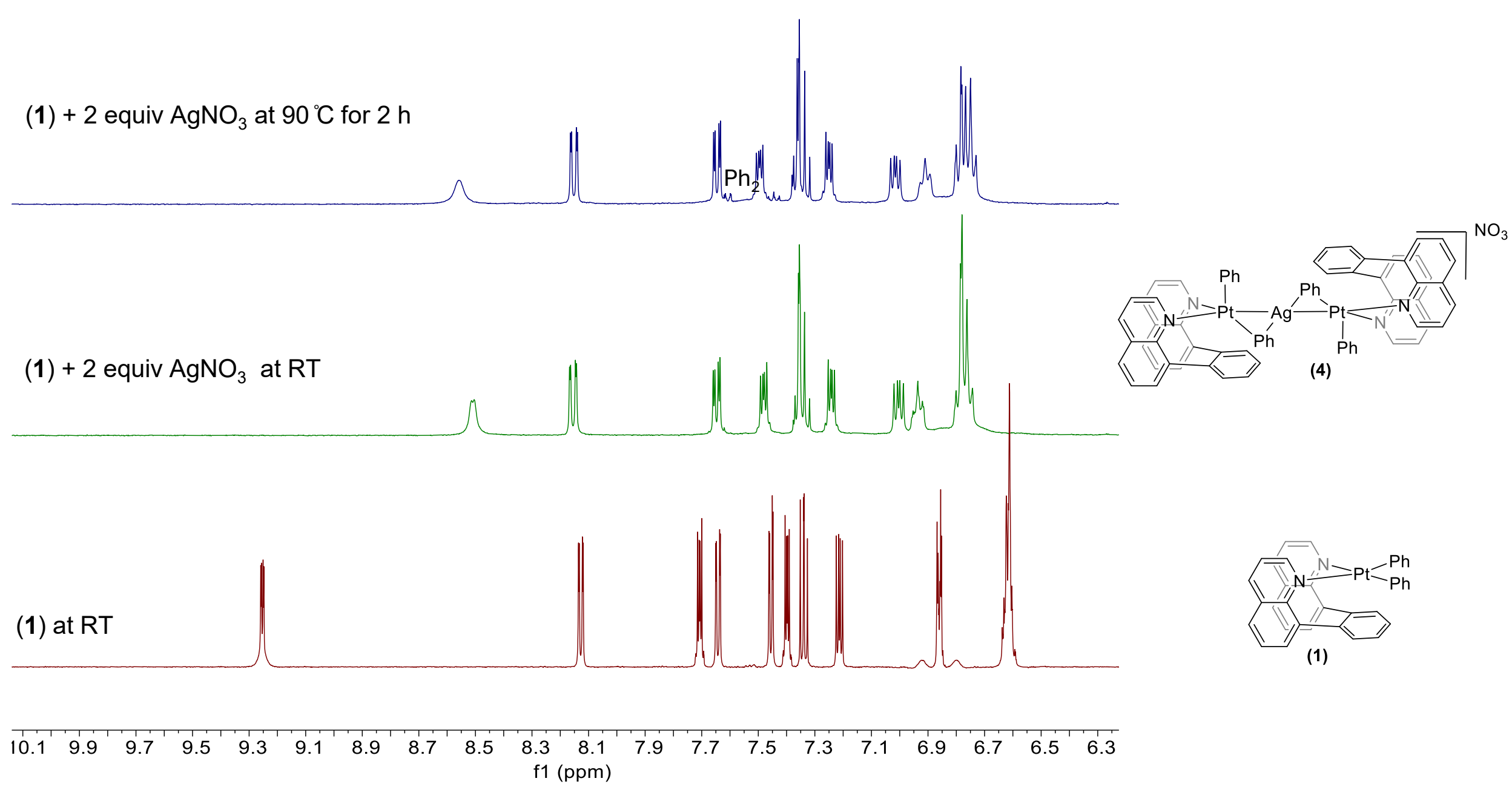

Figure S35. Crude ^{1}H NMR spectra for the reaction of (6-Fp)PtPh$_2$ (**1**) with $AgNO_3$ at room temperature and at 90 ºC in CD_2Cl_2.

9. Isotope scrambling experiment

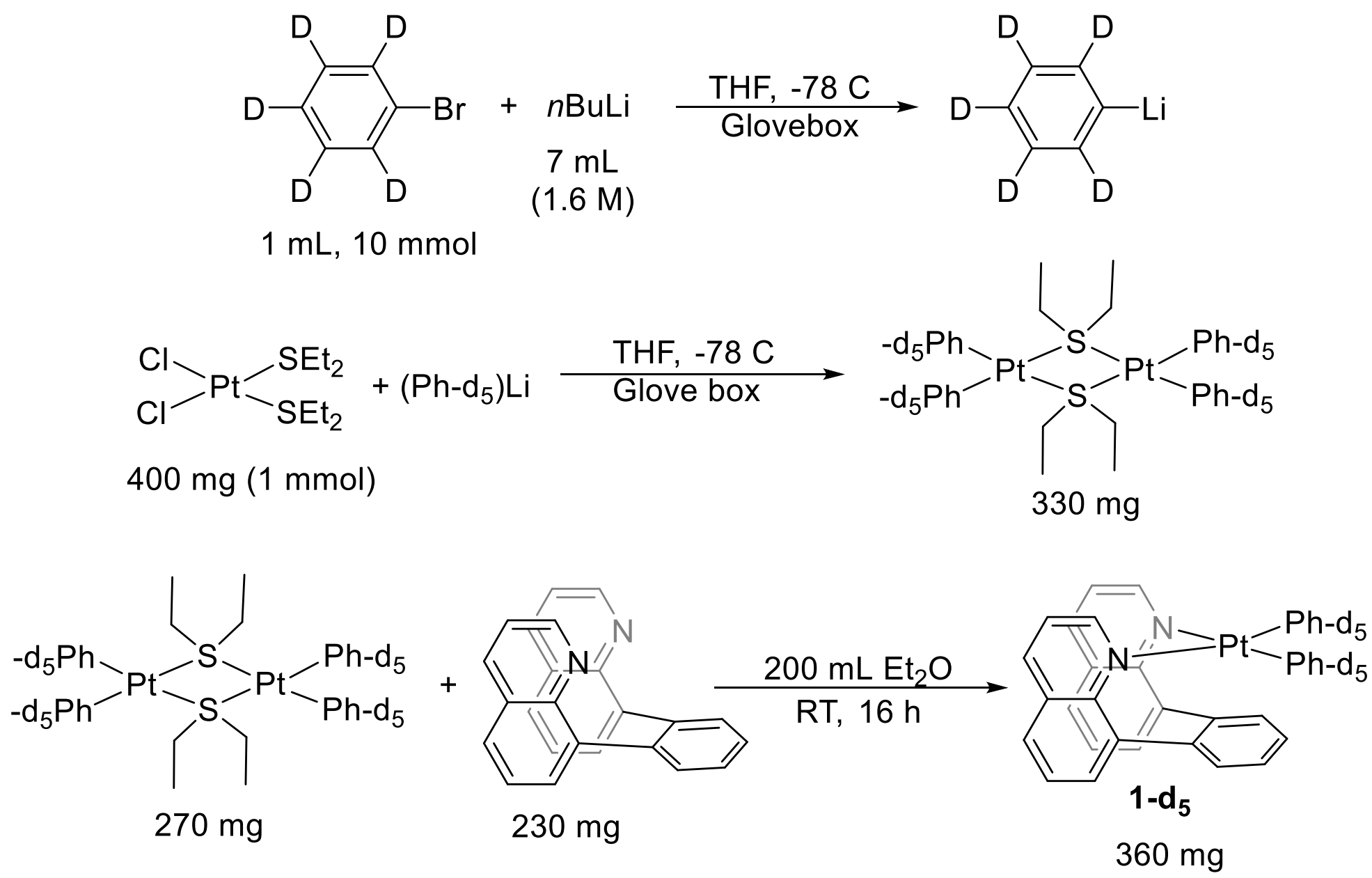

Scheme S3. Reaction scheme for the synthesis of (6-Fp)Pt(Ph-*d*$_5$)$_2$ from $K_2[PtCl_4]$ and *d*$_5$-bromobenzene and the isolated products.

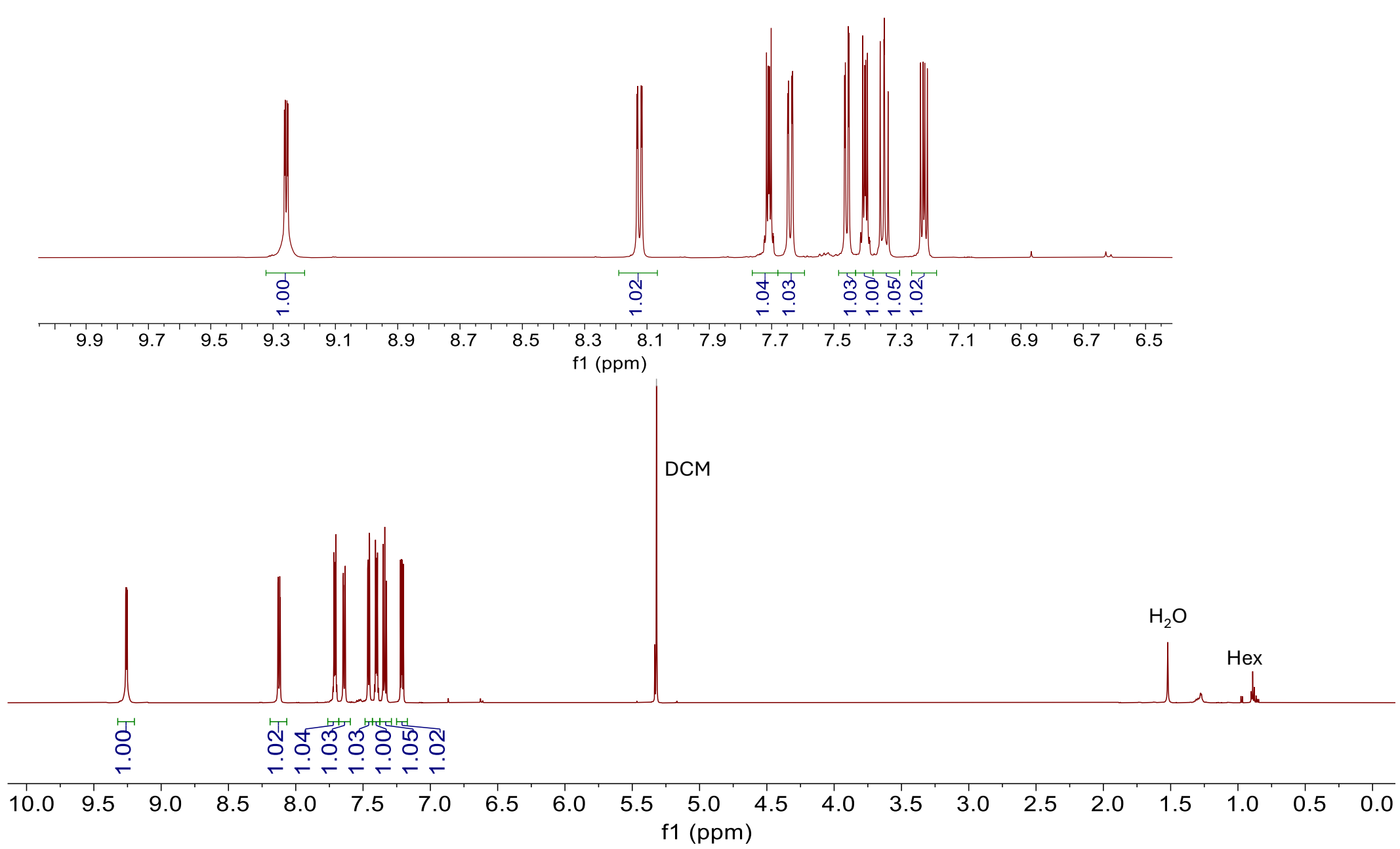

Figure S36. ^{1}H NMR spectrum of (6-Fp)Pt(Ph-*d*$_5$)$_2$ (**1-d$_5$**) in CD_2Cl_2.

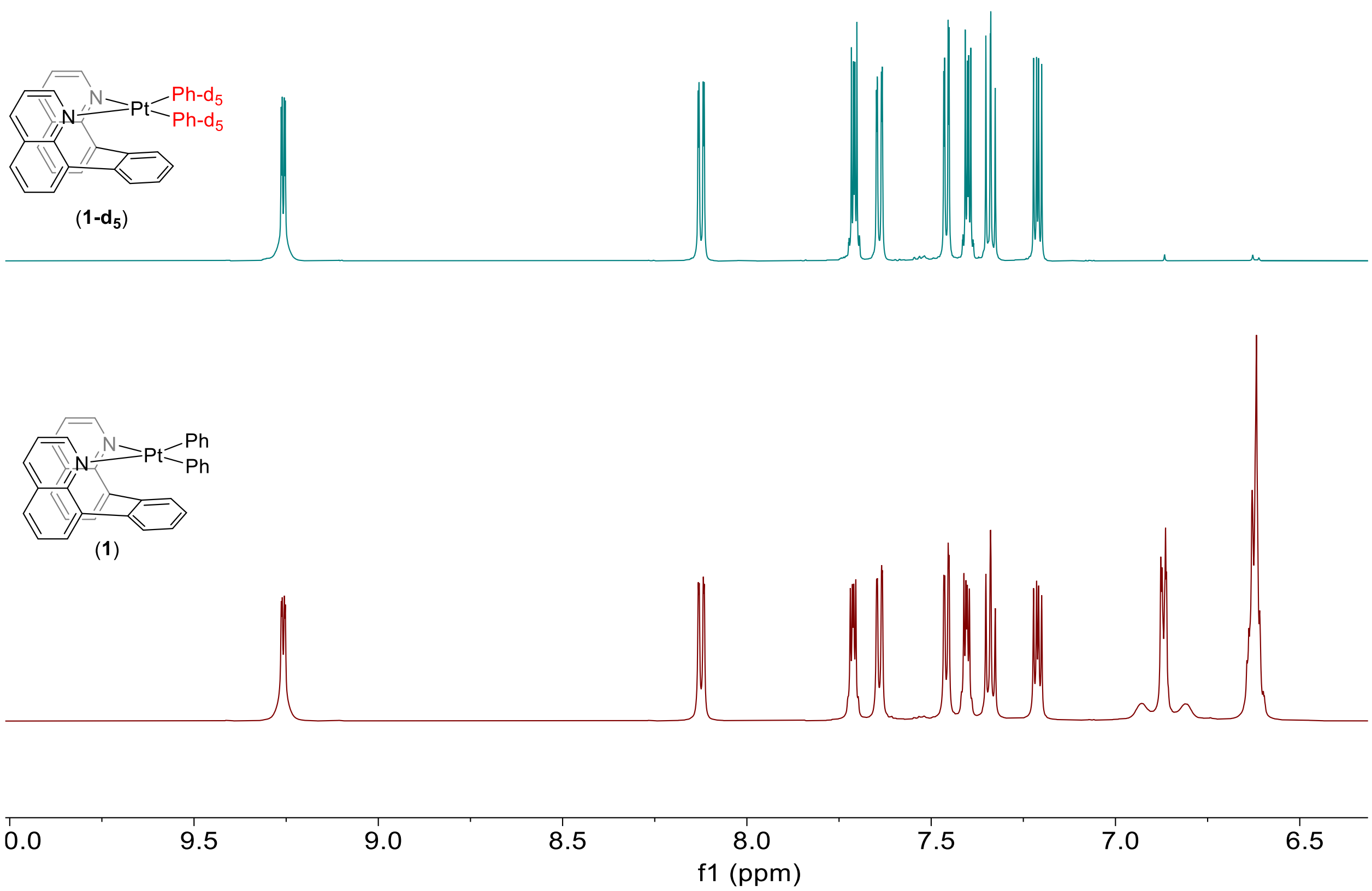

Figure S37. ^{1}H NMR spectra of (6-Fp)Pt(Ph)$_2$ (**1**) and (6-Fp)Pt(Ph-*d*$_5$)$_2$ (**1-d$_5$**) in CD_2Cl_2.

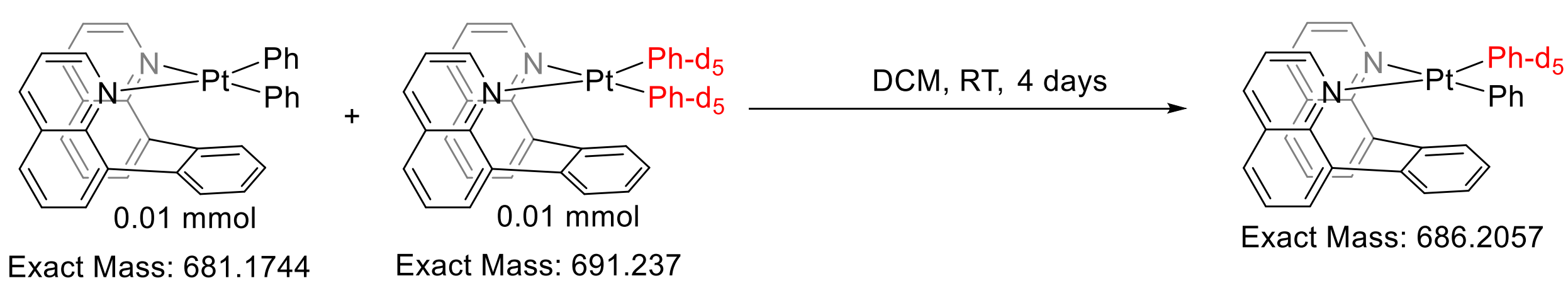

Scheme S4. Isotope scrambling at room temperature.

Note: The 1:1 mixture of (6-Fp)PtPh$_2$ (**1**) and (6-Fp)Pt(Ph-*d*$_5$)$_2$ (**1-*d*$_5$**) in DCM solvent at room temperature for 4 days does not produce any complex with the formula (6-Fp)Pt(Ph)(Ph-*d*$_5$) as indicated by ESI-MS analysis. This is consistent with no phenyl scrambling occurring at room temperature.

10. Computational Study

Computational Methods

DFT calculations were performed using ORCA 6.1.[15] All final DFT geometry optimizations and vibrational frequencies calculations were performed using the M06[16] functional with a basis set of def2-SVP[17] for all atoms with solvation energy corrections. Solvation energy corrections were calculated using the CPCM[18] solvation model with default parameters for dichloromethane. Quasiharmonic corrections for enthalpy and vibrational entropy calculations were applied (at 298.15 K and 1 atm) with a cutoff frequency of 100 cm^{-1} using the GoodVibes[19] software suite. Note that due to the very large and flexible size of these structures, some optimized structures have very small residual imaginary frequencies that were inverted to be positive real modes.

GOAT-explore was also run using ORCA 6.1 with the semiempirical model g-xTB[20]. The GOAT-explore algorithm enforced a minimum number of optimizations (max(3N, 45)) due to the increased degrees of freedom withing this looser topology space. The default parameters for ORCA's "LOOSEOPT" setting were used due to the extreme cost of running ~60,000-80,000 optimizations per intermediate.

Probing the Configurational Space with GOAT-Explore

Due to the large number of possible metal configurations and the generally vast configurational space of large metal complexes we chose to use a more aggressive version of ORCA's Global Optimization Algorithm (GOAT) called GOAT-explore, which does not enforce topological constraints. We found that there was only a small deviation in RMSD from the located global minima and the corresponding DFT optimized X-ray structures, with heavy atom RMSDs of 1.75 Å for **2**, 2.04 Å for **3**, and 0.26 Å for **4**. In comparing the qualitative changes between the M06 structures and the g-xTB global minima we found that the structure of complex **2** had a slightly altered coordination of ligands to Pt, **3** had rotations of the triflate and water ligands, and **4** had virtually no change at all. Note that we are using the second lowest minima configuration for **3** rather than the g-xTB-computed global minimum since the global minimum may not be directly connected to the transition state and would likely have to lose many of its stabilizing

interactions during the reductive elimination of biphenyl, though it is still only moderately different from the DFT optimized X-ray structure (RMSD = 2.49 Å). See the XYZ coordinate file for the structures of g-xTB computed global minima.

Our confidence in these results was further reinforced by our subsequent computations where we took a relatively unstable complex with alternative Pt/Ag topologies from each GOAT-explore calculation and fed it back into a new GOAT-explore run. The lowest energy structures from those runs were less stable than the DFT optimized x-ray structures and their closely related g-xTB computed global minima.

M06 Energies, Thermal Corrections, and Imaginary Vibrational Modes

Table S6. Energies at the M06/def2-SVP/CPCM(DCM) level of theory including all small imaginary modes (i.e., <100 cm^{-1}) that were converted to real modes during QH-correction. All energies are in Hartree.

Complex	Energy	Enthalpy	Free Energy	QH | Enthalpy	QH | Free Energy	Imaginary Frequencies
**2**	-5919.235308	-5917.993445	-5918.260553	-5918.019126	-5918.251643	-18.4, -13.1, -10.0
**3**	-5743.539152	-5742.313212	-5742.556175	-5742.33537	-5742.548525	-11.8
**4**	-3375.349546	-3374.261033	-3374.438108	-3374.274889	-3374.433249	-16.9, -11.2, -8.0
**TS-2**	-5919.203402	-5917.961545	-5918.225131	-5917.987016	-5918.217360	-230.9, -41.4, -29.7, -24.8, -16.3, -9.9, -3.9
**TS-3**	-5743.513941	-5742.289228	-5742.526459	-5742.310496	-5742.520785	-239.5, -11.2
**TS-4**	-3375.288992	-3374.202058	-3374.378245	-3374.216272	-3374.374088	-270.8, -10.4

11. References

1. Gu, S.; Musgrave, C. B.; Gehman, Z. M.; Zhang, K.; Dickie, D. A.; Goddard, W. A.; Gunnoe, T. B. Rhodium and Iridium Complexes Bearing “Capping Arene” Ligands: Synthesis and Characterization. *Organometallics* **2021**, *40*, 2808−2825.

2. Otto, S.; Roodt, A. Reactivity Studies of *Trans*-[PtClMe(SMe_2)$_2$] towards Anionic and Neutral Ligand Substitution Processes. *J. Organomet. Chem*. **2006**, *691*, 4626−4632

3. Steele, B. R.; Vrieze, K. Complexes with Metal-carbon Bonds, part III(1). Preparation and Properties of Binuclear Diarylplatinum(II) Compounds.*Transition Met. Chem.* **1977**, *2*, 140–144.

4 Fulmer, G. R.; Miller, A. J. M.; Sherden, N. H.; Gottlieb, H. E.; Nudelman, A.; Stoltz, B. M.; Bercaw, J. E.; Goldberg, K. I. NMR Chemical Shifts of Trace Impurities: Common Laboratory Solvents, Organics, and Gases in Deuterated Solvents Relevant to the Organometallic Chemist. Organometallics 2010, 29, 2176−2179

5. a) Gracia, C.; Marco, G.; Navarro, R.; Romero, P.; Soler, T.; Urriolabeitia, E. P. C-P and C-H Bond Activations and C-C Coupling in Bis-Phosphonium Salts Induced by Platinum(II) Complexes. *Organometallics* **2003**, *22*, 4910−4921. b) Janzen, D. E.; Mehne, L. F.; VanDerveer, D. G.; Grant, G. J. Cationic Five-Coordinate PtII Complexes as Donors in the Formation of Pt→Ag Dative Bonds. *Inorg. Chem*. **2005**, *44*, 8182−8184.

6. Harris, R. K.; Becker, E. D.; Cabral De Menezes, S. M.; Goodfellow, R.; Granger, P. NMR Nomenclature. Nuclear Spin Properties and Conventions for Chemical Shifts (IUPAC Recommendations 2001). *Pure Appl. Chem*. **2001**, *73*, 1795−1818.

7. Bruker (2019). *Saint; APEX6*. Bruker AXS Inc., Madison, Wisconsin, USA

8. Krause, L.; Herbst-Irmer, R; Sheldrick, G. M.; Stalke, D., Comparison of Silver and Molybdenum Microfocus X-ray Sources for Single-Crystal Structure Determination. *J. Appl. Cryst.* **2015**, *48*, 3-10. doi:10.1107/ S1600576714022985

9. Dolomanov, O. V.; Bourhis, L. J.; Gildea, R. J.; Howard, J. A. K.; Puschmann, H., OLEX2: a Complete Structure Solution, Refinement and Analysis Program. *J. Appl. Cryst.* **2009**, ***42***, 339-341. doi:10.1107/S0021889808042726

10. Sheldrick, G. M., SHELXT – Integrated Space-group and Crystal Structure Determination. *Acta Crystallogr. Sect. A: Found. Adv.* **2015**, *A71*, 3-8. doi:10.1107/S2053273314026370

11. Sheldrick, G. M., Crystal Structure Refinement with SHELXL. *Acta Crystallogr. Sect C: Struct. Chem.* **2015**, *C71*, 3-8. doi: 10.1107/S2053229614024218

12. Kratzert, D. FinalCif, https://dkratzert.de/finalcif.html.

13. Scofield, J. H. *Theoretical Photoionization Cross Sections from 1 to 1500 keV*.; UCRL-51326; Lawrence Livermore Laboratory, 1973

14. a) NIST X-ray Photoelectron Spectroscopy Database, NIST Standard Reference Database Number 20, National Institute of Standards and Technology, Gaithersburg MD, 20899 (**2000**), DOI: https://dx.doi.org/10.18434/T4T88K, (retrieved [06/07/2026]). b) Lee, A.Y., Powell, C.J., Gorham, J.M., Morey, A., Scott, J.H.J. and Hanisch, R.J. (2024) "Development of the NIST X-ray Photoelectron Spectroscopy (XPS) Database, Version 5", *Data Science Journal*, **2024**, *45*, pp. 1–9. DOI:https://dx.doi.org/10.5334/dsj-2024-045

15. a) Neese, F. The ORCA Program System. *Wiley Interdiscip. Rev.: Comput. Mol. Sci*. **2012**, *2*, 73−78. b) Neese, F. Software Update: The ORCA Program System. Version 6.0. *Wiley Interdiscip. Rev.: Comput. Mol. Sci.* **2025**, *15*, No. e70019

16. Zhao, Y.; Truhlar, D. The M06 Suite of Density Functionals for Main Group Thermochemistry, Thermochemical Kinetics, Noncovalent Interactions, Excited States, and Transition Elements: Two New Functionals and Systematic Testing of Four M06-Class Functionals and 12 Other Functionals. *Theor. Chem. Acc*. **2008**, *120*, 215−241

17. Weigend, F.; Ahlrichs, R. Balanced Basis Sets of Split Valence, Triple Zeta Valence and Quadruple Zeta Valence Quality for H to Rn: Design and Assessment of Accuracy. *Phys. Chem. Chem. Phys*. **2005**, *7*, 3297−3305.

18. Barone, V.; Cossi, M. Quantum Calculation of Molecular Energies and Energy Gradients in Solution by a Conductor Solvent Model. *J. Phys. Chem. A* **1998**, *102*, 1995−2001.

19. Luchini, G.; Alegre-Requena, J.; Funes-Ardoiz, I.; Paton, R. GoodVibes: Automated Thermochemistry for Heterogeneous Computational Chemistry Data [version 1; peer review: 2 approved with reservations]. *F1000Research* **2020**, *9*, 291.

20. Froitzheim, T.; Müller, M.; Hansen, A.; Grimme, S. g-xTB: A General-Purpose Extended Tight-Binding Electronic Structure Method for the Elements H to Lr (Z = 1−103). *ChemRxiv* **2025**, 2025-bjxvt.
